# NF-κB/RelA signaling required for CD40-induced humoral immunity depends on specific NEMO lysine residues in mice

**DOI:** 10.1038/s44318-026-00875-0

**Published:** 2026-07-30

**Authors:** Donna Li, Shelly M Wuerzberger-Davis, Yuhong Chen, Funita P Phan, Mei Yu, Dominique N Lisiero, Cynthia C Aguilar, Thomas Cleven, Melba M Tejera, Parth Khatri, Natalie Medina, Yu-Chia Chen, Ning Wang, Philippos Tsourkas, Irene Ong, Jeffrey J Bednarski, Huy Q Dinh, Alexander Hoffmann, Barry P Sleckman, Aussie Suzuki, Avtar Roopra, Marulasiddappa Suresh, Demin Wang, Shigeki Miyamoto

**Affiliations:** 1 McArdle Laboratory for Cancer Research, Department of Oncology, Carbone Cancer Center, University of Wisconsin-Madison, Madison, WI USA; 2 Versiti Blood Research Institute, Milwaukee, WI USA; 3 Department of Pathobiological Sciences, University of Wisconsin-Madison, Madison, WI USA; 4 Biostatistics and Medical Informatics, University of Wisconsin-Madison, Madison, WI USA; 5 Department of Microbiology, Immunology, and Molecular Genetics, and Institute for Quantitative and Computational Biosciences, University of California, Los Angeles, Los Angeles, CA USA; 6 Department of Pediatrics, Washington University School of Medicine, St. Louis, MO USA; 7 Department of Medicine - Hematology & Oncology, The University of Alabama at Birmingham School of Medicine, Birmingham, AL USA; 8 Department of Neuroscience, 5507 WIMR, University of Wisconsin-Madison, Wisconsin Institute for Medical Research, Madison, WI USA

**Keywords:** Immunology, Post-translational Modifications & Proteolysis, Signal Transduction

## Abstract

Oxidative stress and genotoxic damage activate NF-κB signaling through intracellular pathways distinct from those initiated by membrane receptors. DNA damage selectively induces post-translational modifications at lysine residues 277 and 309 of the human ubiquitin-binding protein NEMO to promote NF-κB signaling, but the physiological importance of these modifications in vivo remains unclear. Here, we show that a newly developed mouse model (NEMO^DK^) carrying germline arginine substitutions of the corresponding NEMO lysine residues exhibits B-cell-intrinsic defects in germinal center formation and anti-viral humoral responses. Mechanistically, we identify in NEMO^DK^ B-cells a CD40-specific NF-κB signaling defect that is not linked to the well-characterized canonical or noncanonical NF-κB pathways. These B-cells fail to secure sustained NEMO monoubiquitination following CD40-induced ROS generation, which specifically reduces downstream RelA (p65) signaling required for the transcriptomic and epigenetic remodeling underlying homotypic B-cell aggregation, cell proliferation, class-switch recombination, and antibody-secreting cell generation. Our results establish a physiological role of murine NEMO K270 and K302 in linking CD40 engagement to B-cell responses through enabling sustained NEMO modification and RelA transcriptional activity.

## Synopsis

Post-translational modifications of NEMO lysine residues following CD40 stimulation contribute to the regulation of NF-κB signaling and B-cell differentiation, but their function and physiological importance in vivo are incompletely understood. This work shows that murine NEMO residues K270 and K302 enable sustained activation of the canonical NF-κB pathway in response to CD40-induced ROS generation, enhancing RelA (p65) activity and promoting B-cell functions.

- Germline arginine substitutions of NEMO K270/K302 in mice result in B-cell-intrinsic defects in germinal center and antibody responses.
- NEMO K270/K302 are required for efficient homotypic aggregation, proliferation, class-switch recombination, and antibody-secreting cell differentiation regulated by CD40-induced NF-κB signaling.
- NEMO K270/K302-dependent CD40-activated NF-κB signaling is primarily mediated by the sustained activity of RelA in B-cells.
- NEMO K270/K302 contribute to class-switch recombination by enabling the transcription of AID and NBS1 genes.
- CD40-induced mitochondrial ROS, but not DNA damage, contributes to the monoubiquitination of NEMO K270/K302 and sustained NF-κB signaling for downstream B-cell functions.

## Abstract (web summary)

Murine NEMO K270/K302 enable sustained NF-κB signaling and enhanced RelA activity to promote B-cell functions.

## Graphical abstract

**Figure:**
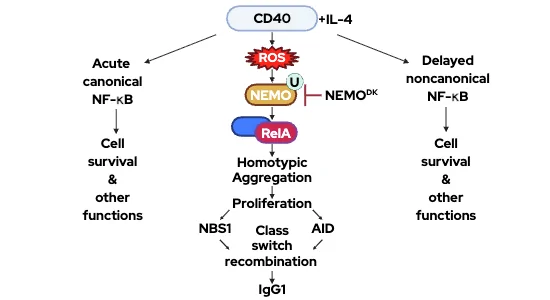

**Figure:**
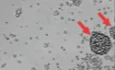

## Introduction

The NF-κB family of transcription factors regulates numerous biological and pathological processes, including development, inflammation, immunity, and cancer (Guo et al, 2024; Taniguchi and Karin, 2018; Zhang et al, 2017). In resting cells, inactive NF-κB complexes are retained in the cytoplasm through association with inhibitor of κB (IκB) proteins. Upon stimulation by diverse signals such as cytokines, pathogenic molecules, oxidative stress, or genotoxic damage, the IκB kinase (IKK) complex is activated. This complex consists of the catalytic subunits IKKα and IKKβ and the regulatory component NEMO (NF-κB essential modulator), also known as IKKγ (Hayden and Ghosh, 2008; Hinz et al, 2010). Activation of the IKK complex results in phosphorylation-dependent degradation of IκB, allowing NF-κB complexes composed of RelA (p65) or cRel with the p50 subunit to translocate into the nucleus and regulate transcription of target genes.

Within the canonical NF-κB pathway, NEMO plays a central role in IKK activation through its ability to bind non-degradative polyubiquitin chains (Hinz and Scheidereit, 2014; Skaug et al, 2009). These ubiquitin chains are generated downstream of several stimuli, including tumor necrosis factor (TNF), Toll-like receptors (TLRs), and DNA damage. In addition to ubiquitin binding, NEMO undergoes numerous stimulus-dependent post-translational modifications (PTMs) that modulate NF-κB signaling (Perkins, 2006; Sebban et al, 2006). Notably, genotoxic stress activates NF-κB through signaling pathways that originate within the cell nucleus rather than from membrane receptors. In this nuclear-to-cytoplasmic pathway, DNA damage triggers NEMO translocation from the cytoplasm to the nucleus, where it undergoes several PTMs including sumoylation, ubiquitination, and phosphorylation by the DNA damage-responsive kinase ATM at serine 85 (S85) (McCool and Miyamoto, 2012). DNA damage-induced NF-κB activation promotes cell survival and has also been implicated in physiological and pathological processes such as B-cell development, cellular senescence, premature aging, and therapy resistance in several cancer types (Janssens and Tschopp, 2006; Konrath et al, 2023; Bredemeyer et al, 2008; Dong et al, 2015; Osorio et al, 2012; Tilstra et al, 2012; Wu et al, 2018).

Reactive oxygen species (ROS) represent another class of intracellular signals capable of modulating NF-κB signaling. However, the effects of ROS on NF-κB activation remain incompletely defined, as both stimulatory and inhibitory effects have been reported depending on the cellular context and experimental conditions (Morgan and Liu, 2011). Notably, exposure to ROS such as H₂O₂ has been shown to activate signaling pathways similar to those triggered by genotoxic stress (Wuerzberger-Davis et al, 2007). Beyond their role in stress responses, ROS also function as important molecules in the immune system. For example, neutrophils and macrophages generate high levels of ROS during the respiratory burst to promote the killing of phagocytosed pathogens (Manoharan et al, 2024). In B lymphocytes, ROS act as secondary messengers that regulate activation, differentiation, and effector functions. In particular, mitochondrial ROS (mROS) have been linked to B-cell fate decisions (Zhang et al, 2019). For example, one study demonstrated that mROS promote class switch recombination (CSR) while suppressing plasma cell differentiation by attenuating heme synthesis and thereby enhancing Bach2 function (Jang et al, 2015).

CD40, a member of the tumor necrosis factor receptor (TNFR) superfamily, is essential for T cell-dependent B cell activation and the generation of robust humoral immune responses (Elgueta et al, 2009; Karnell et al, 2019). Engagement of CD40 by its ligand CD40L (CD154), expressed on activated T follicular helper (T_FH_) cells, initiates intracellular signaling through multiple pathways in B cells (Dadgostar et al, 2002). One of the best-characterized outcomes of CD40 stimulation includes the activation of the NEMO-*dependent* canonical NF-κB pathway and the NEMO-*independent* noncanonical NF-κB pathway (Zarnegar et al, 2004). In the noncanonical pathway, NF-κB-inducing kinase (NIK) activates IKKα homodimers, which mediate proteasomal processing of the p100 precursor into the active p52 subunit. The resulting p52-RelB heterodimers translocate to the nucleus to regulate gene expression (Sun, 2017). Together, these pathways control the activation, proliferation, and survival of CD40-stimulated B cells, as well as homotypic aggregation (HA), immunoglobulin class switch recombination (CSR), and somatic hypermutation (SHM) (Zarnegar et al, 2004).

Consistent with the importance of NEMO in these processes, hypomorphic mutations in NEMO have been linked to impaired CD40-induced B cell activation in patients with X-linked anhidrotic ectodermal dysplasia with immunodeficiency (EDA-ID), including cases associated with osteoporosis and lymphoedema (OL-EDA-ID) (Döffinger et al, 2001; Fusco et al, 2015). Moreover, CD40 signaling is frequently dysregulated in malignancies such as diffuse large B-cell lymphoma and multiple myeloma, and CD40 has therefore emerged as a target for therapeutic intervention (Zhou et al, 2007; Tai et al, 2003; Andersson et al, 2024). Although CD40-induced NEMO polyubiquitination has been reported, the physiological significance of other NEMO PTMs in CD40-mediated NF-κB signaling remains poorly understood (Gerlach et al, 2011; Sasaki et al, 2013).

We previously demonstrated that DNA damage induces sumoylation and monoubiquitination of NEMO in a manner dependent on lysine residues 277 and 309 in human NEMO, leading to NF-κB activation (Huang et al, 2003). Subsequent studies using in vitro cell culture systems have supported a role for NEMO sumoylation in NF-κB signaling induced by various genotoxic agents, as well as oxidative stress induced by H₂O₂ (Hinz et al, 2010; Janssens et al, 2005; Janssens and Tschopp, 2006; Stilmann et al, 2009; Mabb et al, 2006; Wuerzberger-Davis et al, 2007). However, the physiological importance of these PTM sites in vivo has remained unclear. To address this question, we generated a genetically modified mouse model (NEMO^DK^) carrying germline lysine-to-arginine substitutions at the corresponding murine NEMO residues, lysines 270 and 302. Although NEMO^DK^ mice are viable and fertile, we unexpectedly identified B-cell-intrinsic defects in vivo, including reduced GC formation and impaired anti-viral humoral responses. Mechanistically, NEMO^DK^ B cells exhibit a CD40-specific defect in NF-κB signaling that is distinct from the well-characterized canonical and noncanonical NF-κB pathways, as well as DNA damage-ATM-NF-κB signaling. Instead, while mutant B cells generate sustained ROS, in particular mROS, upon CD40 engagement, comparable to wild-type (WT) cells, they fail to induce sustained NEMO monoubiquitination and downstream RelA signaling required for optimal transcriptional and epigenomic remodeling, HA, cell proliferation, CSR and antibody secreting cell (ASC) formation. Together, these findings uncover a physiological role for NEMO K270/K302 in linking CD40 engagement to mROS-mediated NEMO modification and NF-κB activation, thereby promoting T cell-dependent B-cell responses.

## Results

### NEMO^DK^ mice show selective defects in DNA damage-induced NF-κB activation

Our lab previously established the importance of lysine residues 277 and 309 on human NEMO for NF-κB to respond to DNA damage signaling in vitro (Huang et al, 2003). To explore the physiological importance of these residues, we created the NEMO^DK^ mouse model, which harbors germline double lysine (DK) to arginine point mutations at the corresponding NEMO sites (lysine 270 and 302) (Appendix Fig. S1A,B). NEMO^DK^ mice were born without any overt, outward abnormalities, aside from smaller testes in males (Appendix Fig. S1C,D). Yet, hemizygous NEMO^DK^ male mice can breed normally when mated with heterozygous females (Appendix Fig. S1E). Immunoblot analysis also showed similar levels of NEMO, along with some of the NF-κB signaling components, in WT and NEMO^DK^ tissues, although their levels varied widely among tissues (Appendix Fig. S1F). NF-κB activation by TNF${{{\rm{\alpha }}}}$, interleukin (IL)-1${{{\rm{\beta }}}}$, and lipopolysaccharide (LPS) was also normal in NEMO^DK^ mouse embryonic fibroblasts (MEFs) relative to WT controls (Appendix Fig. S1G). The normal TNFα-induced NF-κB activation in NEMO^DK^ MEFs is consistent with the lack of embryonic lethality in NEMO^DK^ mice, unlike the TNF-dependent embryonic lethality of NEMO knockout mice (Rudolph et al, 2000).

**Figure 1:**
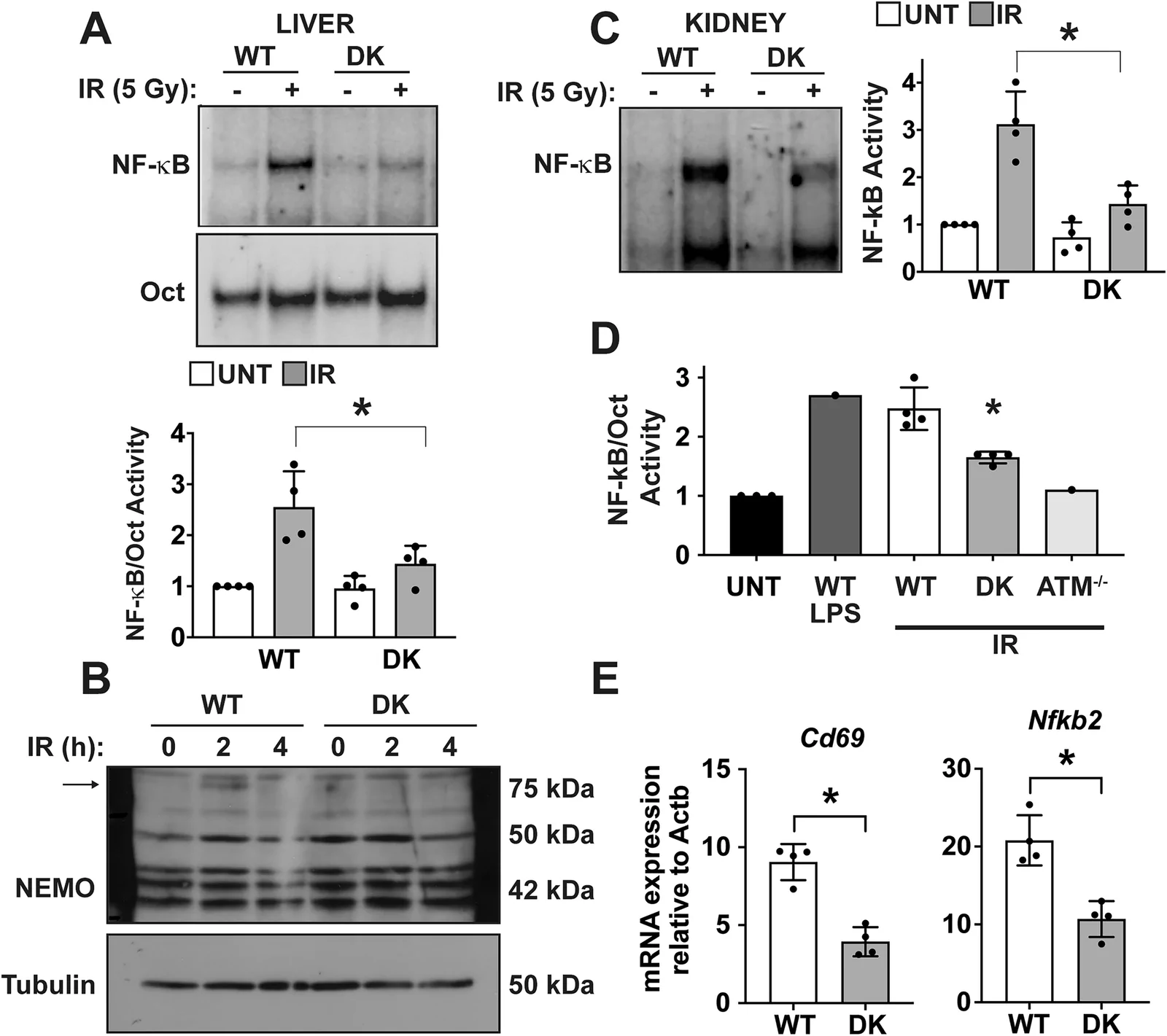
IR-induced NF-κB signaling is defective in NEMO^DK^ mice. (**A**) WT and NEMO^DK^ mice were exposed to 5 Gy whole body IR. Protein extracts isolated from the livers of WT or NEMO^DK^ mice were analyzed for NF-κB activation by EMSA. Oct-1 is used as a loading control. Data is represented as NF-κB activation over Oct-1 control relative to untreated WT samples with mean and SD (*n* = 4/genotype, *P* = 0.04). (**B**) Protein extracts isolated from the livers of WT or NEMO^DK^ mice treated with IR for the indicated times (h) were immunoblotted for NEMO and tubulin (*n* = 1). Arrow points to modified NEMO consistent with sumoylation. (**C**) WT and NEMO^DK^ mice were exposed to 5 Gy whole body IR. Protein extracts isolated from the kidneys of WT or NEMO^DK^ mice were analyzed for NF-κB activation by EMSA and quantified. Kidney samples did not have Oct-1 binding. Data is represented as NF-κB relative to untreated WT samples with mean and SD (*n* = 4/genotype, *P* = 0.01). (**D**) Pre-B cells isolated from bone marrow of WT, NEMO^DK^ (*P* = 0.02), and ATM^−/−^ mice were treated with 5 Gy IR and analyzed for NF-κB activation by EMSA. WT cells were also treated with LPS (10 µg/mL) for 1 h as a positive control. Data is represented as fold change compared to untreated control with mean and SD (*n* = 4), except for ATM^−/−^ (*n* = 1). (**E**) Pre-B cell cultures derived from WT and NEMO^DK^ mouse bone marrow were irradiated with 5 Gy and analyzed 6 h later by qPCR. Data is represented as expression relative fold change and SD of *CD69* (*P* = 0.0006) and *nfκB2* (*P* = 0.003) (*n* = 4/genotype). Data information: statistical analysis was performed using unpaired two-tailed Welch’s *t* test to compare WT and NEMO^DK^ samples. “*” indicates statistically significant. Source data are available online for this figure.

Upon whole body ionizing radiation (IR) exposures, NEMO^DK^ liver showed a significant reduction in NF-κB activation as measured by electrophoretic mobility shift assay (EMSA) compared to WT littermates (Fig. 1A). Notably, an IR-induced modified NEMO band corresponding to its sumoylation was detectable in WT, but not NEMO^DK^ liver tissue (Fig. 1B). Similarly, whole body IR showed significantly reduced NF-κB activation in the kidneys of NEMO^DK^ mice compared to WT controls (Fig.1C).

As physiological DNA breaks induced by V(D)J recombination have been shown to activate an ATM-NEMO-NF-κB dependent transcriptional program, which is also initiated by DNA-damaging agents such as IR, we examined IR-induced NF-κB activation in developing pre-B cells from the bone marrow of NEMO^DK^ mice (Bredemeyer et al, 2008; Innes et al, 2013). Upon irradiation, NEMO^DK^ pre-B cells exhibited a significant reduction in IR-induced NF-κB activation (Fig. 1D), as well as reduced transcription of its target genes *CD69* and *NfκB2* (Fig. 1E). These results collectively indicated that the NEMO^DK^ mouse model shows DNA damage-selective defects in NF-κB activation in multiple tissue/cell types.

### NEMO^DK^ mice show normal B and T cell development

**Figure EV1:**
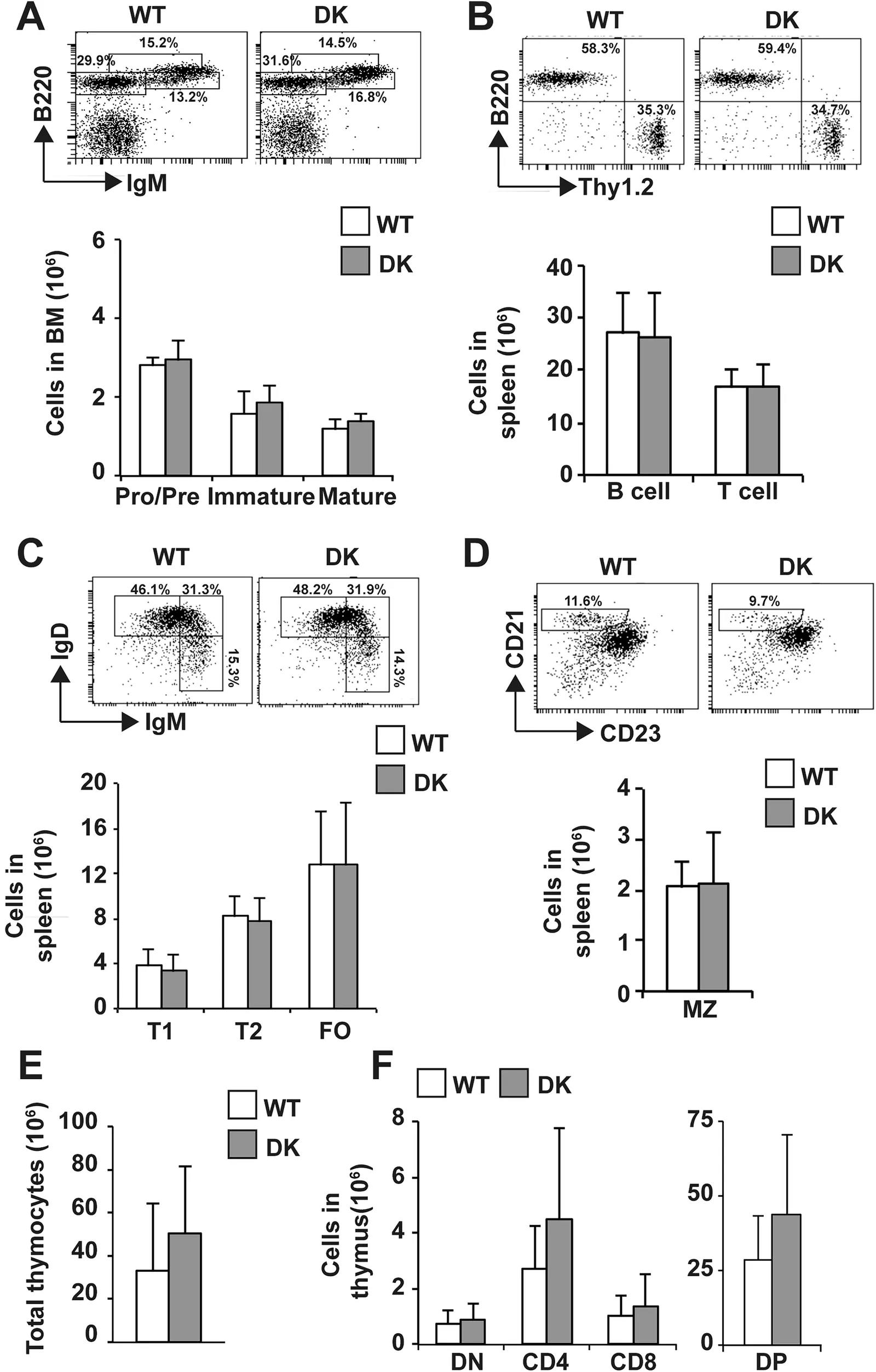
NEMO^DK^ mice display normal lymphocyte development and maturation*.* (**A**) Bone marrow cells from WT and NEMO^DK^ mice were stained with anti-B220 and anti-IgM. Percentages shown by each box indicate cells in the gated lymphoid population. Flow cytometry plots of pro-/pre- (B220^lo^IgM^−^), immature (B220^lo^IgM^+^), and recirculating mature (B220^hi^IgM^+^) B cells shown are representative of five mice per genotype and displayed graphically with mean and SD. (**B**) Flow cytometry plots of splenic B (B220^+^) and T cells (Thy1.2^+^) is displayed graphically with mean and SD (*n* = 5). (**C**) Splenocytes were stained with anti-B220, anti-IgM, and anti-IgD. For cells gated on B220^+^, flow plots of T1 (IgM^hi^IgD^−^), T2 (IgM^hi^IgD^+^), and FO (IgM^lo^IgD^+^) B cells are shown and displayed graphically with mean and SD (*n* = 5). (**D**) Splenocytes were stained with anti-B220, anti-CD21, and anti-CD23. For cells gated on B220^+^, flow plots of MZ B cells (CD21^hi^CD23^lo^) are shown and graphically displayed with mean and SD (*n* = 5). (**E**) Thymocytes from WT and NEMO^DK^ mice were stained with anti-CD4 and anti-CD8 antibodies. Data is represented as bar graphs of the total number of cells with mean and SD (*n* = 5/genotype). (**F**) Cells from (**E**) represented bar graphs of the total number of double negative (DN), CD4^+^, CD8^+^, and double positive (DP) T cells with mean and SD (*n* = 5/genotype). Data information: statistical analysis was performed using unpaired two-tailed Welch’s *t* test to compare WT and NEMO^DK^ samples. *P* values are indicated: *P*≤ 0.05.

NF-κB was previously shown to be activated by V(D)J recombination-associated DNA breaks in a manner dependent on ATM and NEMO, which suggested the possibility that attenuation of such signaling might induce alterations in B and T cell development (Bredemeyer et al, 2008). Thus, we conducted flow cytometry analyses on bone marrow cells to determine whether there was any defect in B and T cell development in NEMO^DK^ mice. We found equivalent numbers of pro-/ pre-, immature, and recirculating mature B cell populations in the mutant bone marrow (Fig. EV1A). In the spleen, overall numbers of B and T cells, as well as T1, T2, follicular (FO), and marginal zone (MZ) B cell populations, were also equivalent between WT and NEMO^DK^ mice (Fig. EV1B–D). In the thymus, the overall number of thymocytes, double negative (DN), CD4^+^, CD8^+^, and double positive (DP) cells was also comparable between WT and NEMO^DK^ mice (Fig. EV1E,F). These results suggested that there were no apparent defects in B and T cell development and maturation.

### NEMO^DK^ B cells fail to mount optimal germinal center and antibody response in vivo

**Figure EV2:**
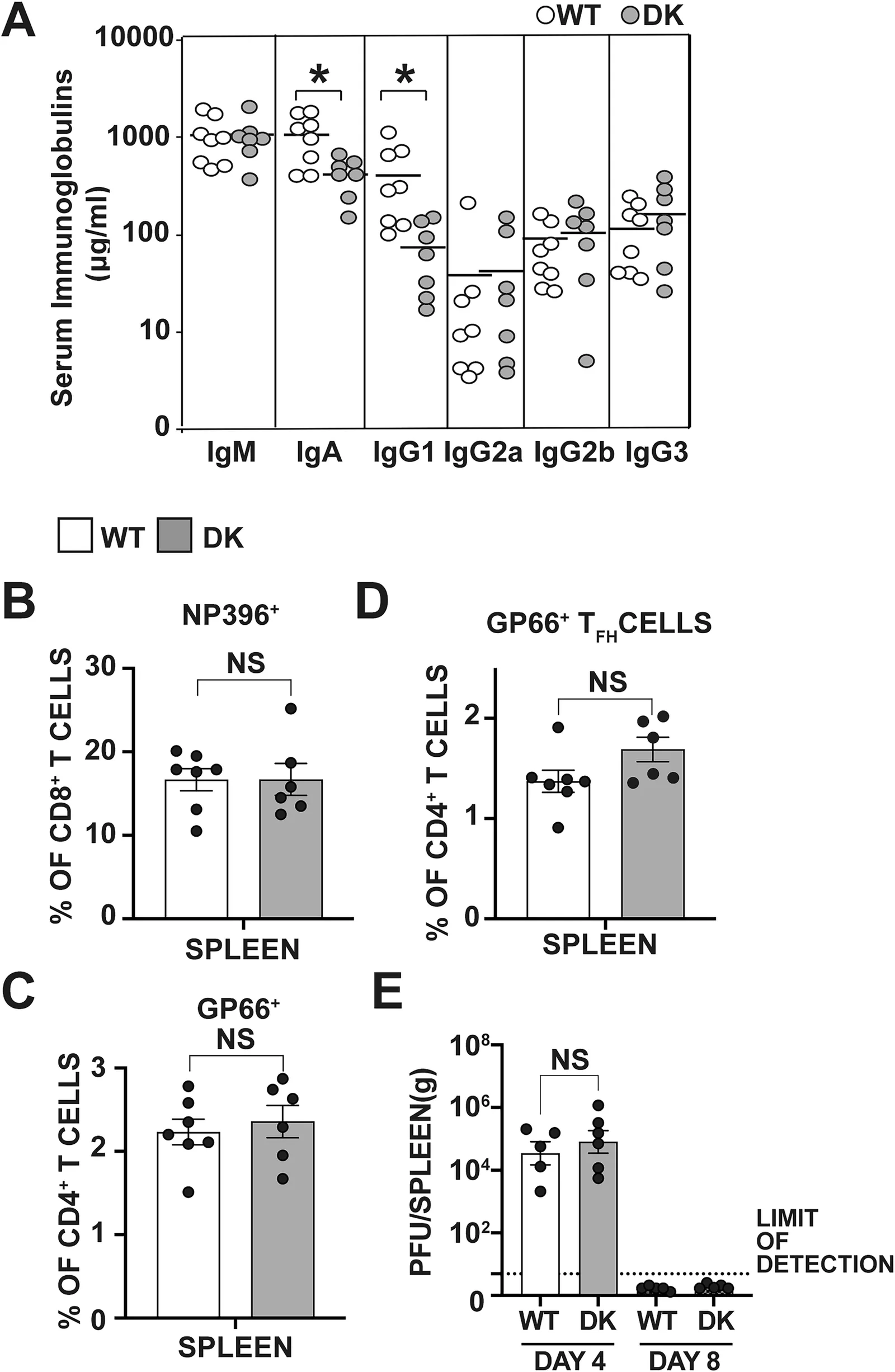
NEMO^DK^ mice exhibit decreased basal antibody levels but normal T cell responses to lymphocytic choriomeningitis virus. (**A**) Basal amounts of indicated serum Ig subtypes were determined by ELISA. Data was obtained from WT (*n* = 8) and NEMO^DK^ (*n* = 7) mice. Cohorts of WT and NEMO^DK^ mice were infected with LCMV-Armstrong. On the 8th day after infection, T cell responses were assessed in the spleen. Single cell suspensions of splenocytes were stained with anti-CD4, anti-CD8, D^b^/NP396 tetramers, I-A^b^/GP66 tetramers, anti-ICOS and anti-PD-1 antibodies. (**B**) Graphical representation of the percentages of NP396-specific cells among CD8^+^ T cells with mean and SD, *P* = 0.9853 (WT *n* = 7, DK *n* = 6). (**C**) Graphical representation of the percentages of GP66-specific cells among CD4^+^ T cells with mean and SD, *P* = 0.6136 (WT *n* = 7, DK *n* = 6). (**D**) Graphical representation of the percentages T_FH_ cells among GP66-specific CD4^+^ T_FH_ cells with mean and SD, *P* = 0.08 (WT *n* = 7, DK *n* = 6). (**E**) Graphical representation of LCMV titers in spleen at day 4 and 8 after infection with mean and SD (WT *n* = 7, DK *n* = 6). Data information: statistical analysis was performed using unpaired Student’s *t* test to compare WT and NEMO^DK^ samples. *P* values are indicated: ns, not significant; “*”, significant with *P*≤ 0.05.

**Figure 2:**
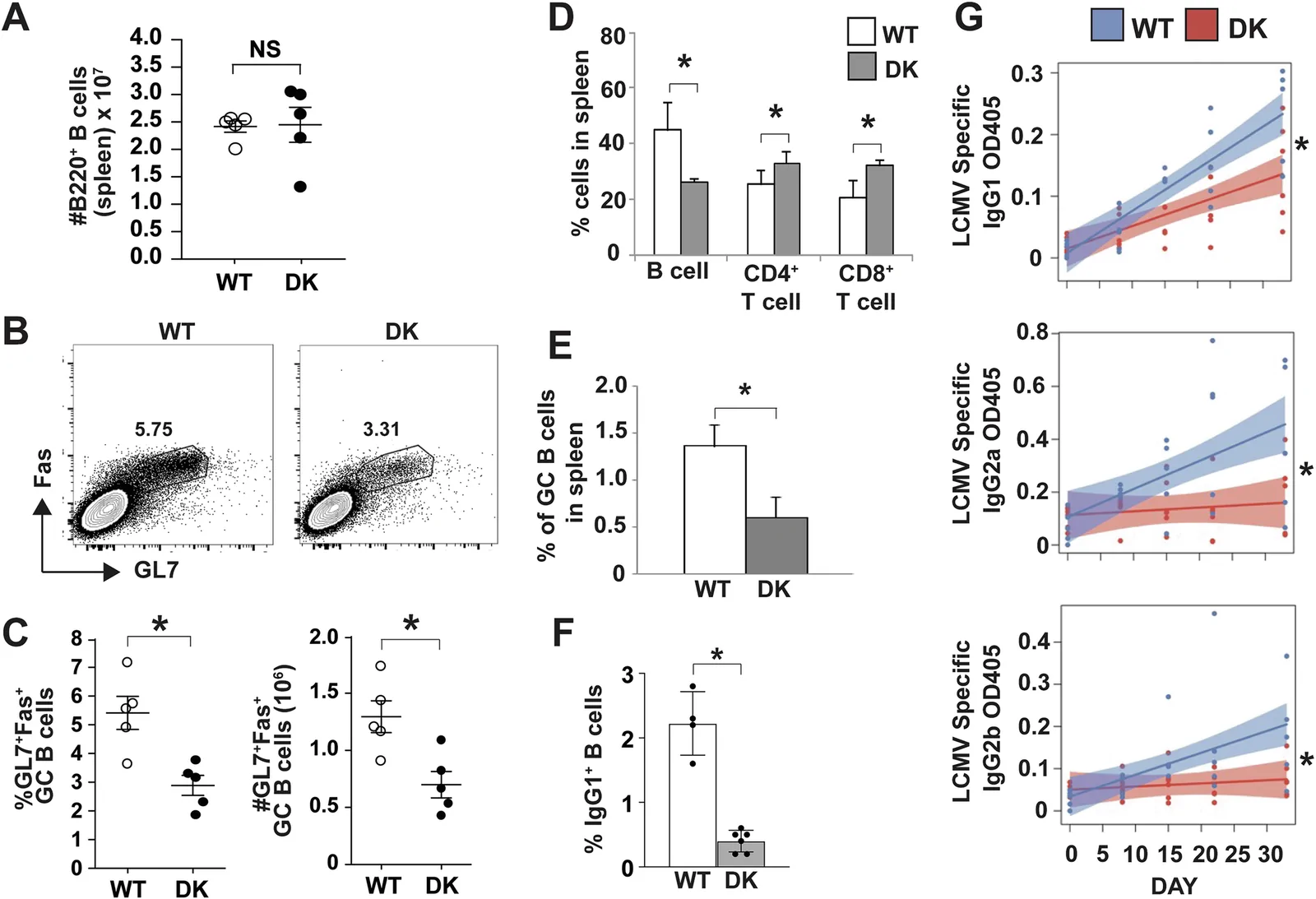
GC and class-switched antibody response against LCMV infection are defective in NEMO^DK^ mice. Splenocytes from LCMV-infected WT and NEMO^DK^ mice 8 days post-infection were analyzed by flow cytometry (**A**–**C**). Mixed bone marrow chimeras generated from WT and NEMO^DK^ mice in lethally irradiated B cell deficient (µMT) mice were infected with LCMV and analyzed post infection (*n* = 5 WT chimeras, *n* = 6 NEMO^DK^ chimeras) (**D**–**G**). Cells were analyzed 33 days post infection by FACS (**D**–**F**). (**A**) Quantification of B220^+^ B cells represented as mean and SEM (*P* = 0.92). (**B**) FACS plots of GC B cells (GL7^+^ Fas^+^) gated on B220^+^. (**C**) Percentages (*P* = 0.006) and absolute numbers (*P* = 0.01) of GC B cells gated in the B cell population in (**B**) (*n* = 5). (**D**) Percentages of splenic B (*P* = 0.01), CD4^+^(*p* = 0.05), and CD8^+^ (*P* = 0.01). Cells represented as mean and SEM (*n* = 5 WT chimeras, *n* = 6 NEMO^DK^ chimeras). (**E**) Percentages of GC B cells (*P* = 0.0003) represented as mean and SD (*n* = 5 WT chimeras, *n* = 6 NEMO^DK^ chimeras). (**F**) Percentages of IgG1^+^ B cells (*P* = 0.0034) represented as mean and SD (*n* = 5 WT chimeras, *n* = 6 NEMO^DK^ chimeras). (**G**) Blood sera were analyzed for virus-specific IgG1 (top, *P* < 0.001), IgG2a (middle, *P* = 0.002), and IgG2b (bottom, *P* = 0.006) by ELISA (OD_405_) at the indicated days post-infection, represented by a linear regression model. Data information: statistical analysis was performed using Mann–Whitney (**A**–**C**), unpaired two-tailed Welch’s *t* test to compare WT and NEMO^DK^ samples (**D**–**F**), and Ordinary one-way ANOVA with multiple comparisons to test for linear trend (**G**). *P* values are indicated: ns, not significant; “*” indicates statistically significant. Source data are available online for this figure.

Despite apparently normal development and maturation of B cells, we found that NEMO^DK^ mice had lower amounts of basal serum IgA and IgG1, suggesting compromised antibody production in NEMO^DK^ mice (Fig. EV2A). This could be due to defects in B cells, T follicular helper (T_FH_), or both. To further characterize the immune functions of the mutant mice, we next infected WT and NEMO^DK^ mice with lymphocytic choriomeningitis virus (LCMV)-Armstrong strain and monitored anti-viral T and B cell responses. At day 8 post-infection, which correlates to the peak of T cell response, the number and percentage of virus-specific CD8^+^ and CD4^+^ cells were similar between WT and NEMO^DK^ mice (Fig. EV2B,C). T_FH_ cell response was also similar in these mice (Fig. EV2D). Virus titers were similarly high at day 4 post-infection and became undetectable at day 8 post-infection in the spleen (Fig. EV2E). These results further indicated that T cell functions were normal in NEMO^DK^ mice. However, despite having equivalent numbers of total splenic B cells, NEMO^DK^ mice showed significantly reduced germinal center (GC) B cells compared to WT mice when measured by flow cytometry (Fig. 2A–C).

The lack of defects in T_FH_ CD4^+^ T cells (Fig. EV2D) suggested that the reduced GC response seen in NEMO^DK^ mice is likely B cell intrinsic. To test this, we created mixed bone marrow chimeras from WT and NEMO^DK^ mice in lethally irradiated B cell-deficient (${{{\rm{\mu }}}}$MT) mice and infected them with LCMV-Armstrong strain. In these chimeras, the bone marrow of either WT or NEMO^DK^ mice was mixed at a 1:4 ratio with bone marrow from host ${{{\rm{\mu }}}}$MT mice. This enabled specific evaluation of B cell-intrinsic defects as B cells are derived from either WT or NEMO^DK^ mice while all other cell types remain normal. We found the infected NEMO^DK^ B cell chimeras had lower percentages of B cells in the spleen compared to WT chimeras, suggesting a defect in B cell response. Percentages of CD4^+^ and CD8^+^ T cells were slightly greater in NEMO^DK^ chimeras, which is likely a reflection of the lower percentages of B cells in the spleen (Fig. 2D). In line with this finding, we also found decreased percentages of GC and IgG1^+^ B cells in NEMO^DK^ chimeras compared to WT (Fig. 2E,F). Moreover, NEMO^DK^ chimeras failed to mount optimal virus-specific class-switched antibody responses (Fig. 2G). These results strongly suggest a B cell-intrinsic role for NEMO K270/K302 in regulating GC and antibody responses in vivo.

### CD40-induced homotypic aggregation (HA) is reduced in NEMO^DK^ B cells

**Figure 3:**
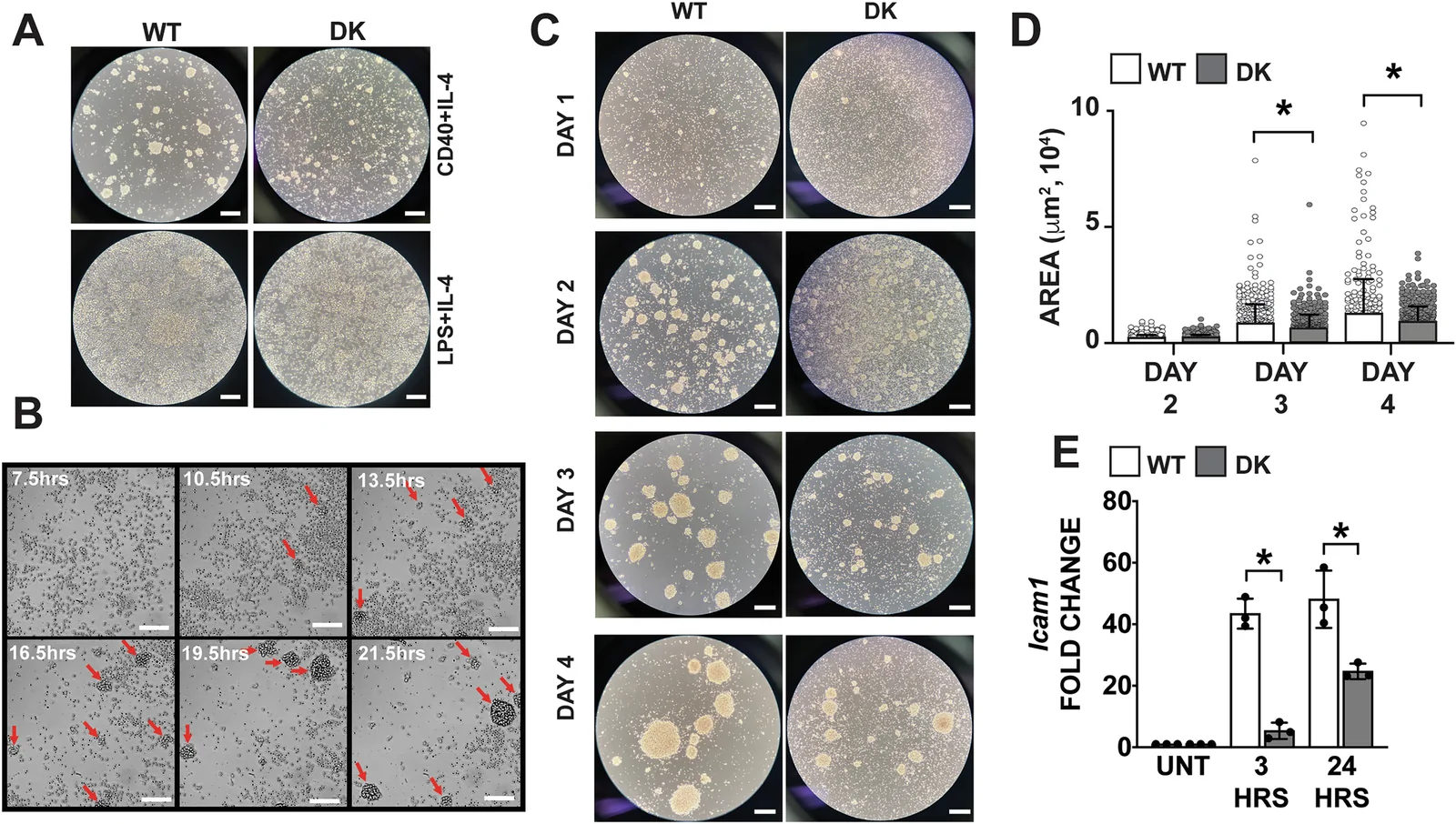
CD40-driven homotypic aggregation is defective in NEMO^DK^ B cells. (**A**) Representative images of WT and NEMO^DK^ B cells stimulated with either CD40 + IL-4 or LPS + IL-4 taken 2 days post stimulation. Scale bar on bottom right represents 100 µm. (**B**) WT splenic B cells were stimulated with CD40 + IL-4 and imaged from 6–24 h post stimulation. Representative images starting 7.5 h post stimulation until 21.5 h, at 3 hr intervals with red arrows indicating aggregates, scale bar on bottom right represents 100 µm (*n* = 1). (**C**) Representative images of WT and NEMO^DK^ B cells stimulated with CD40 + IL-4 over the course of 4 days, scale bar on bottom right represents 100 µm. (**D**) Quantification of the size of homotypic aggregates from CD40 + IL-4 stimulated WT and NEMO^DK^ B cells from days 2–4. Data is graphically represented as bar graphs indicating the mean area and SD, with each dot representing an aggregate (*n* = 3, *P* = 9.4 × 10^−6^). (**E**) Quantification of *Icam1* mRNA measured by qPCR of CD40 + IL-4 stimulated WT and NEMO^DK^ B cells 3 (*P* = 0.001) and 24 h (*P* = 0.01) post stimulation. Data is represented as fold change compared to WT untreated control with mean and SD (*n* = 3). Data information: statistical analysis was performed using unpaired two-tailed Welch’s *t* test to compare WT and NEMO^DK^ samples. *P* values are indicated: “*” indicates statistically significant. Source data are available online for this figure.

Activated by T_FH_ cells, CD40 engagement provides necessary signals to initiate and sustain the GC reaction, driving B cells to proliferate, survive, and undergo CSR (Elgueta et al, 2009). Due to the defects in GC response and cell-intrinsic and class-switched antibody production against LCMV infection in NEMO^DK^ mice and chimeras, we wanted to examine if agonistic anti-CD40 antibody-induced cellular functions were also defective in NEMO^DK^ B cells. Stimulation of CD40 on B cells in vitro also induces homotypic aggregation (HA), where B cells form large, spherical, and highly stable clusters, a phenotype that was further enhanced by IL-4 costimulation (Klaus et al, 1994). We stimulated resting naive B cells, isolated from spleens of WT or NEMO^DK^ mice, with agonistic CD40 antibody in the presence of IL-4 (from here on referred to as “CD40 + IL-4”). WT B cells formed large aggregates compared to NEMO^DK^ cells, which were absent in samples stimulated with LPS + IL-4 (Fig. 3A), demonstrating the CD40 specificity of this cellular phenotype. To understand how HA is initiated, time-lapse imaging was conducted on CD40 + IL-4 stimulated WT B cells. This analysis revealed progressive, accretive fusion of cell clusters, in which aggregates coalesced with neighboring clusters to generate increasingly larger HA structures overtime, a process clearly evident at approximately 10 h post stimulation (Fig. 3B).

Under longer-term stimulation, WT B cells consistently formed larger aggregates than NEMO^DK^ B cells over time (Fig. 3C). Quantification of aggregate size revealed that aggregates formed by NEMO^DK^ B cells were significantly smaller than those formed by WT cells beginning at day 3 post stimulation (Fig. 3D). As CD40-induced HA is dependent on ICAM-1, which is known to be an NF-κB regulated gene product, we examined *Icam1* mRNA by qPCR (Melotti et al, 2001). We analyzed CD40 + IL-4 stimulated WT and NEMO^DK^ B cells 3 and 24 h post stimulation and found *Icam1* mRNA is significantly decreased at both time points (Fig. 3E). This indicated that *Icam1* expression is lower in NEMO^DK^ B cells before the onset of HA. Together, these findings demonstrate that NEMO K270/K302 is required for efficient CD40-driven HA formation in B cells, and that the reduced aggregate size observed in NEMO^DK^ B cells reflects potential defects in NF-κB signaling and *Icam1* expression to promote cluster growth.

### CD40-induced proliferation and antibody class switching are defective in NEMO^DK^ B cells

**Figure 4:**
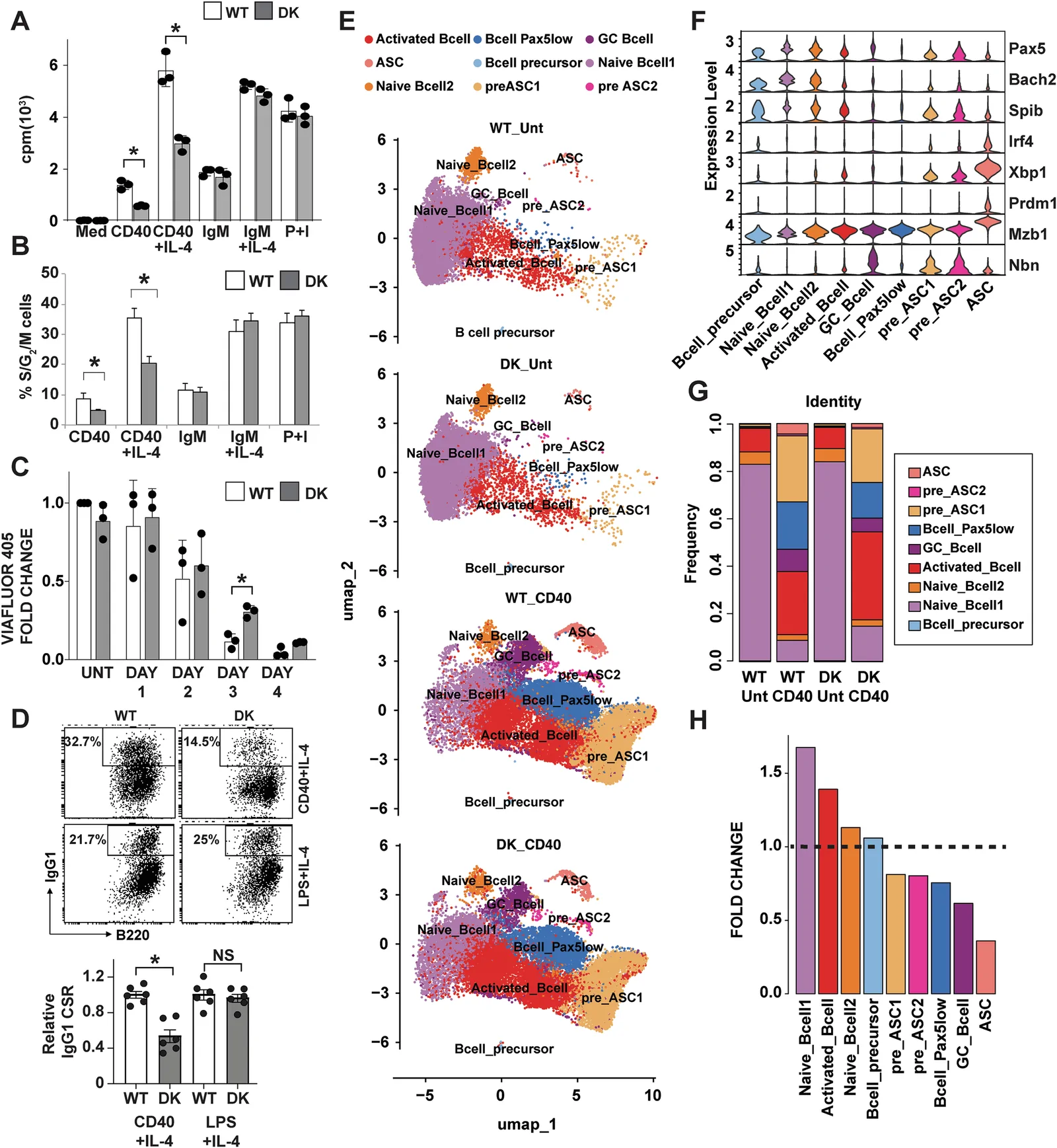
NEMO^DK^ B cells exhibit CD40-specific defects in proliferation, IgG1 CSR, and differentiation ex vivo. (**A**) Splenic WT and NEMO^DK^ B cells were treated with CD40 (2 µg/mL, *P* = 0.01) with and without IL-4 (10 ng/mL, *P* = 0.007), anti-IgM (1 µg/mL) with and without IL-4 (10 ng/mL), or P + I (PDBu at 10 ng/mL plus ionomycin at 1 µg/mL) for 48 h. Proliferation was measured by ^3^H-thymidine incorporation. The graph shows mean and SD (*n* = 3). (**B**) Splenic WT and NEMO^DK^ B cells were treated as in (**A**) for 40 h, stained with propidium iodide, and analyzed for cell cycle profiles by flow cytometry. Graphical representation of the percentage of S/G_2_/M cells expressed as mean and SD (*n* = 8, CD40 and CD40 + IL-4 conditions *P* = 1.38 × 10^−7^). (**C**) Quantification of the MFI of cells stained with Viafluor 405 represented as relative fold change compared to WT untreated control with mean and SD (*n* = 3, day 3 *P* = 0.001). The lower Viafluor 405 intensity represents more proliferation. (**D**) Splenic B cells from WT and NEMO^DK^ mice were stimulated for 4 days with CD40 + IL-4 and LPS + IL-4 and analyzed for in vitro class switch recombination by detecting surface IgG1 expression by FACS. Data show representative 3 independent experiments; each experiment includes 2 WT and 2 NEMO^DK^ mice. Upper: FACS plots. Lower: The percentage of IgG1^+^ class-switched cells displayed graphically with mean and SD (*n* = 6/genotype/condition, CD40 + IL-4 *P* = 0.003). (**E**) Splenic B cells from WT and NEMO^DK^ mice were stimulated for 3 days with CD40 + IL-4 and analyzed by single cell RNA sequencing. UMAP (Uniform Manifold Approximation and Projection) projection of B cells across different conditions are displayed. (**F**) Violin plot of expression level (log scale) of representative B cell gene signatures across different B cell subsets from (**E**, *n* = 2). (**G**) Frequencies of different B cell subsets across conditions from (**E**). (**H**) Fold-change difference of NEMO^DK^ samples compared to WT samples in frequency of B cell subsets (**G**). Data information: statistical analysis was performed using unpaired two-tailed Welch’s *t* test to compare WT and NEMO^DK^ samples. *P* values are indicated: ns, not significant; “*”, significant with a *P*≤ 0.05 Source data are available online for this figure.

**Figure EV3:**
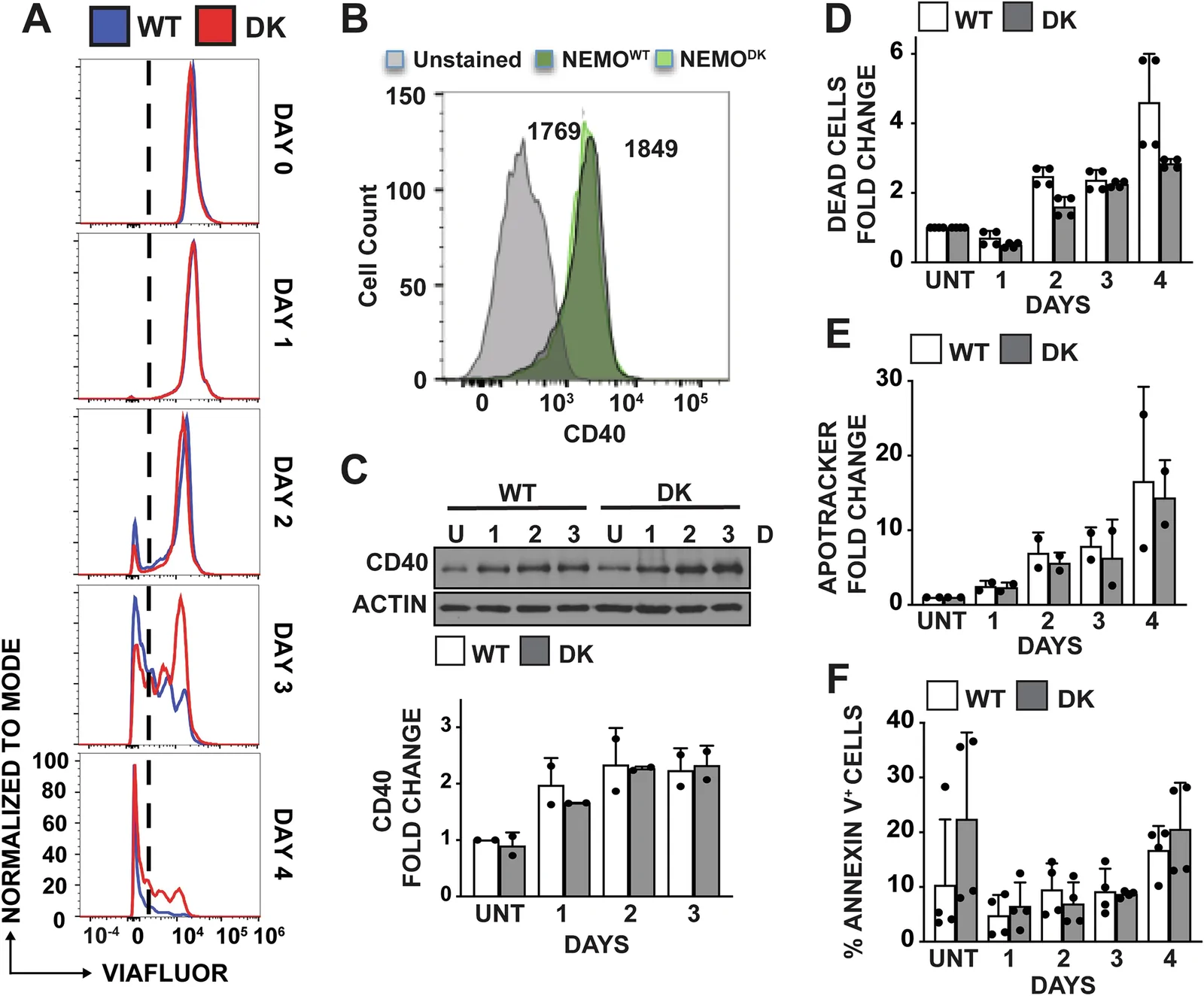
CD40-specific defects in NEMO^DK^ B cells are not due to different CD40 expression or increased cell death. (**A**) WT and NEMO^DK^ B cells were stimulated with CD40 + IL-4 and stained with Viafluor 405 to track proliferation over 4 days and analyzed by FACS. FACS plots are represented as histograms, the dotted line denotes the population of cells that have divided (left) and not divided (right). (**B**) Splenic B cells were stained for CD40 and analyzed by flow cytometry. Representative MFI are displayed (*n* = 5). (**C**) Lysates from WT and NEMO^DK^ B cells were stimulated with CD40 + IL-4 over the course of 3 days and analyzed by immunoblot for CD40 protein and actin as loading control. Data is represented graphically as CD40 expression normalized to actin with relative fold change compared to WT untreated control with mean and SD (*n* = 2). (**D**) Quantification of dead cells measured by Live-or-Dye Nucfix^TM^ Red viability stain of WT and NEMO^DK^ B cells stimulated with CD40 + IL-4. Data represented as relative fold change compared to WT untreated control with mean and SD (*n* = 4). (**E**) Cells treated as described in (**D**) were stained with Apotracker Green and quantified with data represented as in (**D**) (*n* = 2). (**F**) WT and NEMO^DK^ B cells were treated as described in (**D**), stained with Annexin V and quantified. Data is represented as the mean percentage of annexin 5^+^ cells with SD (*n* = 4). Data information: statistical analysis was performed using unpaired two-tailed Welch’s *t* test to compare WT and NEMO^DK^ samples.

We further investigated proliferation of WT and NEMO^DK^ B cells using CD40 ( + /- IL-4) stimulation and compared this to B cells treated with agonistic anti-IgM antibody (+/- IL-4) to stimulate the B cell receptor (BCR) or phorbol-12,13-dibutyrate (PDBu) plus ionomycin (P + I) to mimic combined signaling from the BCR and costimulatory molecules. NEMO^DK^ B cells displayed defective CD40-, but not IgM-, or P + I-, induced proliferation 48 h post treatment as measured by ^3^H-thymidine incorporation into replicating DNA (Fig. 4A). Cell cycle analysis corroborated the proliferation data by showing reduced levels of NEMO^DK^ B cells in the S and G2-M phases after stimulation with CD40 (+ /− IL-4) for 24 h (Fig. 4B). To examine proliferation over a longer period, ViaFluor 405, a membrane permeable dye similar to carboxyfluorescein succinimidyl ester (CFSE) that decreases in intensity with each round of cell division, was used (Fig. EV3A). While both WT and NEMO^DK^ B cells initiated proliferation approximately 2 days after CD40 + IL-4 stimulation, mutant B cells proliferated significantly less than WT cells at day 3. (Figs. 4C and EV3A).

To determine whether the CD40 specificity of the proliferation defect was due to decreased CD40 expression in NEMO^DK^ B cells, CD40 was measured by flow cytometry in WT and NEMO^DK^ cells at baseline and found similar levels of surface CD40 (Fig. EV3B). Using immunoblot analysis, similar CD40 protein levels were also observed in CD40 + IL-4 stimulated WT and NEMO^DK^ B cells (Fig. EV3C).

Next, to determine whether the CD40-induced proliferation defect in NEMO^DK^ B cells was attributable to increased cell death, we measured cell viability and apoptosis. Using Live-or Dye NucFix^TM^ Red, a nuclear dye that stains cells with permeable membranes associated with cell death, we found CD40 + IL-4 stimulated WT and NEMO^DK^ B cells showed comparable percentages of dead cells (Fig. EV3D). Similarly, apoptosis was also comparable between WT and NEMO^DK^ B cells, which was measured by staining externalized phosphatidylserine phospholipids by Annexin 5 and Apotracker^TM^ Green (Fig. EV3E,F). Altogether, these results show the CD40-induced proliferation defects were unlikely due to decreased expression of CD40 or increased cell death in NEMO^DK^ B cells. Thus, NEMO K270/K302 appears to impact proliferation independent of cell survival.

B cells aggregate with T-helper cells and other B cells in the GC or lymphoid tissues to receive the necessary costimulatory signals and cytokines required for activation and CSR (Zaretsky et al, 2017; Koopman et al, 1994; Bjorck and Paulie, 1993). Because HA formation and cell proliferation are critical for CSR, expression of class-switched antibody was examined in WT and NEMO^DK^ B cells stimulated with CD40 + IL-4 or LPS + IL-4 as a specificity control (Klaus et al, 1994; Thakur et al, 2011; Vijayashankar and Vaidya, 2021). As IL-4 specifically induces IgG1 class switching, FACS analysis of cell surface IgG1^+^ B cells was conducted and we found that LPS + IL-4 stimulation produced comparable frequencies of IgG1^+^ B cells in both genotypes (Fig. 4D) (Snapper et al, 1988). In contrast, CD40 + IL-4 stimulated NEMO^DK^ B cells generated significantly fewer IgG1^+^ B cells than WT counterparts (Fig. 4D). Thus, NEMO^DK^ B cells have cell-intrinsic defects in undergoing CSR specifically under CD40-stimulated conditions.

While CD40 signaling is required for humoral immunity, the strength and duration impact the fate of B cells. In the absence of CD40 there is no GC response, while excess CD40 stimulation during a primary immune response can also result in inhibition of GC formation by promoting the differentiation of naïve B cells into plasmablasts localized outside of the B cell follicles (Elgueta et al, 2009). Another study also showed the strength of CD40 signaling, which is translated into NF-κB activation and subsequent BATF upregulation, promotes memory B cell differentiation from GC B cells (Koike et al, 2019). Given the comparable CD40 expression between WT and NEMO^DK^ B cells, and equivalent amounts of anti-CD40 are administered to each sample, we wanted to understand how the difference in NF-κB signaling strength contributed to B cell fate. Here, we conducted single-cell RNA-sequencing (scRNA-seq) on CD40 + IL-4 stimulated WT and NEMO^DK^ B cells 3 days post stimulation. Analysis was conducted at day 3 to capture the transitional phase where CSR has been initiated and proliferative activity is approaching its peak (Fig. EV3A), allowing us to capture the most cellular diversity. ScRNA-seq analysis identified an expansion of B cell states, with a shift from predominantly naive B cells to more activated, GC, and antibody secreting B cell states (Fig. 4E). Notably, we identified antibody secreting cell (ASC), and potentially pre-ASC, states based on the recently identified gene signatures of B cell differentiation using scRNA-seq, including the downregulation of *Pax5, Bach2, Spib*, and *Nbn*, coupled with high expression of *Xbp1, Prdm1, Mzb1*, and *Irf* (Fig. 4F) (Verstegen et al, 2023). ASC cells are not present in unstimulated conditions in both WT and NEMO^DK^ samples (Fig. 4G). When we compared  ratios of different cell populations of NEMO^DK^ to WT samples , the lowest fold-change (0.36) was observed for ASC, while the highest (1.67) was observed for naïve B cells (Fig. 4G,H). Combined with approximately half of the total cells present in NEMO^DK^ relative to WT cells due to the reduced proliferation of stimulated  NEMO^DK^ B cells (Fig. 4A–C), these scRNA-seq results suggest that NEMO^DK^ B cells fail to efficiently progress across the differentiation pathway toward ASC states following CD40 + IL-4 stimulation.

Collectively, these results revealed that NEMO K270/K302 specifically promote HA formation, proliferation, CSR, and generation of ASCs upon CD40 + IL-4 stimulation ex vivo.

### RelA (p65) is the main transcription factor driving transcriptomic defects in CD40-stimulated NEMO^DK^ B cells

To gain insight into the genome-wide transcriptional defects of CD40 signaling in NEMO^DK^ B cells, we next performed bulk RNA sequencing (RNA-seq) analysis of WT and NEMO^DK^ B cells stimulated with CD40 for 24 h. Differentially expressed transcripts with an FDR ≤ 0.05 using the DESeq2 program (see “Methods”) identified ~900 genes up- and ~1100 genes downregulated in NEMO^DK^ B cells compared to WT B cells (Appendix Fig. S2A). The gene set enrichment analysis (GSEA) showed CD40 up-regulated genes were highly enriched in WT but not NEMO^DK^ B cells, whereas the CD40 downregulated genes were enriched in NEMO^DK^ B cells (Appendix Fig. S2B). Using the database for annotation, visualization and integrated discovery (DAVID) analysis of 40 genes up- and 117 genes downregulated more than 1.5-fold (corrected *P* value < 0.05) in NEMO^DK^ B cells versus WT B cells, we found the top three clusters in downregulated genes included genes related to cell cycle, DNA replication, and DNA repair (Appendix Fig. S2C). Promoter motif analysis of downregulated genes in NEMO^DK^ B cells relative to WT B cells using hypergeometric optimization of motif enrichment (HOMER) revealed a strong enrichment of canonical NF-κB RelA/p50 dimer binding sites over NF-κB dimer cRel/p50 and noncanonical RelB/p52 at 24 h post CD40 stimulation (Appendix Fig. S2D). Moreover, cell cycle, DNA replication, and DNA damage repair genes were again associated with RelA/p50 binding sites using DAVID analysis (Appendix Fig. S2E), suggesting their possible regulation by RelA/p50 NF-κB complexes. The downregulated gene clusters of cell cycle and DNA replication genes could explain the CD40-induced proliferation defects in NEMO^DK^ B cells (Fig. 4A–C). Induction of DNA damage repair genes (Appendix Fig. S2C) suggested the potential involvement of the DNA damage response in CD40-stimulated B cells.

**Figure 5:**
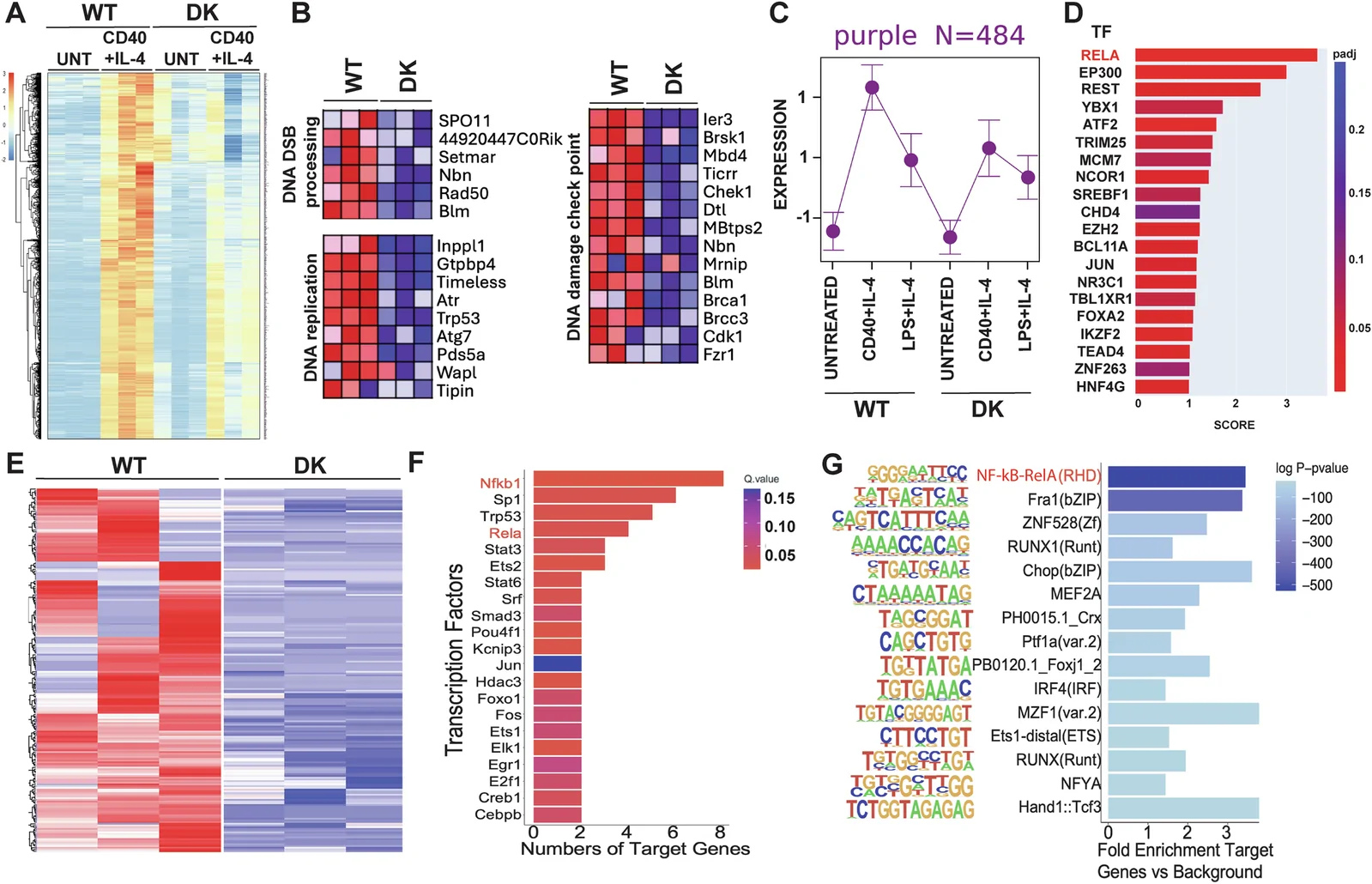
Transcriptomic and epigenetic analyses predict significant downregulation of RelA transcriptional activity in NEMO^DK^ B cells stimulated with CD40 + IL-4. Splenic B cells from WT (*n* = 3) and NEMO^DK^ (*n* = 3) mice were stimulated with anti-CD40 (2μg/mL) + IL-4 (10 ng/mL) or LPS (20 ng/mL) + IL-4 (10 ng/mL) for 48 h. Cells were collected and analyzed by RNA, CUT&Tag, and ATAC sequencing. (**A**) 7648 genes were found to be differentially expressed across the six conditions (WT untreated, WT CD40 + IL-4, WT LPS + IL-4, NEMO^DK^ untreated, NEMO^DK^ CD40 + IL-4, and NEMO^DK^ LPS + IL-4) using the Likelihood Ratio Tests in Deseq2 at 2x relative fold change and adjusted p < 0.01. Data from WT untreated, WT CD40 + IL-4, NEMO^DK^, and NEMO^DK^ untreated CD40 + IL-4 are represented as a heatmap. (**B**) Heatmap of gene signatures that showed core enrichment in CD40 + IL-4 WT samples compared to CD40 + IL-4 NEMO^DK^ from GSEA mouse molecular signatures database’s (MSigDB) DNA double strand break processing, DNA replication, and DNA damage checkpoint Gene ontology (M5) biological processes groups. (**C**) Of the 18 gene clusters from Appendix Fig. S3A, the purple cluster was chosen for its unique reduction of CD40 + IL-4 specific gene expression in NEMO^DK^ B cells (*n* = 3 per condition). (**D**) MAGIC analysis identified RelA as the top driver of genes from the purple cluster in (**C**). (**E**) Heatmaps displaying significantly downregulated peaks in CD40 + IL-4 NEMO^DK^ B cells compared to WT counterparts from CUT&Tag analysis. (**F**) ATAC-seq analysis of WT and NEMO^DK^ splenic B cells. Top transcription factors (*q* value < 0.25) of genes identified as downregulated from the RNA-seq analysis and have decreased signals at the promoter-TSS region in ATAC-seq were analyzed by TRRUST with the counts of transcription factors presented after the column. (**G**) HOMER motif analysis identified significant enrichment of NF-κB-RelA binding sites in peaks where NEMO^DK^ B cells showed significantly decreased peak signals in ATAC-seq analysis.

To further determine transcriptional defects of NEMO^DK^ B cells prior to the peak of CSR reaction, which occur 3–4 days post stimulation, we next performed bulk RNA-seq analysis in WT and NEMO^DK^ B cells treated with CD40 + IL-4 for two days. In parallel, cells were also stimulated with LPS + IL-4 to evaluate CD40 specificity, as CSR defects were only observed under CD40 + IL-4 conditions in NEMO^DK^ B cells (Fig. 4D). A large collection of induced genes was expressed at lower levels in the CD40 + IL-4 NEMO^DK^ vs WT samples, while untreated samples from both groups had similarly low levels of expression of such genes (Fig. 5A). The GSEA analysis on this dataset also revealed genes associated with DNA double strand break (DSB) processing, DNA damage checkpoint, and DNA replication were expressed at lower levels in CD40 + IL-4 stimulated NEMO^DK^ B cells relative to WT B cells (Fig. 5B). Further analysis of the RNA-seq data revealed 7648 genes to be differentially expressed across the three different conditions of WT and NEMO^DK^ B cells (unstimulated, CD40 + IL-4, and LPS + IL-4) with *P* ≤ 0.01. These genes were then subjected to Leiden clustering, which led to the generation of 18 gene clusters with unique expression patterns amongst the different conditions (Appendix Fig. S3A).

From these 18 clusters, we focused on the purple cluster (Fig. 5C) because CD40 + IL-4 induced genes in WT B cells had higher expression levels than NEMO^DK^ counterparts, while LPS + IL-4 stimulated WT and NEMO^DK^ B cells had similar levels of induced expression compared to unstimulated WT and NEMO^DK^ B cells. This allowed us to focus on genes whose expression was specifically diminished in NEMO^DK^ B cells when stimulated with CD40 + IL-4. We next applied the mining algorithm for genetic controllers (MAGIC), which utilizes ENCODE ChIP-seq data to identify statistically enriched transcription factors and cofactors from gene sets (Roopra, 2020). Consistent with the earlier finding with CD40 stimulation alone (Appendix Fig. S2D), the MAGIC analysis identified RelA as the top transcription factor driving the genes found within the purple cluster (Fig. 5D). In contrast, the orange cluster showed LPS + IL-4 induced genes, where WT had greater expression compared to NEMO^DK^, while CD40 + IL-4 and untreated samples showed similar levels of low expression (Appendix Fig. S3A). MAGIC analysis did not identify RelA among the transcription factors predicted to regulate genes within the orange cluster, which consist of LPS-specific genes defective in NEMO^DK^ B cells (Appendix Fig. S3B). Moreover, MAGIC analysis of genes in the aqua cluster, comprising of genes that are defective in NEMO^DK^ B cells under both CD40 and LPS stimulation, did not identify any NF-κB–related transcription factors (Appendix Fig. S3C). Overall, these results highlight RelA as a principal driver of CD40-specific, but not LPS-specific, gene expression, which is selectively defective in NEMO^DK^ B cells.

To further investigate the role of RelA in NEMO^DK^ B cells, we performed CUT&Tag sequencing on untreated and CD40 + IL-4 treated splenic B cells from WT and NEMO^DK^ mice. We used an antibody against RelA, along with antibodies targeting the histone modifications H3K4me3 and H3K27ac, well-established markers of transcriptional activation, and H3K27me3, a marker generally associated with transcriptional repression (Barski et al, 2007; Rada-Iglesias et al, 2011; Santos-Rosa et al, 2002). These modifications were analyzed to characterize promoter and enhancer chromatin landscapes. We first aligned the quality-controlled reads to the mouse genome and quantified signal intensity as reads per million mapped reads (RPM) from the transcription start site (TSS) to the transcription end site (TES). We observed an overall reduction in RelA antibody binding at the TSS region in CD40 + IL-4 stimulated WT B cells compared to NEMO^DK^ B cells (Appendix Fig. S4A), suggesting decreased RelA accessibility in NEMO^DK^ B cells under these stimulatory conditions. In contrast, signals for the histone modifications H3K4me3, H3K27ac, and H3K27me3 were statistically indistinguishable between WT and NEMO^DK^ B cells upon CD40 + IL-4 stimulation (Appendix Fig. S4B–D), indicating that global chromatin modifications were largely unaffected. We next performed peak calling to identify regions of antibody enrichment and conducted differential binding analysis using DESeq2. This analysis revealed 213 RelA peaks significantly reduced in CD40 + IL-4 stimulated NEMO^DK^ B cells compared to WT (Fig. 5E).

To also assess genome-wide chromatin accessibility, we performed Assay for Transposase-Accessible Chromatin with high-throughput sequencing (ATAC-seq) on CD40 + IL-4 stimulated B cells. For this analysis, the findings from ATAC-seq and RNA-seq on CD40 + IL-4 stimulated samples were used. Peak calling and quantification identified 122 genes showing significantly reduced promoter/TSS accessibility in NEMO^DK^ B cells, which also exhibited decreased mRNA expression relative to WT. To investigate the regulatory mechanisms underlying these transcriptional and chromatin accessibility changes, we queried the 122 genes with reduced promoter accessibility and expression in NEMO^DK^ B cells against the Transcriptional Regulatory Relationships Unraveled by Sentence-based Text mining (TRRUST) database (Han et al, 2015). This analysis identified NFκB1 and RelA among the top transcription factors predicted to regulate these genes, with enrichment observed in CD40 + IL-4 stimulated WT samples compared to NEMO^DK^ counterparts (Fig. 5F). We next performed motif enrichment analysis using the HOMER Motif Discovery pipeline on the ATAC-seq dataset (Heinz et al, 2010). Focusing on the ±200 bp regions surrounding the 5,084 peaks that exhibited significantly reduced accessibility in NEMO^DK^ B cells relative to WT upon CD40 + IL-4 stimulation, we found that the top-ranked, significantly enriched motif corresponded to the RelA binding motif (Fig. 5G). HOMER also identified significant enrichment of RelA binding sites within the subset of genes that were differentially expressed in CD40 + IL-4 stimulated samples from the ATAC-seq analysis (Fig. 5G).

Taken together, these genome-wide, unbiased transcriptomic and epigenetic analyses strongly suggest that the activity of RelA, likely in partnership with p50, is markedly attenuated in CD40 and CD40 + IL-4 stimulated NEMO^DK^ B cells relative to WT cells.

### CD40 + IL-4 induced AID and NBS1 expression is reduced in NEMO^DK^ B cells

**Figure 6:**
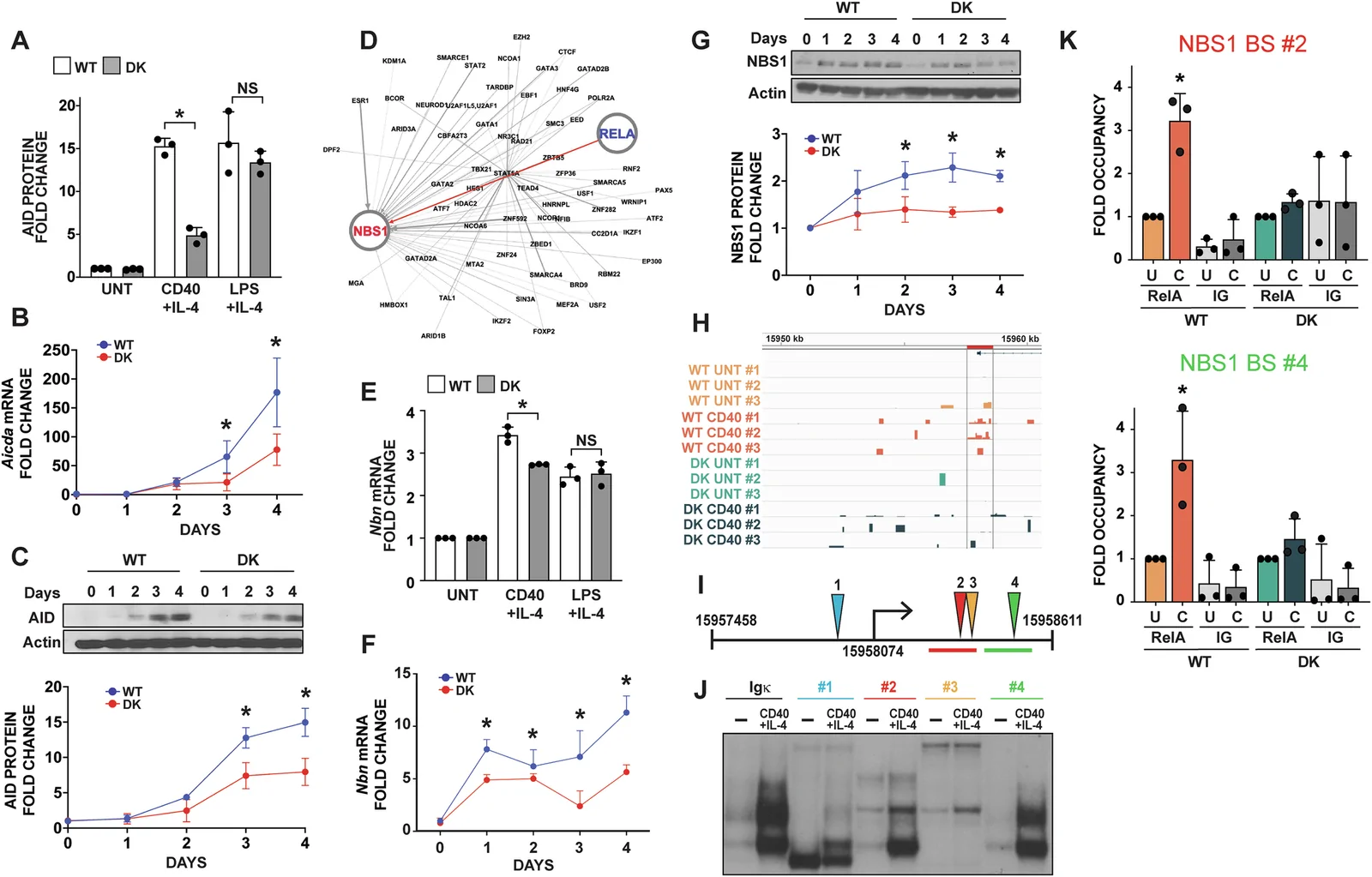
NBS1 and AID expression is decreased in CD40 + IL-4 stimulated NEMO^DK^ B cells*.* (**A**) WT and NEMO^DK^ B cells were stimulated with either CD40 + IL-4 or LPS + IL-4 for 2 days. Lysates from these cells were analyzed by immunoblot for AID expression and quantified. Data is represented as relative fold change compared to each genotype’s respective untreated control with mean and SD (*n* = 3). (**B**) WT and NEMO^DK^ B cells were stimulated with CD40 + IL-4 over the course of 4 days. Cells were analyzed for *Aicda* mRNA by qPCR. Data is represented as a time course plot with mean relative fold change compared to each genotype’s respective untreated control and SD (*n* = 3, day 3 *P* = 0.04, day 4 *P* = 0.0002). (**C**) Lysates from cells from (**B**) were analyzed by immunoblot for AID expression with actin as loading control (upper immunoblots). Quantified data is represented as in (**B**) (lower graph, day 3 *P* = 0.0184, day 4 *P* = 0.0117). (**D**) Projection of the MAGIC output from (Fig. 5D) as a gene regulatory network where arrows connect nuclear proteins to their predicted target genes (encoding other nuclear proteins) with NBS1 highlighted as the major hub for these genes and a direct target of RelA. (**E**) WT and NEMO^DK^ B cells were stimulated with either CD40 + IL-4 or LPS + IL-4 for 2 days and analyzed by qPCR for *Nbn* mRNA. Data represented as in (**A**) (*n* = 3, CD40 + IL-4 *P* = 0.0029). (**F**) Samples from (**B**) were analyzed for *Nbn* mRNA by qPCR. Data represented as in (**B**) (*n* = 3 day 1 *P* = 0.02, day 2 *P* = 0.02, day 3 *P* = 0.0003, day 4 *P* = 0.01). (**G**) Lysates from WT and NEMO^DK^ B stimulated with CD40 + IL-4 over the course of 4 days were analyzed by immunoblot for NBS1 expression with actin as a loading control. Data is represented as in (**C**) (*n* = 3, day 2 = 0.04, day 3 = 0.004, day 4 = 0.006). (**H**) RelA binding peaks in *Nbn* gene locus identified by CUT&Tag analysis from (Fig. 5E). (**I**) The *Nbn* genomic locus (15,957,458–15,958,611) was analyzed using JASPAR, and four putative NF-κB binding sites (indicated by numbered and colored triangles) were selected. Red (κB-#2) and green (κB-#4) lines denote the regions targeted by ChIP-qPCR primers. (**J**) EMSA analysis of lysates from untreated and WT CD40 + IL-4 stimulated (day 4) cells to determine binding to radiolabeled oligonucleotide probes of the 4 *Nbn* putative κB sites with Igκ-κB probe as a positive control. (**K**) ChIP-qPCR analysis of RelA binding at the *Nbn* κB-#2 (*P* = 0.05) and -#4 (*P* = 0.007) regions in untreated and CD40 + IL-4 stimulated (day 4) WT and NEMO^DK^ B cells. Chromatin was immunoprecipitated with anti-RelA or IgG1 isotype control antibodies and quantified by qPCR. Data is shown as fold occupancy, which is the enrichment of binding relative to the % input of each genotype’s untreated RelA sample. “U” represents untreated and “C” represents CD40 + IL-4 treated samples. Data information: statistical analysis was performed using unpaired two-tailed Welch’s *t* test to compare WT and NEMO^DK^ samples (**A**–**C**, **E**–**G**) and ordinary one-way ANOVA with multiple comparisons to test for linear trend and multiple comparisons to untreated controls (**K**). *P* values are indicated: ns, not significant; “*” indicates statistically significant. Source data are available online for this figure.

Our data thus far demonstrate that the double lysine-to-arginine mutation in NEMO^DK^ mice resulted in progressive defects across B cell phenotypes following CD40 + IL-4 stimulation, culminating in reduced CSR and ASC generation. The activation induced cytidine deaminase (AID) protein is required for CSR and is a well-known NF-κB target gene (Park, 2012; Endo et al, 2007). To determine whether AID expression contributes to the CSR defect in NEMO^DK^ B cells, AID protein expression was compared between WT and NEMO^DK^ B cells stimulated with either CD40 + IL-4 or LPS + IL-4. While LPS + IL-4 stimulated samples demonstrated similar levels of AID, NEMO^DK^ B cells showed significantly decreased AID protein compared to WT at day 2 post stimulation (Fig. 6A). This demonstrated that NEMO^DK^ B cells have a CD40 specific defect in AID expression. Time course analysis further confirmed decreased *Aicda* mRNA and AID protein expression in CD40 + IL-4 stimulated NEMO^DK^ B cells compared to WT (Fig. 6B,C).

To further analyze whether there are other key CSR-related factors defective in NEMO^DK^ B cells, we projected the MAGIC output of the purple cluster as a gene regulatory network, in which arrows connect nuclear proteins to their gene targets. This analysis revealed Nijmegen breakage syndrome protein 1 (NBS1) as a major hub for the potential regulatory network and a possible direct target of RelA (Fig. 6D). NBS1 is a DSB repair protein that functions with its complex partners RAD50 and MRE11 and is also critical for CSR reactions post AID-mediated deamination steps (Fan et al, 2025; Petersen et al, 2001). Consistent with this prediction, quantitative reverse transcriptase-PCR (qRT-PCR) analysis confirmed a significant reduction of *Nbn* (encoding NBS1) expression after 2 days of CD40 + IL-4 stimulation in NEMO^DK^ B cells, but not LPS + IL-4 stimulation (Fig. 6E). Time course analysis showed CD40 + IL-4 stimulated NEMO^DK^ B cells also had reduced *Nbn* mRNA and NBS1 protein expression compared to WT (Fig. 6F,G). These results uncovered that NEMO^DK^ B cells fail to coordinately induce AID and NBS1 to promote efficient CSR only after CD40 + IL-4 stimulation.

**Figure EV4:**
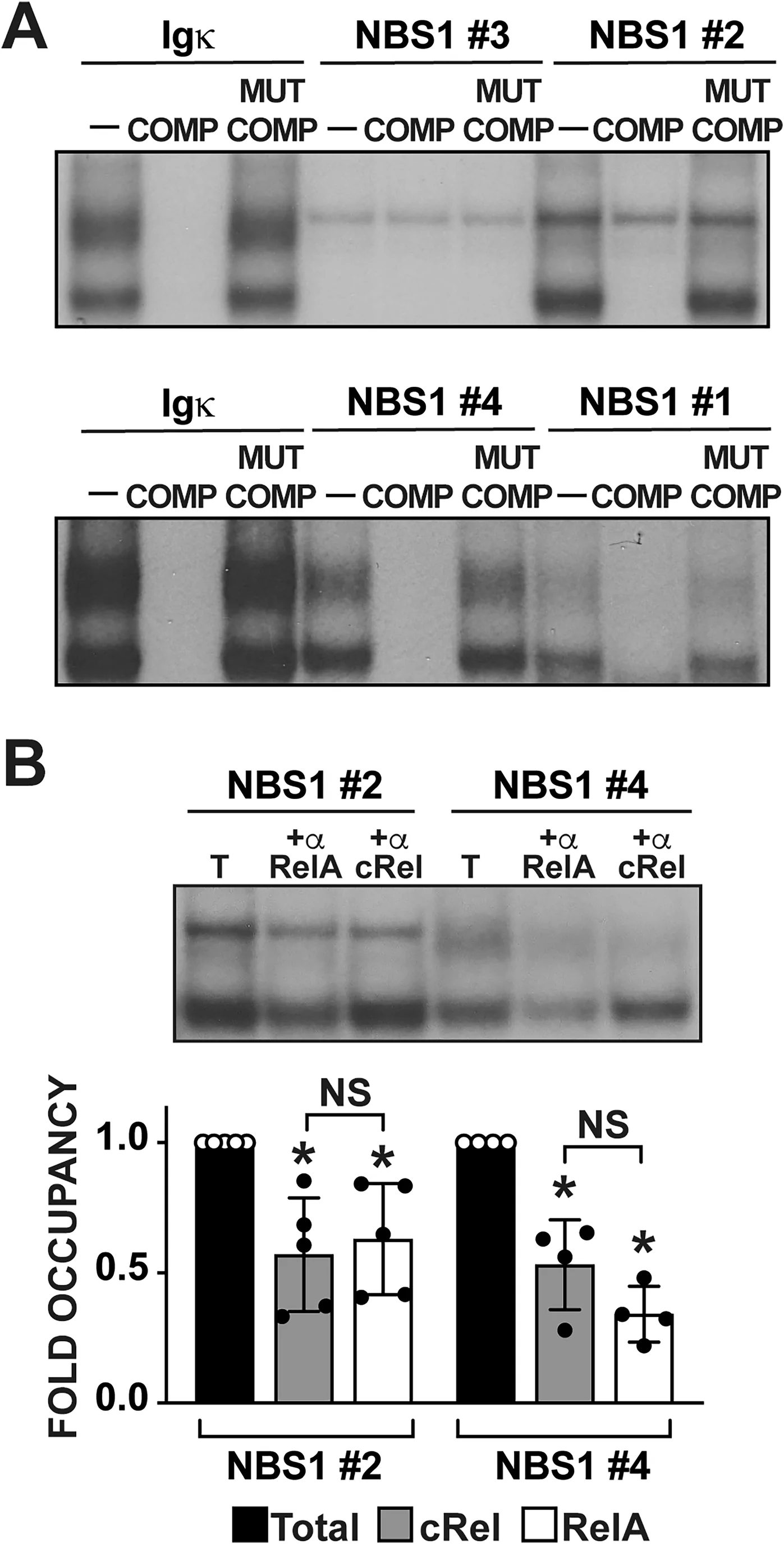
Binding specificity of putative κB sites within *Nbn* locus proximal to start site. (**A**) Competitive binding assay with samples from (Fig. 6J) of the 4 *Nbn* putative κB sites and Igκ-κB with unlabeled 20-fold excess duplexed oligos of authentic WT and mutated κB sites. (**B**) Supershift analysis of the samples from (Fig. 6J) incubated with either RelA or cRel antibodies to determine binding to NBS1 κB-#2 and -#4. Quantification of the remaining bands (leftover NF-κB subunit after shifting) is represented as the relative fold change compared to the “total” sample which contains both RelA and cRel with mean and SD (*n* = 5 for κB-#2, *n* = 4 for κB-#4). For κB-#2, cRel *P* = 0.004 and RelA *P* = 0.01. For κB-#4, cRel *P* = 0.0006 and RelA *P* = 6.74 × 10^−5^. Data information: statistical analysis was performed using unpaired two-tailed Welch’s *t* test to compare cRel and RelA samples and ordinary one-way ANOVA with multiple comparisons to the total samples. *P* values are indicated: “*” indicates statistically significant.

As the MAGIC analysis identified *Nbn* as a potentially novel RelA target gene, and both mRNA and protein analyses showed decreased *Nbn* expression in NEMO^DK^ B cells, we sought to determine whether RelA directly binds to the *Nbn* locus. CUT&Tag analysis revealed peaks within a ~ 1 kb region proximal to the *Nbn* TSS in the CD40 + IL-4 stimulated WT B cells, which was lacking in stimulated NEMO^DK^ B cells or untreated samples, suggesting inducible RelA occupancy at this locus (Fig. 6H). To assess whether this region contains functional NF-κB binding (κB) sites, we queried JASPAR for predicted NF-κB binding motifs and identified four putative κB sites within the 1 kb region (Fig. 6I) (Ovek Baydar et al, 2026). Duplexed oligonucleotide probes corresponding to each site were generated and radiolabeled with ^32^P for EMSA analysis to determine if RelA could directly bind to these sites. Using cell extracts from untreated and CD40 + IL-4 stimulated WT B cells, we observed inducible binding at all 4 sites with distinct migration patterns caused by unique DNA-protein complexes (Fig. 6J). Competition with unlabeled 20-fold excess duplex oligos (both wild-type and mutated) from authentic κB binding sites demonstrated that some of the protein complexes bound to κB-#1, -#2 and -#4 sites were specific, but protein complexes bound to κB-#3 were non-specific (Fig. EV4A). As binding to κB-#1 was very weak, we continued our focus on κB-#2 and -#4 sites. Supershift analyses using RelA and cRel antibodies further demonstrated that the protein complexes bound to κB-#2 and -#4 sites were composed of both NF-κB subunits, RelA and cRel (Fig. EV4B).

To determine whether RelA occupies the *Nbn* locus upon stimulation of B cells with CD40 + IL-4, we designed primer sets spanning the region of the gene surrounding κB-#2 and -#4 sites to perform ChIP-qPCR (Fig. 6I, red and green lines, respectively). ChIP was conducted in untreated and CD40 + IL-4 stimulated WT and NEMO^DK^ B cells (4 days post stimulation) using antibodies against RelA and histone 3 (H3, a positive control), along with an isotype IgG1 negative control. In WT samples, RelA occupancy increased approximately threefold upon CD40 stimulation at the κB-#2 and -#4 sites, whereas IgG1 isotype showed no significant increase (Fig. 6K). In contrast, NEMO^DK^ samples incubated with RelA antibodies did not exhibit a significant increase in occupancy (Fig. 6K). The stimulation-dependent increase in WT samples and lack of enrichment in NEMO^DK^ samples supports inducible recruitment of RelA to the *Nbn* κB-#2 and -#4 regions. Together, the EMSA and ChIP-qPCR results suggest that RelA has the capacity to bind directly to κB elements within the *Nbn* locus upon CD40 + IL4 stimulation.

Overall, these results suggest CD40-induced RelA signaling mediated by NEMO K270/K302 contributes to the full induction of AID and NBS1 to promote efficient CSR. Our study also uncovered that RelA can directly bind to *Nbn* locus, whose gene expression is specifically reduced in CD40 + IL-4 stimulated NEMO^DK^ B cells.

### CD40-induced, sustained canonical NF-κB (RelA) activation is defective in NEMO^DK^ B cells

**Figure 7:**
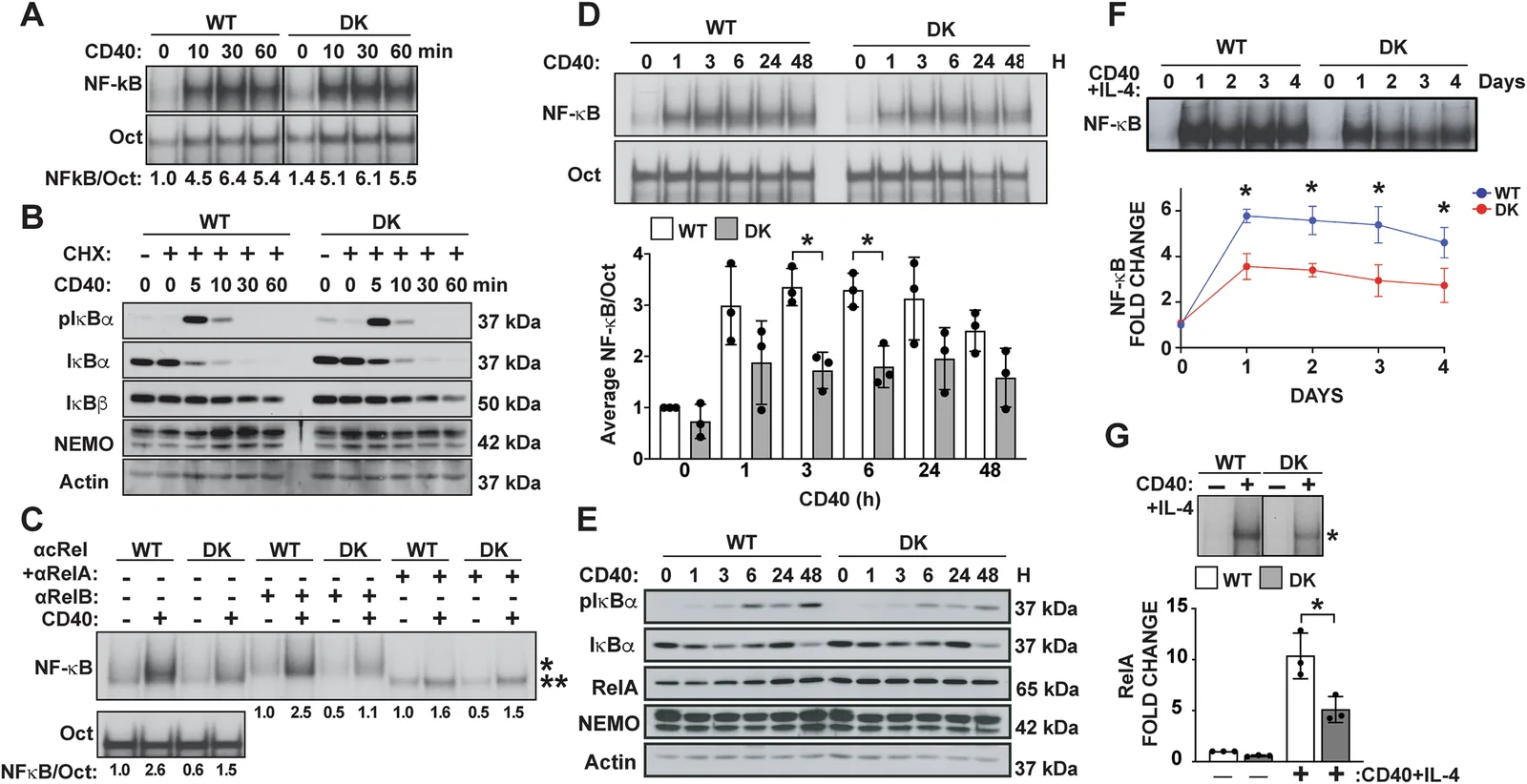
Sustained NF-κB signaling induced by CD40 + IL-4 is defective in NEMO^DK^ B cells. (**A**) Splenic B cells were stimulated with CD40 (2μg/mL) over the period of 1 h and analyzed for NF-κB activation by EMSA. Relative fold change of NF-κB activation normalized to Oct-1 is shown below each lane. The data is representative of three independent experiments. (**B**) Splenic B cells were pretreated with cycloheximide (CHX, 20 μg/mL) for 30 min prior to stimulation with CD40 to clearly detect IκBα/β degradation and analyzed by immunoblot for the indicated NF-κB signaling components and actin as a loading control. (**C**) Splenic B cells treated with CD40 for 24 h were analyzed by supershift assay using the indicated antibodies. Single asterisk marks canonical RelA and cRel complexes; double asterisk marks noncanonical RelB complexes. Relative fold change of NF-κB activation normalized to Oct-1 is shown below each lane. All data shown is representative of three independent experiments. (**D**) Splenic B cells were treated with CD40 over a period of 48 h and analyzed by EMSA for NF-κB and Oct-1 binding. Quantification of EMSA analysis is displayed graphically as the fold change of NF-κB over Oct-1 compared to WT untreated controls with mean and SD (*n* = 3, 3 HR *P* = 0.006, 6HR *P* = 0.01). (**E**) Samples from (**D**) were analyzed by immunoblot for expression of NF-κB signaling components with actin used as a loading control. Note that the lack of cycloheximide here is causing the resynthesis of IκBα. (**F**) Splenic B cells were treated with CD40 (2 μg/mL) and IL-4 (10 ng/mL) over a period of 4 days and analyzed by EMSA. Quantification of EMSA analysis is displayed graphically as a time course plot with relative fold change compared to untreated conditions with mean and SD (*n* = 3, day 1 *P* = 0.0004, day 2 *P* = 0.0005, day 3 *P* = 0.0001, day 4 *P* = 0.002). (**G**) Supershift analysis of WT and NEMO^DK^ splenic B cells stimulated with CD40 + IL-4 over the course of 48 h. Lysates were incubated with antibodies for cRel and RelB to reveal RelA complexes only as denoted by the asterisk. Quantification of RelA complexes displayed graphically as relative fold change compared to untreated conditions with mean and SD (*n* = 3, *P* = 0.04). Data information: statistical analysis was performed using unpaired two-tailed Welch’s *t* test to compare WT and NEMO^DK^ samples (**D**, **G**) and ordinary one-way ANOVA with multiple comparisons to test for linear trend (**F**). *P* values are indicated: “*” indicates statistically significant. Source data are available online for this figure.

Given our transcriptomic analyses identified RelA as the main driver of differentially expressed genes in CD40 + IL-4 stimulated WT vs NEMO^DK^ B cells (Fig. 5D,F, G), we further examined whether canonical NF-κB RelA activation is defective after CD40 stimulation in NEMO^DK^ B cells. Surprisingly, acute canonical NF-κB activation, as measured by EMSA, at 10-, 30-, and 60-min post CD40 stimulation was indistinguishable between WT and NEMO^DΚ^ B cells (Fig. 7A). Accordingly, CD40-induced activation of IκB kinase and subsequent degradation of IκB${{{\rm{\alpha }}}}$ and IκB${{{\rm{\beta }}}}$ at these early time points post-stimulation were also normal in NEMO^DK^ B cells (Fig. 7B). Similarly, delayed noncanonical RelB activation, as determined by supershift analysis, was also similar between NEMO^DK^ and WT B cells following CD40 stimulation (Fig. 7C; Appendix Fig. S5A).

However, further time course analysis revealed NF-κB activation is sustained up to 48 h post CD40 stimulation in WT B cells, but such activity was reduced in NEMO^DK^ B cells starting at 3 h following CD40 stimulation compared to WT (Fig. 7D). Immunoblot analysis confirmed the decrease in canonical NF-κB signaling as evidenced by reduced phosphorylation of IκB${{{\rm{\alpha }}}}$ in NEMO^DK^ B cells (Fig. 7E). Notably, CD40 + IL-4 stimulated WT B cells showed sustained NF-κB activity lasting 4 days of stimulation. However, such activity was significantly reduced in NEMO^DK^ B cells at each time point analyzed (Fig. 7F). This sustained NF-κB activation appears to be unique to CD40 stimulated conditions, as stimulation with IgM (+/− IL-4), P + I or LPS ( + /− IL-4) did not yield strong NF-κB activation 2 days after stimulation, unlike CD40 ( + /− IL-4) (Appendix Fig. S5B). Supershift analyses also showed significant reductions in RelA DNA binding activity at day 2 in NEMO^DK^ B cells relative to WT cells. (Fig. 7G), while RelA protein levels did not change (e.g., Fig. 7E). Thus, RelA activation, rather than its expression, is reduced in CD40-stimulated NEMO^DK^ B cells.

Altogether, NEMO^DK^ B cells showed no significant defect in acute canonical and delayed noncanonical NF-κB activation induced by CD40 stimulation but selectively displayed defects in sustained canonical RelA activation, starting around 3 h post stimulation. Thus, the observed sustained RelA activation defects correlated well with the transcriptomic analysis implicating RelA-selective, genome-wide transcriptional and epigenetic defects in NEMO^DK^ B cells.

### CD40 signaling induces sustained, K270 and K302 dependent, NEMO monoubiquitination

**Figure 8:**
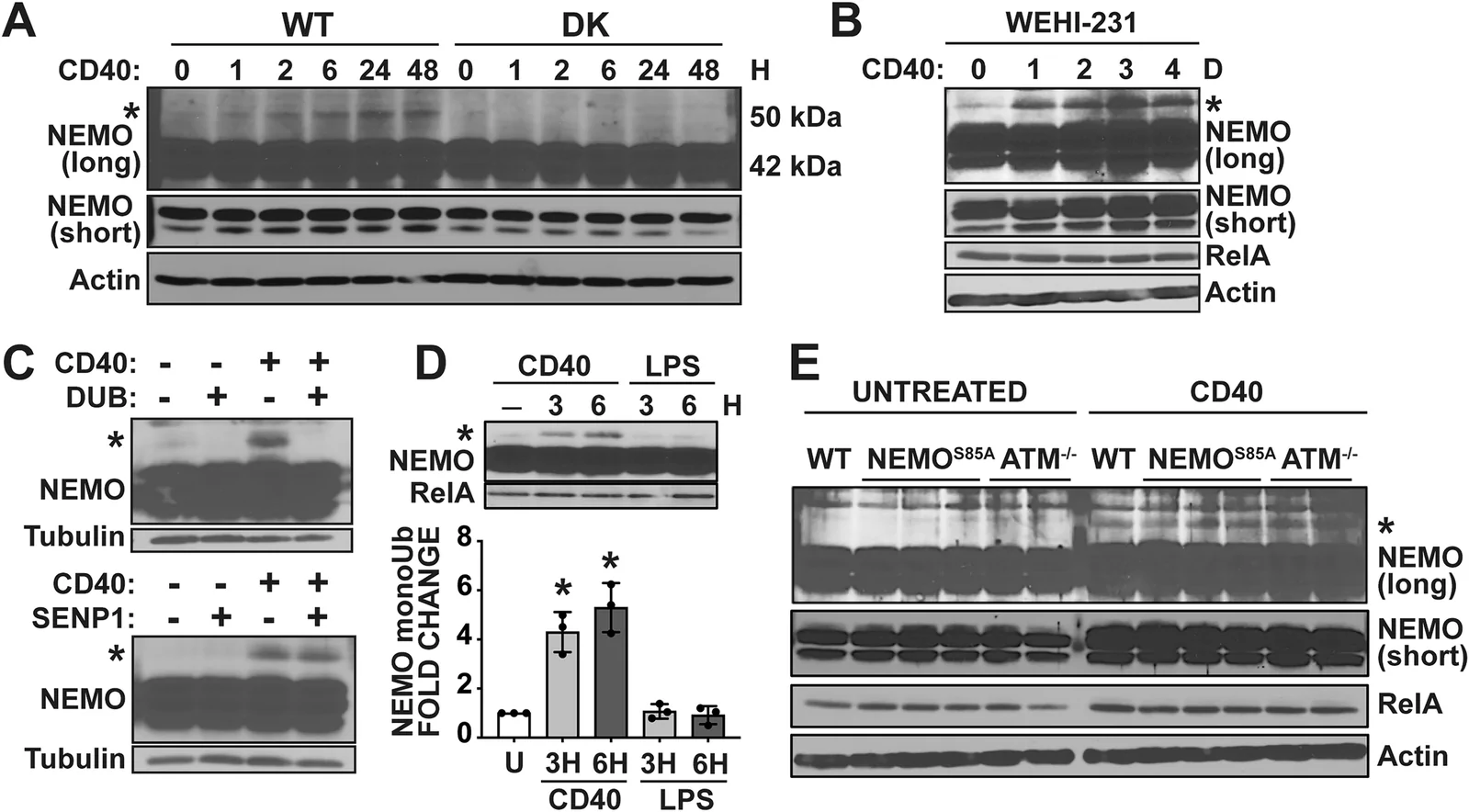
CD40 induces sustained NEMO monoubiquitination dependent on NEMO double lysines but independent of ATM or NEMO S85. (**A**) Splenic WT and NEMO^DK^ B cells stimulated with CD40 (2 μg/mL) for indicated periods were analyzed by immunoblot for NEMO and actin. Blot is representative of three independent experiments; the asterisk denotes the NEMO modification. (**B**) WEHI-231 mouse immature B cells treated with CD40 over 4 days were analyzed by immunoblot for NEMO and actin. The asterisk denotes NEMO modification (*n* = 5). (**C**) WEHI-231 lysates as in (**B**) were incubated with 4 μM USP2 or 4 μM SENP1 and analyzed by immunoblot for NEMO and tubulin. The asterisk denotes NEMO monoubiquitination (*n* = 2). (**D**) WEHI-231 cells were treated with either CD40 (2 μg/mL) or LPS (10 μg/mL) for 3 or 6 h. Lysates were analyzed by immunoblot for NEMO and RelA. The asterisk denotes monoubiquitinated NEMO. Quantification of monoubiquitinated NEMO is represented as relative fold change compared to untreated cells with mean and SD (*n* = 3, 3HR *P *= 0.0002, 6HR *P* = 6.43 × 10^−6^). (**E**) Splenic B cells from WT (*n* = 1), NEMO^S85A^ (*n* = 3), and ATM^−/−^ (*n* = 2) mice were treated with CD40 for 24 h and lysates were analyzed by immunoblot for NEMO, RelA, and actin. The asterisk denotes monoubiquitinated NEMO. Data information: statistical analysis was performed using ordinary one-way ANOVA with multiple comparisons to the untreated condition. *P* values are indicated: “*” indicates statistically significant. Source data are available online for this figure.

Because the mutated lysine sites in NEMO^DK^ mice have been implicated in the PTM of NEMO, such as sumoylation and ubiquitination, to mediate NF-κB signaling, we next examined whether CD40 stimulation caused such modifications of NEMO in WT but not NEMO^DK^ B cells (Huang et al, 2003; McCool and Miyamoto, 2012). Whole cell lysates from CD40-stimulated WT and NEMO^DK^ B cells were analyzed by NEMO immunoblot. Interestingly, we observed the sustained accumulation of a novel CD40-inducible NEMO PTM band of ~50 kDa migrating above the bulk 42 kDa NEMO in WT B cell lysates, which was completely absent in NEMO^DK^ samples (Fig. 8A). This NEMO PTM band corresponded with CD40-induced NF-κB activation over a similar time course (Fig. 7D). Remarkably, CD40 stimulation of the WEHI-231 immature B cell line also resulted in the NEMO modification that was sustained over the course of 4 days (Fig. 8B).

**Figure EV5:**
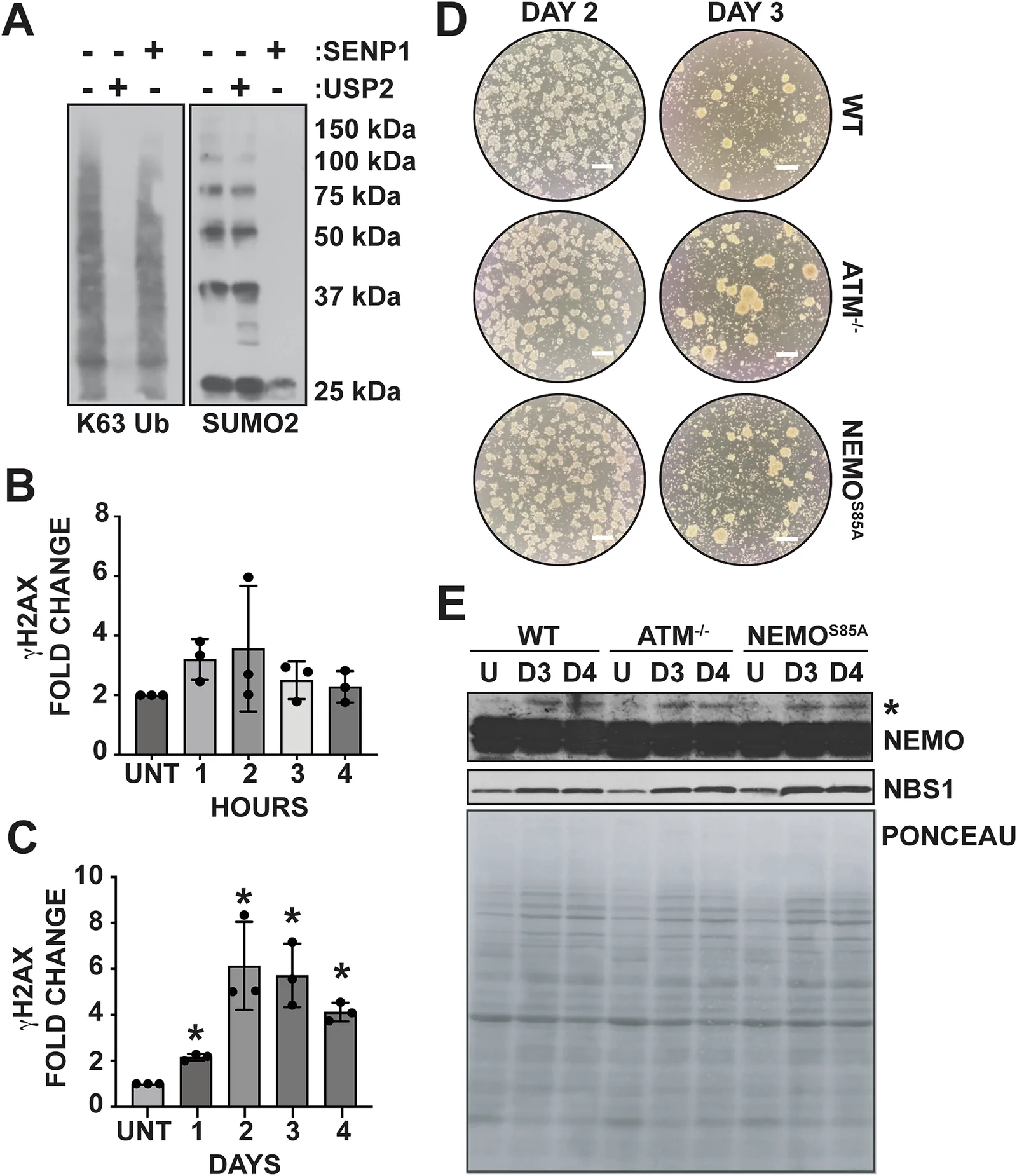
CD40-induced responses are independent of DNA damage-ATM-NEMO signaling. (**A**) Recombinant K63-linked polyubiquitin or SUMO-2 chains were incubated with USP2 or SENP1 and analyzed by immunoblot with anti-K63 Ub or anti-SUMO2 antibodies (*n* = 2). (**B**) WT primary splenic B cells were stimulated with CD40 + IL-4 over the course of 4 h, stained for γH2AX, and analyzed by flow cytometry. Data is represented as the MFI fold change for each condition normalized to the untreated control, and values presented as bar graphs showing the mean relative fold change and SD (*n* = 3). (**C**) WT primary splenic B cells were stimulated with CD40 + IL-4 over the course of 4 days and analyzed as in (**B**) (*n* = 3, day 1 *P* = 0.005, day 2 *P* = 0.04, day 3 *P* = 0.03, day 4 *P* = 0.006). (**D**) Representative images of HA from WT (*n* = 3 pooled), ATM^−/−^ (*n* = 3 pooled) and NEMO^S85A^ (*n* = 3 pooled) stimulated with CD40 + IL-4 for 4 days. HA images from days 2 and 3 are displayed, scale bar on bottom right represents 100 μm (*n* = 1). (**E**) Lysates from day 3 and 4 samples from (**D**) were analyzed for NEMO and NBS1, with ponceau staining for loading control. The asterisk denotes monoubiquitinated NEMO (*n* = 1/genotype). Data information: statistical analysis was performed using ordinary one-way ANOVA with multiple comparisons to the untreated condition. “*” indicates statistically significant.

Due to previous data demonstrating the requirement of the equivalent lysine residues in human NEMO for SUMO1 and monoubiquitin modifications, along with the small size shift (~ 8 kDa) observed by NEMO immunoblot, we hypothesized the modifications might be due to monoubiquitin (Huang et al, 2003; McCool and Miyamoto, 2012). To test this, we first performed immunoprecipitation of ubiquitin in denatured protein extracts, followed by Western blot analysis of NEMO. However, the presence of large amounts of Ig heavy chain (50 kDa) that comigrated with modified NEMO obscured our analysis. Thus, we next performed in vitro deconjugation assays using recombinant deubiquitinase (ubiquitin specific protease 2, USP2) and SUMO protease (sentrin specific protease 1, SENP1) as a specificity control. Incubation of CD40-stimulated cell lysates with USP2, but not SENP1, eliminated the CD40-induced NEMO PTM (Fig. 8C). Specificity of corresponding enzymatic actions was validated by deconjugation of recombinant polyubiquitin and SUMO2 chains, respectively (Fig. EV5A). Based on the shift of ~8 kDa, approximately the size of a ubiquitin (8.5 kDa), in the migration of modified NEMO and sensitivity to deubiquitinase, we conclude that this NEMO PTM is due to monoubiquitination. Consistent with the CD40 specificity of NEMO^DK^ B cell defects, NEMO monoubiquitination was induced by CD40 but not by LPS in WEHI-231 cells (Fig. 8D).

### CD40-induced NEMO monoubiquitination does not involve DNA damage-ATM-NEMO signaling

In response to DNA DSBs, ATM is activated and phosphorylates NEMO at S85, allowing NEMO to be monoubiquitinated to trigger NF-κB activation (Wu and Miyamoto, 2008; McCool and Miyamoto, 2012). Thus, inhibition of ATM or mutation of NEMO S85 prevents efficient activation of NF-κB in response to genotoxic stress but permits normal NF-κB activation in response to TNF${{{\rm{\alpha }}}}$ and LPS. If CD40 stimulation induces DSBs, this could be the source of the observed NEMO monoubiquitination. While CD40-induced monoubiquitination is observed as early as 2h following stimulation (Fig. 8A), DSB as measured by ${{{\rm{\gamma }}}}$H2AX signals using flow cytometry was not significantly induced at such early time points (0–4 h, Fig. EV5B), pointing to an alternative mechanism at play during these early time points after CD40 stimulation. However, ${{{\rm{\gamma }}}}$H2AX signals were significantly increased at 1–4 days after CD40 + IL-4 stimulation of B cells with the peak detected at days 2-3 (Fig. EV5C), suggesting the possibility that DSBs might be playing a role in these later time points where maximal cell proliferation and CSR take place.

To formally test the role of DSB-induced ATM activation and NEMO S85 phosphorylation in CD40-induced NEMO monoubiquitination, we next analyzed ATM^−/−^ and newly engineered NEMO^S85A^ mice (see Materials and Methods for details). We stimulated splenic B cells from WT, ATM^−/−^, and NEMO^S85A^ mice with CD40 for 24 h and found similar induction of NEMO monoubiquitination in the B cells from all 3 mice (Fig. 8E). Induction of similar monoubiquitination at days 3 and 4 after CD40 + IL-4 stimulation was also confirmed in WT, ATM^−/−^ and NEMO^S85A^ B cells, along with normal HA and NBS1 induction (Fig. EV5D,E).

Overall, these results reveal a sustained accumulation of CD40-induced NEMO monoubiquitination that is entirely dependent on intact lysine 270 and 302 residues. Subsequent HA and NBS1 induction occur independently of DNA damage-ATM-NEMO signaling. Moreover, these mutant phenotypes are specific to CD40 stimulation and not observed with LPS stimulation.

### CD40-induced mitochondrial-ROS promotes NEMO monoubiquitination and sustained NF-κB activation

**Figure 9:**
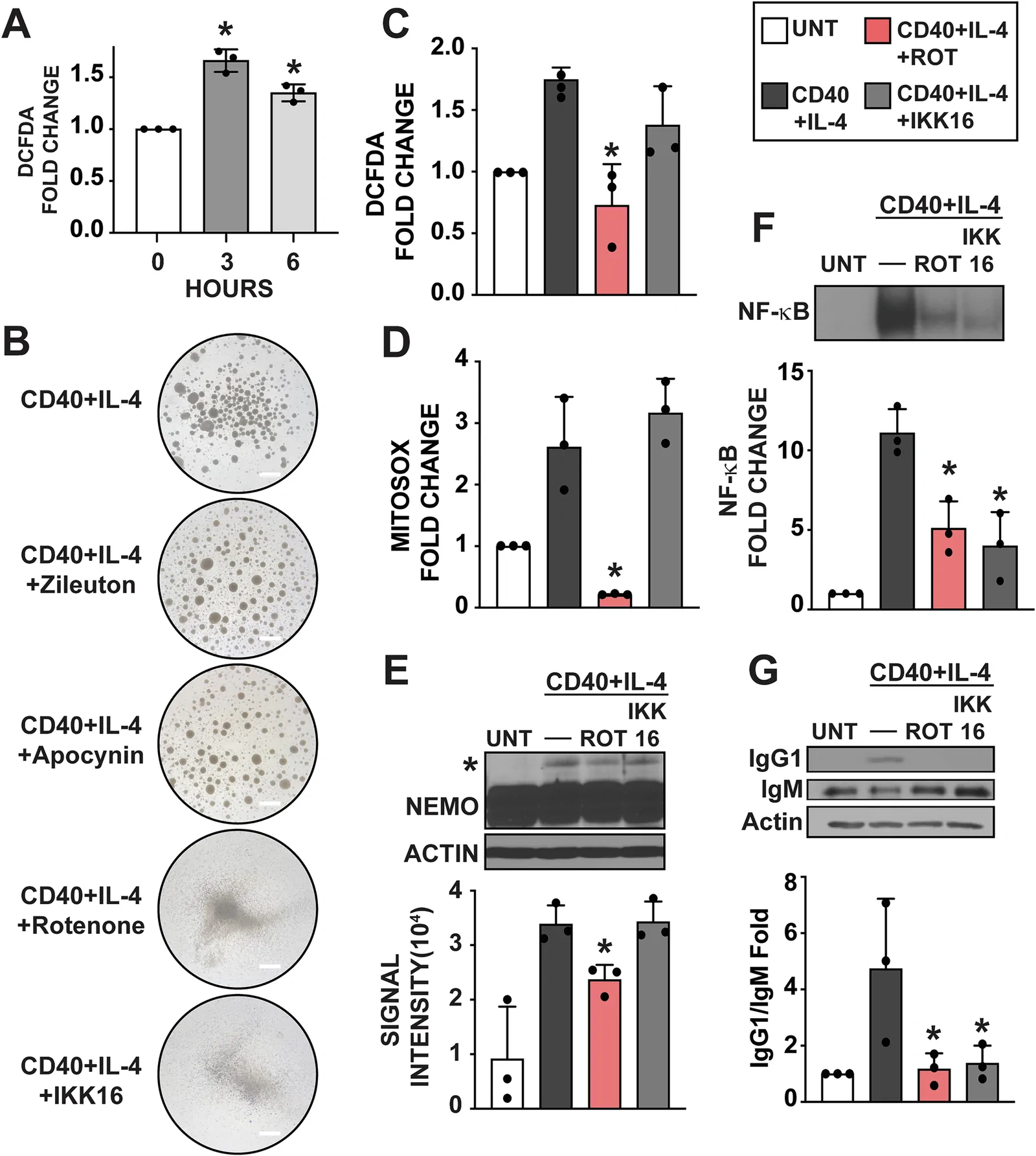
CD40-induced mitochondrial ROS is required for sustained monoubiquitination and downstream events. (**A**) WT splenic B cells were treated with CD40 + IL-4 for 3 and 6 h and stained with H_2_DCFDA and analyzed by flow cytometry. Data is represented as the MFI fold change for each condition normalized to the untreated control, and the values are presented as bar graphs with mean and SD (*n* = 3, 3HR *P* < 0.001, 6HR *P* = 0.003). (**B**) Representative images of HA formation in CD40 + IL-4 stimulated WT B cells cotreated with zileuton (50 μM), apocynin (40 μM), rotenone (0.5 μM), or IKK16 (0.5 μM) for 4 days. Scale bar on the bottom right represents 400 μm. (**C**) WT B cells treated with either CD40 + IL-4 alone or cotreatment with rotenone or IKK16 for 24 h, stained with H_2_DCFDA, and analyzed by flow cytometry. Data is represented as the MFI fold change for each condition normalized to the untreated control, and the values are presented as bar graphs showing the mean and SD (*n* = 3, *P* = 0.002). (**D**) Cells from (**C**) were stained with MitoSOX^TM^ Red and analyzed by flow cytometry. Data is represented as described in (**C**, *P* = 0.0009). (**E**) Lysates were made from the cells treated as in (**C**) and analyzed by immunoblot for NEMO and actin, with the asterisk denoting monoubiquitinated NEMO. Quantification of monoubiquitinated NEMO is represented as fold change normalized to the untreated condition mean with SD (*n* = 3, *P* = 0.02). (**F**) Lysates from (**E**) were analyzed by EMSA for NF-κB activation (upper) and quantification (lower) is represented as in (**E**, *P* = 0.003). (**G**) WT B cells treated with either CD40 + IL-4 alone or cotreatment with rotenone or IKK16 for 4 days were collected, and analyzed by immunoblot for IgG1, IgM, and actin (upper). Quantification (lower) of class switching is represented as the relative fold change of IgG1 over IgM compared to untreated cells with mean and SD (*n* = 3, ROT *P* = 0.0258, IKK16 *P* = 0.0342). Data information: statistical analysis was performed using ordinary one-way ANOVA with multiple comparison to the “0” time point (**A**) or CD40 + IL-4 alone (**C**–**G**). *P* values are indicated: “*” indicates statistically significant. Source data are available online for this figure.

Since DNA damage-ATM-NEMO-NF-κB signaling failed to account for B cell-intrinsic defects seen in CD40-stimulated NEMO^DK^ B cells, we next considered the possible role of ROS, another source of intracellular stress that can also activate NEMO K270/K302-dependent NF-κB signaling (Wuerzberger-Davis et al, 2007). CD40 stimulation in B lymphoma cell lines have been reported to rapidly induce ROS to support NF-κB signaling and downstream effector functions but the mechanism involved in primary B cells is unclear (Ha and Lee, 2004; Ha et al, 2011). To determine whether ROS acts as a signaling intermediate linking CD40 stimulation to NF-κB activation through NEMO K270/K302, we first examined whether CD40 induces ROS at early time points corresponding to the onset of the NF-κB signaling defect observed in NEMO^DK^ B cells. Intracellular ROS was measured using 2’,7’-dichlorodihydrofluorescein diacetate (H_2_DCFDA), which becomes fluorescent upon oxidation by intracellular H₂O₂. Flow cytometric analysis revealed significant ROS induction at 3 and 6 h following CD40 + IL-4 stimulation in WT B cells (Fig. 9A). Importantly, CD40 + IL-4 stimulated NEMO^DK^ B cells exhibited comparable ROS levels at 6 h post stimulation (Appendix Fig. S6A), and extended time-course analysis demonstrated sustained ROS signals in both WT and NEMO^DK^ cells (Appendix Fig. S6B). Despite defective NF-κB activation in NEMO^DK^ B cells, ROS induction was normal, suggesting that ROS production occurs upstream of the signaling defect and may function as an intermediate signal promoting NEMO K270/K302-dependent sustained NF-κB activation.

To directly test the contribution of ROS to CD40-induced NF-κB signaling, we utilized the antioxidant N-acetyl cysteine (NAC). B cell HA formation was first used as a rapid functional readout of NF-κB activity. NAC significantly diminished ROS accumulation at 3 h post stimulation following CD40 + IL-4 stimulation (Appendix Fig. S6C) and prevented HA formation (Appendix Fig. S6D). Consistent with this reduction, NAC treatment abolished NEMO monoubiquitination (Appendix Fig. S6E) and NF-κB activation (Appendix Fig. S6F) at 24 h following CD40 stimulation. Furthermore, NAC prevented CSR, as measured by decreased IgG1 production relative to IgM at day 4 of stimulation (Appendix Fig. S6G). Collectively, pharmacologic inhibition of ROS prevented key signaling and functional outcomes of CD40 + IL-4 stimulation in WT B cells, supporting a requirement for ROS in sustaining CD40-mediated NF-κB responses.

Because we needed to use high levels of NAC to observe the above effects in B cells, we sought to further investigate the physiological source(s) of ROS following CD40 stimulation, which leads to NEMO monoubiquitination and downstream B cell phenotypes. CD40 signaling has been reported to generate ROS through multiple pathways, including 5-lipoxygenase (5-LO), NADPH oxidase, and mitochondrial oxidative phosphorylation (OXPHOS) (Ha et al, 2011; Ha and Lee, 2004; Herb et al, 2019). To identify the relevant ROS source contributing to CD40-dependent NF-κB activation, WT B cells were treated with pathway-specific inhibitors: zileuton (5-LO), apocynin (NADPH oxidase), and rotenone (mROS) (Helmy et al, 2018; Petrônio et al, 2013; Herb et al, 2019). HA formation was again used as a surrogate readout for NF-κB activity, with IKK16 serving as a positive control for pathway inhibition. Whereas zileuton and apocynin had no effects on HA formation, rotenone inhibited HA formation to a degree comparable to IKK16 (Fig. 9B), prompting further analysis of mROS. DCFDA measurements demonstrated that rotenone significantly reduced cytosolic ROS following CD40 + IL-4 stimulation (Fig. 9C). In contrast, IKK16 did not affect ROS levels, indicating that ROS production occurs upstream of IKK activation. Because rotenone primarily targets mitochondrial complex 1, mitochondrial superoxide was measured using MitoSOX™ Red. Rotenone reduced mROS to near-baseline levels, demonstrating a more pronounced effect than observed with cytosolic ROS, consistent with a selective inhibition of mROS production (Fig. 9D). Rotenone treatment also significantly decreased NEMO monoubiquitination (Fig. 9E), NF-κB activation (Fig. 9F), and IgG1 CSR (Fig. 9G) following CD40 + IL-4 stimulation. Notably, IKK16 impaired NF-κB activation and CSR without affecting NEMO monoubiquitination, further supporting that NEMO modification occurs upstream of IKK activation.

Altogether, these data support a model in which mitochondrial ROS contributes to CD40-induced NEMO monoubiquitination in a manner dependent on intact K270 and K302, thereby promoting sustained NF-κB RelA signaling required for efficient HA and CSR in B cells.

## Discussion

In this study, we discovered a role for NEMO K270/K302 in promoting efficient GC and antibody responses in vivo, and CD40-mediated responses ex vivo using a newly engineered NEMO^DK^ mouse model. In NEMO^DK^ mice, lymphocyte development and maturation appears normal, but mixed bone marrow chimera and LCMV infection experiments revealed a B cell-intrinsic defect in GC responses with decreased populations of GC and class switched B cells and impaired virus specific antibody production. In contrast, T cell populations and functions, particularly T_FH_ cells, were normal in NEMO^DK^ mice. Upon ex vivo analysis, using CD40 agonist to mimic T_FH_-B cell interaction in vivo, we discovered NEMO^DK^ B cells  had defects in NEMO monoubiquitination, sustained NF-κB RelA activation, HA formation, proliferation, CSR, and antibody secreting cell generation. In contrast, NEMO^DK^ B cells had normal responses to other canonical B cell activators, including LPS. Multiple independent transcriptomic and epigenomic analyses revealed NEMO K270/K302 mediated RelA-driven transcription to affect downstream CD40-induced B cell responses. Pharmacological inhibition of ROS, and more specifically, mROS, phenocopied the NEMO^DK^ defects, implicating a role for mROS in modulating CD40-induced sustained NEMO monoubiquitination, RelA activation, and subsequent responses. In contrast, CD40 stimulated B cells from ATM^−/−^ and NEMO^S85A^ mice had comparable NEMO monoubiquitination, HA formation, and NBS1 expression as WT mice, highlighting DNA damage-ATM-NEMO signaling has no role in CD40-induced sustained RelA activation. Moreover, we demonstrated for the first time that RelA can inducibly bind to two putative κB sites on the *Nbn* locus upon CD40 stimulation, in a manner that is promoted by NEMO K270/K302. These findings highlight the importance of NEMO K270/K302 in mediating CD40-induced sustained RelA activation to promote efficient T cell-dependent B cell responses both in vivo and ex vivo.

While the well-established CD40-induced acute canonical and sustained noncanonical NF-κB pathways remain intact in NEMO^DK^ B cells, our mutant mice revealed a mechanistically distinct third pathway. Acute canonical NF-κB activation downstream of CD40 is driven by receptor-proximal signaling mediated by TRAF6 and TAK1 leading to the presence of phosphorylated IκB${{{\rm{\alpha }}}}$ within minutes of stimulation, whereas our findings suggest that sustained RelA signaling involves site-selective NEMO monoubiquitination (Liu et al, 2017; Arcipowski and Bishop, 2012; Merino-Vico et al, 2024). This PTM transiently appears ~90 min following DNA damage induced by VP16 treatment (Huang et al, 2003). In contrast, NEMO^DK^ B cells demonstrated that CD40 signaling is unlike DNA damage signaling because NEMO monoubiquitination is induced 1–3 h after stimulation and sustained throughout the duration of stimulation, even up to 4 days. Moreover, CD40 stimulation results in more robust NF-κB activation compared to IgM and P+I (Appendix Fig. S5B). These observations support a model in which CD40 stimulation induces sustained signaling dependent on monoubiquitination of NEMO at K270/K302 to regulate the magnitude and duration of RelA activity required for optimal B cell-intrinsic humoral responses. In addition, our data show that CD40 stimulation generates sustained mROS that promotes prolonged NEMO monoubiquitination and maintains NF-κB- activation. This reveals a previously unrecognized mode of NEMO regulation, whereas  most reported NEMO post-translational modifications are only transiently induced (Jackson et al, 2013; Huang et al, 2003; Wu et al, 2006). Notably, sustained NF-κB signaling has largely been described in pathological settings such as cancer or autoimmune disease, where chronic pathway activation contributes to aberrant cell survival or immune responses, and studies examining long-term NF-κB dynamics during physiological B cell activation remain limited (Mao et al, 2025). One study of CD40-stimulated B cells reported sustained RelA nuclear localization but only up to 24 h following stimulation (Zarnegar et al, 2004). Our findings substantially extend this temporal framework by demonstrating that canonical NF-κB (RelA) signaling can remain sustained for at least four days following CD40 + IL-4 stimulation, the longest time point examined in this study. We also observed CD40-induced cRel activation as revealed by supershift assays. and found cRel also had the capacity to bind to *Nbn* κB elements,  potentially contributing to *Nbn* gene regulation under certain conditions. However, we did not specifically evaluate the contribution of cRel since all of our unbiased transcriptomic and epigenetic analyses pointed to RelA as the main contributor to CD40-induced NEMO^DK^ cell defects. Collectively, these results reveal a previously unrecognized mROS-dependent regulatory mechanism that maintains prolonged RelA signaling in CD40-activated B cells and identify NEMO K270/K302 as critical regulatory residues required to sustain RelA activity during humoral immune responses.

Notably, the double lysine mutation of NEMO produced a striking CD40-specific defect while leaving LPS- and IgM-induced responses largely intact, despite all three stimuli activating canonical NF-κB signaling. This specificity was unexpected and suggests that NEMO K270/K302-dependent NF-κB activation requires signaling features uniquely engaged downstream of CD40. Unlike LPS or BCR stimulation, CD40 robustly activates both the canonical and noncanonical NF-κB pathways and promotes germinal center-associated processes such as HA formation (Zarnegar et al, 2004). A previous study reported that combinatorial treatment with LPS and BAFF, which induces canonical (LPS) and noncanonical (BAFF) NF-κB signaling, failed to induce HA formation at 24 h, but generated smaller HAs by 72 h compared to those induced by CD40 (Zarnegar et al, 2004). These findings suggest that HA formation requires not only concurrent canonical and noncanonical NF-κB signaling but also a sufficient duration and/or magnitude of signaling. Moreover, another study showed LPS + IL-4 stimulation induces ROS to drive B cell fate determination, but this ROS declines after 48 h (Jang et al, 2015). However, we found in our studies that CD40 + IL-4 stimulation maintains a high level of ROS for at least 4 days. Furthermore, although costimulation of BCR and BAFF in primary splenic B cells enhanced the magnitude and duration of cRel DNA binding, RelA activity was rapidly induced and returned to basal levels within 6 h (Almaden et al, 2016). In contrast, our study suggests sustained RelA activity following CD40 stimulation. We also noted IgM (BCR) stimulation did not result in proliferation defects in NEMO^DK^ B cells. This aligns with previous literature establishing that IgM alone is unable to induce efficient proliferation and requires cofactors, such as IL-4, to promote proliferation (Sidman and Unanue, 1978; Paul et al, 1986). In contrast, CD40 stimulation alone is able to induce proliferation in B cells, with cofactors, such as IL-4, enhancing proliferation (Lee et al, 2003; Guo et al, 2021; Tawab et al, 2004; Rousset et al, 1991, 1991). Altogether, our data, and prior published studies, suggest CD40 stimulation is unique in its ability to induce sustained RelA-driven, NF-κB activation that is mediated by prolonged mROS generation. The proficient induction of ROS, but absence of monoubiquitination and sustained NF-κB activation, in NEMO^DK^ B cells highlights the requirement of NEMO K270/K302 in mediating these downstream signaling events. Together, these findings suggest that NEMO K270/K302 define a CD40-specific mechanism for sustaining robust RelA signaling, providing new insight into how different stimuli engaging the NF-κB signaling system can produce distinct functional outcomes in B cells and help explain how CD40 uniquely supports GC-associated B cell functions.

Previous studies have established that ROS promotes proliferation and CSR in B cells, processes that are also driven by CD40-NF-κB signaling (Wheeler and Defranco, 2012; Guikema et al, 2010; Lee, 2003; Zarnegar et al, 2004; Wang et al, 2006). ROS is also reported to activate NF-κB to enhance immune cell function, such as promoting T cell responses and act as microbicidal agents in phagocytic cells (Ginn-Pease and Whisler, 1998; Canton et al, 2021). However, the mechanistic relationship linking CD40-induced ROS to NF-κB activation in B cells remains unclear. Our findings bridge this gap by demonstrating that CD40-induced mROS promotes NF-κB  activation and provide temporal and mechanistic insight into how this signaling axis contributes to downstream processes required for effective humoral responses. Previous studies examining CD40-induced ROS implicated the 5-LO and NADPH oxidase pathways using lymphoma cell lines, which limits the relevance to primary B cell biology and may account for why the inhibition of these pathways failed to prevent HA formation in CD40-stimulated WT B cells in our study (Ha and Lee, 2004; Ha et al, 2011). Other literature has shown inconsistencies in how ROS influences NF-κB activity, specifically whether it inhibits or promotes NF-κB activation. This could be a result of using differing cell types, such as macrophages or human lymphoma cells, as well as agonists, such as TNF or LPS (Herb et al, 2019; Jamaluddin et al, 2007; Agrahari et al, 2022; Jang et al, 2015). Our findings clarify that CD40-stimulated primary B cells induce ROS to activate NF-κB within 3 h of stimulation and maintain ROS for 4 days to sustain RelA signaling through NEMO monoubiquitination, promoting HA, proliferation, CSR and ASC production. The use of rotenone in our study also implicates mROS as the primary source of ROS in this system, indicating that mROS, rather than 5-LO or NADPH oxidase pathways, predominates in regulating RelA signaling during CD40 activation of primary B cells. These findings may also inform how ROS regulates signaling in other immune cells, where ROS has been implicated in shaping activation and effector responses, but the mechanisms linking ROS production to potential sustained transcriptional signaling remain incompletely defined.

Another important feature of the NEMO^DK^ mouse model is that it represents the only viable mouse model examining a NEMO PTM that produces a measurable NF-κB signaling phenotype. Previous genetically modified mouse models have been unsuccessful in revealing the physiological roles of NEMO PTMs in NF-κB signaling, such as those incorporating germline mutations, K392R and S85A (Alhawagri et al, 2012; Glynn et al, 2024). While insertion of K285/K399R mutant human NEMO cDNA into the *Nemo* locus resulted in embryonic lethality, similarly inserted wild-type cDNA needs to be analyzed given that NEMO knockout mice are also embryonic lethal (Schmidt-Supprian et al, 2000; Jun et al, 2013). In contrast, our NEMO^DK^ mice provide new insights into the critical function of NEMO K270/K302 in sustaining mROS-mediated RelA signaling in CD40-stimulated B cells and humoral immunity. Moreover, our NEMO^DK^ mice may also provide a better understanding of physiological roles of DNA damage-induced NF-κB activation as DNA damage-induced NF-κB signaling was significantly inhibited in several tissues and cell types analyzed, although this inhibition was incomplete. Similarly, although the double lysine mutation impaired CSR, it did not abolish it, highlighting a therapeutic window to selectively modulate aberrant CD40–NF-κB signaling without broadly disrupting normal immune function. These findings establish the NEMO^DK^ mouse model as a valuable platform for defining how NEMO PTMs regulate NF-κB signaling in immune cells and how dysregulation of these mechanisms may contribute to inflammatory and immune-mediated diseases.

Although our data strongly support a role for mROS as a key intermediate factor sustaining NEMO monoubiquitination and RelA activity following CD40 stimulation, they do not definitively establish mROS sufficiency or directly link it to the modification of NEMO at K270/K302. Experiments testing ROS sufficiency using physiological ROS-generating systems, such as glucose oxidase–mediated H₂O₂ production, as well as examining potential mechanisms including ROS-induced inhibition of deubiquitinases, could further clarify how ROS promotes NEMO monoubiquitination in this system (Panayiotidis et al, 1999; Lee et al, 2013). Investigation of the role of known ubiquitin ligases that promote NEMO monoubiquitination, specifically TRAF6, cIAP1 and TRIM37, would further clarify the mechanism of this CD40-induced NEMO modification (Hinz et al, 2010; Wu et al, 2018). Finally, because both lysine residues are mutated in NEMO^DK^ mice, our study cannot distinguish the individual contributions of K270 and K302. Although previous in vitro work showed that mutation of either residue alone was insufficient to block NF-κB activation by genotoxic agents, it remains unclear whether one or both residues are required to sustain NEMO monoubiquitination and RelA activity downstream of CD40 in primary B cells (Huang et al, 2003). It has been demonstrated that TRIM37 specifically induces monoubiquitination of K309 in human NEMO (K302 mouse equivalent) and this mutation was sufficient to prevent NF-κB activation by genotoxic agents in esophageal cancer cell lines, further highlighting species and cell type differences in lysine requirements to mediate NF-κB signaling (Wu et al, 2018). Thus, addressing above questions will be important for defining the precise molecular mechanisms by which NEMO regulates sustained RelA signaling in B cells.

Mutations affecting NEMO and the CD40 pathway have well-established consequences for human immunity. Patients with X-linked hyper-IgM syndrome with hypohydrotic ectodermal dysplasia (XHM-ED) harboring missense mutations in the zinc-finger (ZF) domain of NEMO exhibit defective CD40L-mediated IκBα degradation and impaired CSR, whereas innate immune responses to LPS remain largely intact (Jain et al, 2001). Similarly, hypomorphic ZF mutations in XHM-ED patients have been associated with defective somatic hypermutation (SHM), CSR, and cRel activation following CD40 + IL-4 stimulation despite normal induction of AID (Jain et al, 2004). These observations highlight a selective requirement for intact NEMO signaling in CD40-driven adaptive B cell responses. Parallel observations have been reported in patients with mutations in the CD40 pathway. Individuals with CD40 or CD40L deficiency exhibit reduced serum IgG, IgA, and IgE levels with normal or elevated IgM, impaired antigen-specific antibody responses, and reduced numbers of class-switched memory B cells despite normal total B cell counts (Dunn and de la Morena, 1993; Leite et al, 2020). In one case, a patient with normal surface expression of CD40 carried a mutation inserting 37 additional amino acids into the cytoplasmic domain, disrupting receptor internalization required for effective downstream signaling (Murguia-Favela et al, 2017). Clinically, this patient presented with recurrent infections, undetectable IgG and IgA titers, elevated IgM, and an inability to generate protective vaccine-specific antibody responses despite full immunization. Notably, B and T cell numbers and lymphocyte proliferative responses remained intact, indicating a functional signaling defect rather than a developmental abnormality. Collectively, these observations illustrate how distinct mutations affecting CD40 binding, receptor trafficking, or downstream signaling components can selectively impair humoral immunity. Although mutations in human NEMO at the corresponding residues K277 and K309 have not been reported, our findings provide mechanistic insight into how NEMO regulates CD40-induced NF-κB activation and downstream processes such as B cell proliferation and CSR.

Together, our findings support a model in which CD40 stimulation promotes a third NF-κB signaling pathway, in addition to well-established acute canonical and delayed noncanonical. This third pathway specifically involves sustained mROS production to facilitate sustained NEMO monoubiquitination at K270/K302, thereby enabling prolonged RelA signaling required for efficient B cell HA, proliferation, CSR, and humoral responses. Thus, these findings identify a previously unrecognized regulatory mechanism within the CD40-NF-κB signaling axis, providing new insight into how dysregulated NF-κB signaling contributes to immune dysfunction and suggesting potential strategies to control pathological immune activation while preserving protective immune responses.

## Methods

**Table:** Reagents and tools table

Reagent/resource	Reference or source	Identifier or catalog number
**Experimental models**		
ATM knockout Mice	Jackson labs	008536
C57BL/6J	Jackson labs	000664
B6.129S2-*Ighm*^*tm1Cgn*^/J (µMT mice)	Jackson labs	002288
**Recombinant DNA**		
PL253	Liu et al, 2003	
PL452	Liu et al, 2003	
BAC13D21	BACPAC Resource, Center, CA	
**Antibodies**		
Anti-Actin (goat)	Santa Cruz Biotechnology	sc-1616
Anti-β-actin	Sigma-Aldrich	A2228
Anti-AID (ZA001)	ThermoFisher	2500
Anti-B220-APC	eBioscience	17-0452
Anti-CD4 Monoclonal Antibody (GK1.5), APC	ThermoFisher	17-0041
Anti-CD8a Monoclonal (53-6.7), PE	ThermoFisher	12-0081-83
Anti-CD21/CD35, mouse, PE		
CD23 Monoclonal Antibody (B3B4), PE-Cyanine7	ThermoFisher	25-0232
CCD25 Monoclonal Antibody (PC61.5), PE-Cyanine7	ThermoFisher	25-025
Anti-CD40 (T-20), goat	Santa Cruz Biotechnology	sc-1731
CD40 Monoclonal Antibody (1C10), PE	ThermoFisher	12-0401-83
Anti-CD43, mouse, PE	BD Biosciences	553271
CD45R (B220) Monoclonal Antibody (RA3-6B2), PE-Cyanine7	ThermoFisher	25-0452
Anti-CD95 (FAS), mouse, PE	BD Biosciences	554258
Anti-T- and B-Cell Activation Antigen (GL-7), mouse, Alexa Fluor® 647	BD Biosciences	551529
Anti-H2AX (pS139), Mouse, PerCP-Cy™5.5	BD Biosciences	564718
Anti-Histone H3 **(**ChIP Formulated)	Cell Signaling	2650
Goat anti-mouse Ig antibody (ELISA)	Southern Biotech	1010-010
Anti-IgG1, mouse	Cell Signaling	96714
Anti-IgG1, mouse, APC	BD Biosciences	550874
HRP-conjugated goat anti-mouse IgG1 (ELISA)	Southern Biotech	1070-05
HRP-conjugated goat anti-mouse IgG2a (ELISA)	Southern Biotech	1080-05
HRP-conjugated goat anti-mouse IgG2b (ELISA)	Southern Biotech	1090-05
HRP-conjugated goat anti-mouse IgG3 (ELISA)	Southern Biotech	1100-05
HRP-conjugated goat anti-mouse IgA (ELISA)	Southern Biotech	1040-05
Mouse IgD Monoclonal Antibody (11-26c), FITC	ThermoFisher	11-5993
HRP-conjugated goat anti-mouse IgM (ELISA)	Southern Biotech	1021-05
Mouse IgM Monoclonal Antibody (II/41), APC	ThermoFisher	17-5790
Anti-IgM XP®	Cell Signaling	77591
Anti-H3K4me3	Active Motif	39159
Anti-H3K27ac	Active Motif	39034
Anti-H3K27me3	Cell Signaling	9733
Anti-IκBα (C-21), rabbit	Santa Cruz Biotechnology	sc-371
Anti-IκBβ(C-20)	Santa Cruz Biotechnology	sc-945
Anti-IKKγ (FL-419), rabbit	Santa Cruz Biotechnology	sc-8330
Anti-P65 (C20) goat, rabbit	Santa Cruz Biotechnology	sc-372
Anti-phosphorylated IκBα	Cell Signaling	9246
Anti-RelA (D14E12) XP®	Cell Signaling	8242
Anti-RelA, rabbit	Miyamoto et al, 1994	
Anti-RelB (C-19)	Santa Cruz Biotechnology	sc-226
Anti-c-Rel, rabbit	Miyamoto et al, 1994	
Anti-CD90.2 (Thy1.2), mouse, APC	BD Biosciences	553007
Anti-tubulin	EMD Millipore	CP06
Anti-CD4	BD Biosciences	741051
Anti-CD8	BD Biosciences	563786
Db/NP396	NIH Tetramer Core Facility (Emory University, Atlanta, GA)	
I-Ab/GP66	NIH Tetramer Core Facility (Emory University, Atlanta, GA)	
Anti-ICOS	Biolegend	313534
Anti-PD-1	Biolegend	135231
**Oligonucleotides and other sequence-based reagents**		
Nfkb2 F-5’-CAGAACCTGGAGCAACTGCTG-3’ R-5’-ATCTGGGTGGAAGTTGACCG-3’		
CD69 F- 5’-TGGTGAACTGGAACATTGGA-3’ R- 5’-CAGTGGAAGTTTGCCTCACA-3’		
Aicda F- 5’-GAAAGTCACGCTGGAGACCG-3’ R- 5’-TCTCATGCCGTCGCTTGG-3’	Dorsett et al, 2008	
Nbn F- 5’-GGATGGACGCAGAGAGGAAT-3’ R- 5’-CTGGAGGTGGAGTTGTGGTT-3’		
Icam1 F- 5′-CAATTTCTCATGCCGCACAG-3′ R- 5′ AGCTGGAAGATCGAAAGTCCG-3′		
NF-κB probe (5’-CTCAACAGAGGGGACTTTCCGAGAGGCCAT-3’)	Miyamoto et al, 1994	
NBS1 κB #1 probe (5’- GCGGCGGGGAGTTCTTTCCCGGGAGCGGCC-3’)		
NBS1 κB #2 probe (5’- GCGAACCGAGGGGAAGAACCACGCGGAGTT-3’)		
NBS1 κB #3 probe (5’- AGAACCACGCGGAGTTGCCCAGGCGCCCAG-3’)		
NBS1 κB #4 probe (5’- TTTAAAGTTTTCAGAATTTCCCCTCGGGGA -3’)		
unlabeled palindromic κB site (5’-CTCAACAGAGGGGAATTTCCGAGAGGCCAT-3)	Miyamoto et al, 1994	
Igκ-kB mutant sequence (5’-TCAACAGAGCTCACTTTATGAGAGGCC-3’)	Miyamoto et al, 1994	
*Nbn* κB-#2 qPCR primers (for: 5’-CTGACTGGACACGGAAGCTG-3’, rev: 5’- GTGGTTCTTCCCCTCGGTTC-3’)		
*Nbn* κB-#4 qPCR primers (for: 5’- GCAGACCGGAGCGTAGTTAG-3’, rev: 5’-AGTCGCAGGCAAGTGCATAA-3’)		
**Chemicals, enzymes and other reagents**		
Agonistic anti-CD40 clone HM40-3	BD Biosciences	553721
Agonistic anti-IgM	Jackson Immunoresearch Laboratories	115-005-075
Annexin V, PE	Biolegend	640907
Apocynin	MedChem Express	HY-N0088
Apotracker™ Green	Biolegend	427401
Annexin		
2,2′-azino-bis(3-ethylbenzothiazoline-6-sulfonic acid) (ABTS), ELISA	Southern Biotech	0202-01
B Cell Isolation Kit, mouse	Miltenyi	130-090-862
CD4 (L3T4) MicroBeads, mouse	Miltenyi	130-049-201
CD8a(Ly-2) Microbeads mouse	Miltenyi	130-049-401
CD11b(MAC-1) Beads, mouse	Miltenyi	130-049-601
Cycloheximide	Sigma	C7698
Fetal Bovine Serum, Premium Grade	Avantor Seradigm	97068-085
Fixable Viability Dye eFluor™ 450	ThermoFisher	65-0863-14
H2DCFDA	Biotium	10058
IKK16	Selleckchem	S2882
IL-4 (mouse)	Miltenyi	130-097-757
Ionomycin	Sigma	I9657
iScript™ cDNA Synthesis Kit	Bio-Rad	1708891
Lipopolysaccharide (LPS)	Sigma	L2880
Live-or-Dye NucFix	Biotium	32010-T
Lymphocyte Separation Medium	18061	Corning
β-mercaptoethanol	Sigma	M7522
MinElute Kit	Qiagen	28004
MitoSOX™ Mitochondrial Superoxide Indicators	ThermoFisher	M36008
MNase	New England Biolabs	M0247S
N-acetyl-L-cysteine	Sigma	A7250
NEBNext^®^ High-Fidelity 2X PCR Master Mix	New England Biolabs	M0541
NEBNext^®^ Poly(A) mRNA Magnetic Isolation Module	New England Biolabs	E7490
NEBNext^®^ Ultra^™^ II RNA Library Prep Kit for Illumina^®^	New England Biolabs	E7770
NucleoSpin RNA, Mini kit for RNA purification	Macherey-Nagel	740955.50
Penicillin, Streptomycin Solution, 100X	Corning	30-002-CI
Phorbol-12,13-dibutyrate (PDBu)	Sigma	P1269
PIPseq™ T20 3′ Single Cell RNA Kit	Fluent BioSciences	FBS-SCR-T20-4-V3
poly-K63-ubiquitin chains	Boston Biochem	UC-380
poly-SUMO-2 chains	Boston Biochem	ULC-210
Ponceau S staining solution	Bio-Rad	5225
Protein A Magnetic Sepharose beads	Cytivia	28951378
Rotenone	Apexbio Technology LLC	501998711
SENP1	Boston Biochem	E-700
TRIzol	ThermoFisher	15596026
USP2 (human)	Boston Biochem	E-504
ViaFluor 405	Biotium	30068
Zileuton	Ambeed	77667-690
**Software**		
Microsoft Excel	Microsoft	
GraphPad Prism 10	Dotmatics	
FlowJo v10	BD Biosciences	
R	R Project	
GSEA v4.3.2	Broad Institute	
FACSDiva	BD Biosciences	
NIS-Elements	Nikon	
FIJI	ImageJ	
**Other**		
WEHI-231 immature B cells	ATCC	CRL-1702
LSRII	BD Biosciences	
LSR Fortessa	BD Biosciences	
Attune Nxt	Thermofisher	
Ti Eclipse Microscope	Nikon	
Illumina NextSeq 500	Illumina	
Illumina NovaSeq	Illumina	
NovaSeq X Plus	Novogene	

### Generation of NEMO^DK^ and NEMO^S85A^ Mice

The NEMO K270R/K302R (DK) mouse knock-in construct was generated using recombineering techniques (Liu et al, 2003). A bacterial artificial chromosome (BAC) clone containing the murine *Nemo* locus (BAC13D21) was introduced into electrocompetent DY380 cells. A retrieval vector was engineered by subcloning two homology arms derived from the *Nemo* locus into plasmid PL253, consisting of a 490 bp 5′ upstream region (nucleotides 2290–2780) and a 500 bp 3′ region (nucleotides 9620–10,110). The resulting plasmid was linearized with SpeI and electroporated into DY380 cells containing BAC13D21 to mediate gap repair. This process yielded a construct encompassing ~5 kb upstream and 3 kb downstream of the DK mutation sites.

A secondary targeting vector was constructed by amplification and subcloning ~1 kb upstream and 450 bp downstream of the DK mutation sites from BAC13D21 into plasmid PL452. This design inserts a neo cassette floxed by loxP sites between the 5’ and 3’ homology regions of *Nemo*. Site-directed mutagenesis was performed using QuikChange PCR to substitute lysine residues at positions 270 and 302 with arginine (K270: AAA to AGA; K302: AAG to AGG).

The resulting fragment was co-transformed by electroporation into the DY380 bacteria already harboring the gap-repaired PL253 containing the full retrieved genomic *Nemo* gene. The bacteria were then selected on kanamycin plates to select for the correct NEMO^DK^ targeting vector. The resultant vector was confirmed through sequence analysis in its entirety. Subsequently, the finalized targeting vector was linearized and transfected into 129 SV/J ES cells by the University of Wisconsin Carbone Cancer Center (UWCCC) Genome Editing and Animal Models (GEAM) core facility. Correctly targeted ES cell clones were confirmed by Southern blot analyses. The floxed PGK-Neo cassette was removed by infecting positive ES cells with a Cre expression adenovirus. ES cell clones harboring the correct targeting vector were karyotyped and injected into C57BL/6 blastocysts to generate chimeric mice. Integrity of the targeted alteration was confirmed by genomic DNA sequencing.

The NEMO^DK^ line was then backcrossed more than ten times onto C57BL/6 to establish a congenic line. The NEMO gene is X-linked, therefore males harboring the mutant allele are hemizygous. Male WT and female NEMO^DK^ heterozygous littermates were bred, and male WT and NEMO^DK^ mice (typically littermates) were used for all analyses.

Genotyping of mice was done by PCR from DNA generated from ear clips using the following primers: 5’-GG-GCCAAAAGACTCTGACAGCACCCTT-3’, and 5’-GCCCCAGTACAGGGGAACGCCAG-GGCCAT-3’.

Testes were isolated from of WT and NEMO^DK^ littermates and wet weights were measured.

The S85A (serine to alanine) mutation was introduced into exon 4 of NEMO by the UWCCC GEAM core facility. The single-stranded donor oligonucleotide and guide RNA sequences were synthesized by Integrated DNA Technologies (IDT) as follows: 5′-CGCTGTGAGGAGCTGCTGCATTTCCAGGTCGCTCAGCGGGAGGAGAAGGAGTTCCTTATG-3′ and 5′-CCTTGAGGAAGAGGAGGGCGACC-3′, respectively. Guide RNA (50 ng/µL), single-stranded donor DNA (20–50 ng/µL), and Cas9 protein (10–100 ng/µL) were co-microinjected into 12-h C57BL/6 mouse embryos. SCR-7 was included at a final concentration of 1 µM to block the non-homologous end-joining DNA repair pathway. Injected embryos were subsequently implanted into pseudopregnant C57BL/6 females. Integrity of the targeted alteration was confirmed by sequencing of genomic DNA.

Mice were raised in a specific-pathogen-free animal housing facility accredited by the Association for the Assessment and Accreditation of Laboratory Animal Care International. All mice were handled in accordance with animal protocols approved by the Institutional Animal Care and Use Committee of the University of Wisconsin-Madison or the Medical College of Wisconsin Institutional Animal Care and Use Committee.

### Reagents

ATM^−/−^ mice (008536) were purchased from Jackson Laboratories. LPS (L2880), ionomycin (I9657), PDBu (phorbol-12,13-dibutyrate, P1269), Cycloheximide (C7698), and N-acetyl-L-cysteine (A7250) were purchased from Sigma-Aldrich. Agonistic anti-IgM (115-005-075) was purchased from Jackson Immunoresearch Laboratories. Agonistic anti-CD40 clone HM40-3 (553721) was purchased from BD Biosciences. Mouse IL-4 (130-097-757) was purchased from Miltenyi. H_2_DCFDA (10058), ViaFluor 405 (30068), and Live-or-Dye NucFix (32010-T) were purchased from Biotium. Recombinant proteins human USP2 (E-504), SENP1 (E-700), poly-SUMO-2 chains (ULC-210), and poly-K63-ubiquitin chains (UC-380) were purchased from Boston Biochem. Ponceau S staining solution (5225) was purchased from Bio-Rad. Other inhibitors purchased include rotenone (Apexbio Technology LLC, 501998711), zileuton (Ambeed, 77667-690), apocynin (MedChem Express, HY-N0088) and IKK16 (Selleckchem, S2882).

The following antibodies were purchased from Santa Cruz Biotechnology: anti-actin sc-1616; anti-CD40 (T-20) sc-1731; anti-IKKγ (FL-419, 1:1000) sc-8330; anti-IκBα (C-21) sc-371; anti-IκBβ (C-20) sc-945; anti-RelA (C-20) sc-372; anti-RelB (C-19) sc-226. Anti-phosphorylated IκBα #9246, anti-NBS1 #3002, anti-RelA (D14E12) XP® #8242, anti-Mouse IgG1 (96714), and anti-IgM XP® (77591) were purchased from Cell Signaling. Anti-AID (ZA001, 2500) was purchased from ThermoFisher. Anti-tubulin (CP06) and anti-β-actin (A2228, 1:5000) were purchased from Sigma-Aldrich.

The cRel and RelA antibodies were described in (Miyamoto et al, 1994).

### Primary B cell purification

Splenic B cells were purified by negative selection using anti-CD4 (130-049-201), anti-CD8 (130-049-401), and anti-CD11b (130-049-601) conjugated microbeads (Miltenyi Biotech) as previously described (Wuerzberger-Davis et al, 2011). Alternatively, for biochemical analysis, splenic follicular B cells were isolated by negative selection per the manufacturer’s protocol using the mouse B Cell Isolation Kit (Miltenyi Biotech, 130-090-862). B cell purity was measured by staining cells for B220 and analyzed by FACS and was consistently >96%.

### Cell culture

Primary mouse cells and WEHI-231 immature B cells (ATCC, CRL-1702) were cultured at 1–2 × 10^6^ cells/mL and 1–5 × 10^5^ cells/mL, respectively, in RPMI-1640 medium supplemented with 10% (w/v) fetal bovine serum, 50 mM ${{{\rm{\beta }}}}$-mercaptoethanol, and antibiotics (100 IU/mL penicillin and 100 µg/mL streptomycin). Some primary splenic B cells were also cultured with anti-CD40 (2 µg/mL), mouse IL-4 (10 ng/mL), or LPS (20 ng/mL) in the presence or absence of IL-4. When primary splenic B cells were treated with rotenone (0.5 µM), IKK16 (0.5 µM), zileuton (50 µM), or apocynin (40 µM), they were pretreated for 30 min prior to anti-CD40 ( + /− IL-4 stimulation). In experiments using NAC (50 mM), cells were pretreated for 3 h prior to anti-CD40 ( + /− IL-4 stimulation).

### Basal antibody quantification

Ig levels of different isotypes were measured using ELISA. A goat anti-mouse Ig antibody (1010-010) was used as the capture antibody. Detection was performed with HRP-conjugated goat anti-mouse antibodies specific for IgA (1040-05), IgG1 (1070-05), IgG2a (1080-05), IgG2b (1090-05), IgG3 (1100-05), and IgM (1021-05) all at 1:300 dilution. ABTS (0202-01) was used as the HRP substrate. All reagents were purchased from Southern Biotech.

### Flow cytometry

Analysis of single-cell suspensions from bone marrow, thymus and spleen was performed as previously described (Chen et al, 2008). APC-conjugated anti-B220 (17-0452), anti-IgM (17-5790), anti-CD4 (17-0041), PE-Cy7- conjugated anti-B220 (25-0452), anti-CD25 (25-0251), anti-CD23 (25-0232), eF450-conjugated fixable viability dye (65-0863-14), PE-conjugated anti-CD40 (12-0401-83), anti-CD8 (12-0081-83) and FITC-conjugated anti-IgD (11-5993) antibodies were purchased from ThermoFisher. PerCP-Cy5.5-conjugated gH2AX (564718), PE-conjugated anti-CD43 (553271), anti-CD21 (552957), anti-Fas(554258), APC-conjugated anti-Thy1.2 (553007), anti-IgG1 (550874), and Alexa Fluor 647 conjugated anti-GL7 (551529) antibodies were purchased from BD Biosciences. All antibodies were used at a 1:300 dilution. Samples were applied to a flow cytometer (LSRII or LSRFortessa, Becton Dickinson). Data were collected and analyzed using FACSDiva software (Becton Dickinson) or FlowJo software (BD Biosciences).

For Fig. EV2B–D, single cell suspensions of splenocytes were stained with anti-CD4 (1:200, BD Biosciences, 741051), anti-CD8 (1:200, BD Biosciences, 563786), Db/NP396 tetramers (1:150, NIH Tetramer Core Facility), I-Ab/GP66 tetramers (1:150, NIH Tetramer Core Facility), anti-ICOS (1:200, Biolegend, 313534) and anti-PD-1 (1:200, Biolegend, 135231) antibodies using FACS buffer (1% BSA in PBS). Subsequently, cells were fixed in 2% paraformaldehyde and acquired on an LSR-Fortessa flow cytometer. Data was analyzed using the FlowJo software (BD Biosciences).

### LCMV infection and GC B cell analysis

For Figs. 2A–G and  EV2B–E, mice were infected with 2 × 10^5^ plaque-forming units (PFU) of lymphocytic choriomeningitis virus-Armstrong by intraperitoneal injection. Germinal center B cells were identified by flow cytometry based on the expressions of B220, Fas and GL-7. Viral titers in spleen were quantified with a plaque assay using VERO cells, as described previously (Suresh et al, 2005).

### Mixed bone marrow chimeras

µMT mice (Jackson Laboratory) were maintained in the Biological Resource Center at the Medical College of Wisconsin (MCW). Bone marrow cells from NEMO^DK^ or control mice were mixed 1:4 with bone marrow cells from µMT mice (7- or 11-week-old) and transplanted into lethally irradiated (1000 rads) µMT mice by intravenous injection (4 × 10^6^ cells/recipient). Six weeks after transplantation, the recipients were infected with LCMV (Armstrong strain, 2 × 10^5^ pfu/mouse) intraperitoneally as described above. The sera were collected before (day 0) and after infection at the indicated time points. The GC and IgG1^+^ B cells were examined by flow cytometry at day 33 after infection. The levels of LCMV-specific IgG1, IgG2a, and IgG2b in serum were measured by ELISA, using lysates of LCMV-infected BHK cells as plate-bound capture antigens. Detection antibodies were as described in (Chen et al, 2008).

### Proliferation and cell cycle analysis

Cell proliferation and cell cycle were analyzed as previously described (Chen et al, 2008). Briefly, purified splenic B cells (2.5 × 10^4^) were stimulated with anti-IgM (10 μg/mL) or anti-IgM (10 µg/mL) plus IL-4 (10 ng/mL), anti-CD40 (2 μg/mL) or anti-CD40 (2 μg/mL) plus IL-4 (10 ng/mL), or PdBu plus ionomycin for 48 h in a 96-well plate. The cells were then stained with propidium iodide for cell cycle analysis or pulsed for 16 h with ^3^H-thymidine (1 μCi/well). Thymidine-pulsed samples were collected with the use of a MACH III harvester, and the incorporation of ^3^H-thymidine was determined with a Wallac MicroBeta TriLux scintillation system.

Primary splenic B cells were collected in PBS at 1 × 10^6^ cells/mL and incubated with ViaFluor 405 (Biotium, 30068) for 15 min in the dark at 37 °C. An equal volume of cell culture medium was added and incubated for an additional 5 min. Cells were then resuspended in B cell culture media and cultured as described above. Cells were collected at indicated time points and analyzed via a flow cytometer (Thermofisher Attune Nxt). Gating strategy goes as follows: Cells were first gated based on FSC-A and SSC-A to exclude debris and select the main population of intact cells, then singlets were selected, and Viafluor 405^+^ (VL1 detection channel) cells were selected based on unstained cells as a control. Data was analyzed using FlowJo software.

### Live-dead detection assay

Primary splenic B cells via negative selection were collected at the end point after treatment and washed with PBS once and resuspended in PBS at 1 × 10^6^ cells/mL. In total, 1× Live-or-Dye NucFix (Biotium, 32010-T) dead cell stain was added to cells for 30 min and washed once with PBS after. Cells were then fixed in 4% paraformaldehyde for 20 min, washed twice with FACS buffer, and then resuspended in FACS buffer prior to analysis. Cells were analyzed on a flow cytometer (Thermofisher Attune Nxt). Gating strategy goes as follows: Cells were first gated based on FSC-A and SSC-A to exclude debris and select the main population of intact cells, then singlets were selected, and Live or Dye^+^ (RL1 detection channel) cells were selected based on unstained cells as a control. Data was analyzed using FlowJo software.

### Apotracker green and Annexin 5 staining

Primary splenic B cells via negative selection were collected at the end point after treatment and washed with PBS once. Cells were stained with Apotracker™ Green (Biolegend, 427401) per the manufacturer's recommendations and a 1:100 dilution of Annexin V (Biolegend, 640907) and incubated for 15 min at room temperature, protected from light. Cells were collected at indicated time points and analyzed via a flow cytometer (Thermofisher Attune Nxt). Gating strategy goes as follows: Cells were first gated based on FSC-A and SSC-A to exclude debris and select the main population of intact cells, then singlets were selected, and Apotracker Green^+^ (BL1 detection channel) cells were selected, separately from Annexin 5^+^ (BL2 detection channel) cells, based on unstained cells as a control. Data was analyzed using FlowJo software.

### Live cell imaging

Time-lapse bright-field live-cell images were acquired at a single Z-plane every 2 min for a 24-h period using a Plan Apo ×20 air objective (NA = 0.75) on a Nikon Ti Eclipse Microscope equipped with an ORCA-Fusion camera (Hamamatsu), controlled by Nikon NIS-Elements software (version 5.20). Cells were cultured in an 8-well chamber slide (Ibidi, 80807) and maintained at 37 °C in a humid atmosphere with 5% CO_2_ using a stage-top incubator (Tokai Hit, STXG-WSKMX) with temperature feedback control and stimulated for 9 h prior to imaging.

### In vitro class switch recombination assay

Purified splenic B cells were stimulated with anti-CD40 (2 μg/mL) plus IL-4 (10 ng/mL) or LPS (20 ng/mL) plus IL-4 (10 ng/mL). Four days after stimulation, cells were collected and stained with anti-IgG1 (1:300) antibodies and analyzed by flow cytometry.

### RNA-seq analysis of CD40-stimulated B cells

FACS-sorted IgM^low^IgD^+^ mature follicular B cells from WT and NEMO^DK^ mice were left unstimulated or stimulated with anti-CD40 antibodies for 24 h and lysed in TRIzol (ThermoFisher, 15596026) to isolate total RNA. The mRNA was purified using NEBNext^®^ poly(A) mRNA Magnetic Isolate Module (New England Biolabs, E7490). The libraries were constructed using NEBNext^®^ Ultra RNA library Prep kit for Illumina^®^ (New England Biolabs, E7770) and quantified using QubitFluorometer (ThermoFisher) and Kapa Library Quantification Kit (KapaBiosystems). The average size of the libraries was measured using D1000 ScreenTape system (Agilent). A total of 1.7 pmole libraries were sequenced on Illumina NextSeq 500 with NextSeq 500/550 v2 kit. The raw reads of RNA-seq were preprocessed on the Illumina BaseSpace sequence hub and aligned to the mouse reference genome Mus musculus/mm10 (RefSeq) using the aligner STAR. Differentially expressed transcripts with FDR ≤ 0.05 were identified using the DESeq2 program as implemented on the Illumina BaseSpace hub. Functional enrichment and pathway analysis of gene lists was performed using GSEA software (http://software.broadinstitute.org) (Subramanian et al, 2005) with gene signatures from CD40 activated and resting human CD19^+^ B cells (GSE54017) (Shimabukuro-Vornhagen et al, 2014), DAVID Bioinformatics Resources (https://david.ncifcrf.gov/) (Huang et al, 2009a, 2009b), and Reactome pathway knowledgebase analysis (https://reactome.org/) (Fabregat et al, 2018). NF-κB dimer binding motifs and motif scores were identified for the promoters −1000 to +300 relative to the transcription start site of downregulated genes using HOMER (Heinz et al, 2010) and protein binding microarray (Siggers et al, 2012) in which RelA/p50 dimer is from mouse, and cRel/p50 and RelB/p52 dimers are from human promoter regions.

### RNA-seq analysis of CD40 + IL-4 stimulated B cells

Primary splenic B cells isolated by negative selection as described above from WT and NEMO^DK^ mice were treated with anti-CD40 and IL-4 or LPS and IL-4 for 2 days in three biological replications. The frozen pellets were shipped on dry ice to Genewiz/Azenta for RNA sequencing, RNA extraction, sample QC, library preparations, and sequencing reactions. The samples were prepared and analyzed as previously described in (Chang et al, 2023). GSEA was performed using the GSEA software version 4.3.2 from the Broad Institute. Differentially expressed genes were ranked based on log2 fold-change, and enrichment analysis was conducted using the Mouse MSigDB v2024.1.Mm, including the GO M5 gene sets. A total of 1000 permutations were used to estimate statistical significance. Enrichment scores (ES) and normalized enrichment scores (NES) were calculated, and significance was assessed based on false discovery rate (FDR) *q*-values < 0.25, as recommended by the GSEA guidelines.

### Expression quantification, differential gene expression and mining algorithm for genetic controllers (MAGIC)

Reads were mapped to the mm39 build mouse genome and subjected to Likelihood Ratio Tests using Deseq2 (Love et al, 2014). Genes were classified as expressed if they had at least three reads in all samples of any one of the six conditions. This gave 21,119 genes. Deseq2 output was then filtered for only expressed genes and adjusted *P* values were recalculated for the reduced number of genes. Genes with an adjusted *P* < 0.1 defined as differentially expressed (7648 genes) were subjected to Leiden clustering with resolution = 1.0 using the scanpy Leiden tool (REF: PMID 29409532). The Leiden algorithm clusters genes based on how similarly they are expressed across different conditions. Genes within a cluster show similar behavior across the 6 conditions. This clustering helps to identify groups of genes that may be functionally related in experiments with more than 2 conditions.

Purple, orange and aqua cluster genes were passed through MAGIC using default settings (Roopra, 2020) but with *r* = 1 and GTFs were ignored. GTFs were defined as any ENCODE Factors containing the strings “TBP”, “POL”, “GTF”, or “TAF”. Predicted transcription factors and coregulators were ordered by Score and those with *P*adj≤0.05 were graphed.

### ATAC-seq analysis

ATAC-seq was performed as previously described (Buenrostro et al, 2013). Briefly, 50,000 splenic B cells were isolated from WT and NEMO^DK^ mice and stimulated with anti-CD40 and IL-4 for two days. Cells were washed once with cold PBS, lysed in cold lysis buffer, and immediately centrifuged to collect nuclei. The nuclei pellet was resuspended in a transposition reaction mix (Illumina) and incubated at 37 °C for 30 min. Following transposition, DNA was purified using the MinElute Kit (Qiagen, 28004) and amplified with NEBNext^®^ PCR Master Mix (New England Biolabs, M0541). Libraries were sequenced on an Illumina NovaSeq platform to generate paired-end 150 bp reads.

Raw reads were pre-processed, aligned, and subjected to peak calling for data analysis using the nf-core/atacseq pipeline (https://doi.org/10.5281/zenodo.2634132). Data normalization and differential peak analysis were performed using DESeq2 v1.32.0 (Love et al, 2014). Transcription factor motif analysis and regulatory network inference were conducted using HOMER (Heinz et al, 2010) and TRRUST (Han et al, 2015), respectively.

### CUT&Tag analysis

CUT&Tag was performed as previously described (Kaya-Okur et al, 2020). Briefly, splenic B cells were isolated from WT and NEMO^DK^ mice, stimulated with anti-CD40 and IL-4 for two days, then washed and resuspended in wash buffer. Cells were bound to ConA-coated beads, incubated overnight with anti-RelA (Cell Signaling, 8242), anti-H3K4me3 (Active Motif, 39159), anti-H3K27ac (Active Motif, 39034), and anti-H3K27me3 (Cell Signaling, 9733) antibodies, followed by secondary incubation with anti-rabbit IgG antibodies (Antibodies Online). All antibodies were used at a 1:50 dilution. After washing, the cells were incubated with the pA-Tn5 adapter complex (Epicypher) at room temperature for 1 h. Tagmentation was performed at 37 °C, after which DNA was extracted and PCR-amplified to generate sequencing libraries. Libraries were sequenced on an Illumina NovaSeq platform to produce paired-end 150 bp reads.

For data analysis, reads were aligned to the mm10 mouse genome, and peaks were identified using SEACR v1.3 (Meers et al, 2019) in stringent mode with IgG controls. Data normalization and differential peak analysis were conducted using DESeq2 v1.32.0 (Love et al, 2014). Heatmaps and visualizations were generated using Complex Heatmap v2.8.0 (Gu et al, 2016).

### qRT-PCR analysis

RNA was isolated using NucleoSpin RNA mini kit for RNA purification (Macherey-Nagel, 740955.50) and reverse transcribed using iScript™ cDNA synthesis kit (Bio-Rad, 1708891) according to the manufacturer’s protocol. qPCR was performed using and acquired on a Bio-Rad CFX96. Each reaction was run in triplicate. Primers used: *NfκB2* (for: 5’-CAGAACCTGGAGCAACTGCTG-3’, rev: 5’-ATCTGGGTGGAAGTTGACCG-3’), *CD69* (for: 5’-TGGTGAACTGGAACATTGGA-3’, rev: 5’-CAGTGGAAGTTTGCCTCACA-3’), *Aicda* (for: 5’-GAAAGTCACGCTGGAGACCG-3’, rev: 5’-TCTCATGCCGTCGCTTGG-3’) (Dorsett et al, 2008), *Nbn* (for: 5’-GGATGGACGCAGAGAGGAAT-3’, rev: 5’-CTGGAGGTGGAGTTGTGGTT-3’), *Icam1* (for: 5′-CAATTTCTCATGCCGCACAG-3′, rev: 5′ AGCTGGAAGATCGAAAGTCCG-3′).

### Single cell-RNA sequencing preprocessing and analysis

Splenic B cells were isolated from WT and NEMO^DK^ mice, either left unstimulated or stimulated with anti-CD40 and IL-4 for three days. On day three, cells were collected and subjected to density gradient centrifugation using Lymphocyte Separation Medium (Corning 25-072-CI) to remove dead cells and debris. In all, 40,000 cells were used to make single-cell RNA-seq libraries using the PIPseq™ T20 3′ Single Cell RNA Kit (Fluent BioSciences, FBS-SCR-T20-4-V3) according to the manufacturer’s instructions. Libraries were sequenced by Novogene using lane-based sequencing on a NovaSeq X Plus platform (25B flow cell, paired-end 150 bp configuration).

Pre-processing was done using the PIP-Seeker analysis pipeline v02.00.00 (https://www.fluentbio.com/products/pipseeker-software-for-data-analysis/) and we use recommended sensitivity_5 option, resulting in ~15,000 cells for the samples without stimulation and ~20,000 cells with CD40 stimulation, after removing potential doublets (2–5%) using DoubletFinder and further keeping only the cells with <20% mitochondria content for downstream analysis, including normalization, high dimensional deduction using 5000 genes as number of variable features and 30 principal components for downstream analysis using Seurat package (Stuart et al, 2019; McGinnis et al, 2019) (McGinnis et al, 2019; Stuart et al, 2019). We used Harmony for integration analysis across all 4 samples. Graph-based Seurat clustering was used with resolution 0.2, resulting in 10 clusters (Korsunsky et al, 2019). B cell cluster annotation using differentially expressed genes based on existing B cell subset signatures, with two activated B cell subsets merging into one subset (Verstegen et al, 2023). UMAP (Uniform Manifold Approximation and Projection) and violin plots of representative gene signatures were done using Seurat. Analysis and other barplot functions were performed in R.

### EMSA and immunoblotting analyses

Whole cell extracts were made using total extract (TOTEX) buffer (20 mM HEPES (pH 7.9), 350 mM NaCl, 1 mM MgCl_2_, 0.5 mM EDTA, 0.1 mM EGTA, 0.5 mM DTT, 20% glycerol, and 1% NP-40). EMSA and immunoblots were performed as described previously (Miyamoto et al, 1994). EMSA was performed using a double-stranded and ^32^P end-labeled NF-κB probe (5’-CTCAACAGAGGGGACTTTCCGAGAGGCCAT-3’), NBS1 κB #1 probe (5’- GCGGCGGGGAGTTCTTTCCCGGGAGCGGCC-3’), NBS1 κB #2 probe (5’- GCGAACCGAGGGGAAGAACCACGCGGAGTT-3’), NBS1 κB #3 probe (5’- AGAACCACGCGGAGTTGCCCAGGCGCCCAG-3’), and NBS1 κB #4 probe (5’- TTTAAAGTTTTCAGAATTTCCCCTCGGGGA-3’).

For Supershift Analysis, 1.5 µl of anti-cRel or anti-p65 antibody (Santa Cruz, sc-372) was added to the binding reaction and incubated for 20 min prior to addition of the Igκ-κB probe (Miyamoto et al, 1994). For cold-probe competition assays 20-fold excess of unlabeled probe was added to the binding reaction and incubated for 20 min before probe addition. The sequence for the unlabeled palindromic κB site is 5’-CTCAACAGAGGGGAATTTCCGAGAGGCCAT-3 and Igκ-κB mutant sequence (5’-TCAACAGAGCTCACTTTATGAGAGGCC-3’) (Miyamoto et al, 1994). Band intensities from EMSA were quantified using ImageQuant software according to the manufacturer’s instructions (GE Healthcare).

For the detection of monoubiquitinated NEMO, 200 µg of total cell extracts were used. If smaller amounts were used, the detection of the modified NEMO bands became less efficient. There was no need to add any deubiquitinase inhibitors, such as N-ethylmaleimide or iodoacetamide, to detect the monoubiquitinated NEMO, unlike sumoylated NEMO. Immunoblot signals were quantified using ImageJ.

### DCFDA ROS and mitochondrial ROS detection assay

Primary splenic B cells via negative selection were collected at end point after treatment and resuspended in cell media at 1 × 10^6^ cells/mL and incubated with 20 mM DCFDA for 15 min in the dark at 37 °C. An equal volume of cell media was added and incubated for an additional 5 min. Cells were then centrifuged and washed twice with PBS. Prior to analysis cells were resuspended in phenol-red-free media. Similarly, to measure mitochondrial ROS, cells were incubated with 1 mM MitoSOX (Invitrogen, M36008) for 30 min at 37 °C, washed three times with warm PBS. Cells were analyzed on a flow cytometer (Thermofisher Attune Nxt). Data was analyzed using FlowJo software.

### In vitro deconjugating assay

WEHI-231 cell extracts (200 µg) were incubated with and without 4 µM recombinant human USP2 deubiquitinase or human SENP1 SUMO protease for 1 h in TOTEX buffer and analyzed by SDS-PAGE and immunoblot.

### ChIP-qPCR analyses

Cells at a concentration of 1 × 10^6^ cells/mL were crosslinked with 0.5% formaldehyde for 5 min, quenched with 125 mM glycine for 5 min, and washed once with PBS. Cells were lysed (50 mM HEPES pH 7.9, 0.5% NP-40, 0.25% Triton X-100) for 15 min at room temperature, rotating. Cells are washed three times with MNase digestion buffer (50 mM HEPES pH 7.9, 10 mM CaCl_2_, 150 mM NaCl). In MNase digestion buffer, cells were sonicated with a Sonic Dismembrator (Fisher Scientific) seven times, 30 s each round. MNase (New England Biolabs, M0247S, 1:200) was added for 30 min, shaking at 500 RPM at 37 °C using a thermomixer (Eppendorf). MNase was quenched with 10X Quenching Buffer (100 mM EDTA, 10 mM EGTA, 1.5 M NaCl) with 0.1% SDS. Cells were further sonicated twice for 30 s each round. Samples were then incubated with 5X volume of IP buffer (20 mM Tris-Cl pH 7.5, 150 mM NaCl, 3 mM EDTA, 3 mM EGTA, 0.5% NP-40, and protease inhibitors) with either RelA (1:200), Histone 3 (Cell Signaling, 2650; 1:50), or IgG1 isotype control (Cell Signaling, 3900; 1:50) antibodies overnight at 4 °C, rotating (Miyamoto et al, 1994). Samples are then incubated with equilibrated Protein A Magnetic Sepharose beads (Cytivia, 28951378, 1:120) for 5 h at 4 °C, rotating. Beads were then washed with IP buffer (3×), IP buffer + 0.3 M NaCl (2×), LiCl buffer (2×, 0.25 M LiCl, 0.5% NP-40, 0.5% Na-Deoxycholate), and TE buffer (1×, 10 mM Tris-HCl pH 8, 1 mM EDTA, 50 mM NaCl). Beads were then incubated in EB buffer (50 mM Tris-HCl pH 8, 10 mM EDTA, 1% SDS) with proteinase K to de-crosslink overnight at 65 °C shaking at 700 rpm. Beads were then incubated with RNase A for 30 min at 37 °C. DNA was then recovered by PCR purification kit (28104, Qiagen).

qPCR was performed using and acquired on a Bio-Rad CFX96. Primers used: *Nbn* κB-#2 (for: 5’-CTGACTGGACACGGAAGCTG-3’, rev: 5’-GTGGTTCTTCCCCTCGGTTC-3’) and *Nbn* κB-#4 (for: 5’-GCAGACCGGAGCGTAGTTAG-3’, rev: 5’-AGTCGCAGGCAAGTGCATAA-3’).

### Experimental studies and statistical analysis

Investigators were aware of mouse genotypes during experiments, and no blinding was performed. Mice were included based on availability and were not selected according to specific experimental criteria, except for age. Only male mice were used due to the availability of appropriately matched WT littermates. For biochemical assays, data points were excluded during initial protocol optimization; however, once experimental conditions were established, all data from subsequent experiments were included. Data were excluded only in cases of clear technical failure, such as excessive background in immunoblots that precluded reliable quantification. All “*n*” numbers described in figure legends represents biological replicates.

Statistical tests were selected as appropriate for each dataset, with assumptions of normality and homogeneity of variance assessed prior to analysis; variation within groups was considered when performing statistical analyses, although not explicitly reported. Statistical analysis was done using the GraphPad Prism 10 Software (GraphPad Software Inc., La Jolla, CA, USA). CUT&TAG and ATAC-Seq were pre-processed with FASTQC, trimmed for adapters, and aligned with the mouse genome mm10 using Bowtie2. Peaks were called with MACS2 (ATAC-Seq) and SEACR (CUT&TAG). Differential accessibility was assessed with DESeq2 using Wald tests. GSEA *p*-values were adjusted via the Benjamini–Hochberg method. Transcription factor networks were predicted using TRRUST with Fisher’s exact test (Han et al, 2018), and motif enrichment was analyzed with HOMER.

## Supplementary information


Appendix
Peer Review File
Source data Fig. 1
Source data Fig. 2
Source data Fig. 3
Source data Fig. 4
Source data Fig. 6
Source data Fig. 7
Source data Fig. 8
Source data Fig. 9
Expanded View Figures


## Data Availability

Datasets generated in this study have been deposited to public repositories as follows: -Bulk RNA-seq: GEO accession GSE325803; Single-cell RNA-seq: GEO accession GSE325910; ATAC-seq: GEO accession GSE325579; CUT&Tag data: GEO accession GSE325580. The source data of this paper are collected in the following database record: biostudies:S-SCDT-10_1038-S44318-026-00875-0.
