# Supplementary material for: NF-κB/RelA signaling required for CD40-induced humoral immunity depends on specific NEMO lysine residues in mice
Source: EMBO J. 2026 Jul 30;45(17):6229–66. doi: 10.1038/s44318-026-00875-0 (PMC13534677; doi:10.1038/s44318-026-00875-0)
Supplement: Supplementary file 1 [file 44318_2026_875_MOESM11_ESM.pdf]

Expanded View Figures

Figure EV1. NEMO^{DK} mice display normal lymphocyte development and maturation.

(**A**) Bone marrow cells from WT and NEMO^{DK} mice were stained with anti-B220 and anti-IgM. Percentages shown by each box indicate cells in the gated lymphoid population. Flow cytometry plots of pro-/pre- (B220^{lo}IgM^{-}), immature (B220^{lo}IgM^{+}), and recirculating mature (B220^{hi}IgM^{+}) B cells shown are representative of five mice per genotype and displayed graphically with mean and SD. (**B**) Flow cytometry plots of splenic B (B220^{+}) and T cells (Thy1.2^{+}) is displayed graphically with mean and SD ($n = 5$). (**C**) Splenocytes were stained with anti-B220, anti-IgM, and anti-IgD. For cells gated on B220^{+}, flow plots of T1 (IgM^{hi}IgD^{-}), T2 (IgM^{hi}IgD^{+}), and FO (IgM^{lo}IgD^{+}) B cells are shown and displayed graphically with mean and SD ($n = 5$). (**D**) Splenocytes were stained with anti-B220, anti-CD21, and anti-CD23. For cells gated on B220^{+}, flow plots of MZ B cells (CD21^{hi}CD23^{lo}) are shown and graphically displayed with mean and SD ($n = 5$). (**E**) Thymocytes from WT and NEMO^{DK} mice were stained with anti-CD4 and anti-CD8 antibodies. Data is represented as bar graphs of the total number of cells with mean and SD ($n = 5$/genotype). (**F**) Cells from (**E**) represented bar graphs of the total number of double negative (DN), CD4^{+}, CD8^{+}, and double positive (DP) T cells with mean and SD ($n = 5$/genotype). Data information: statistical analysis was performed using unpaired two-tailed Welch's *t* test to compare WT and NEMO^{DK} samples. *P* values are indicated: $P \leq 0.05$.

 © The Author(s)

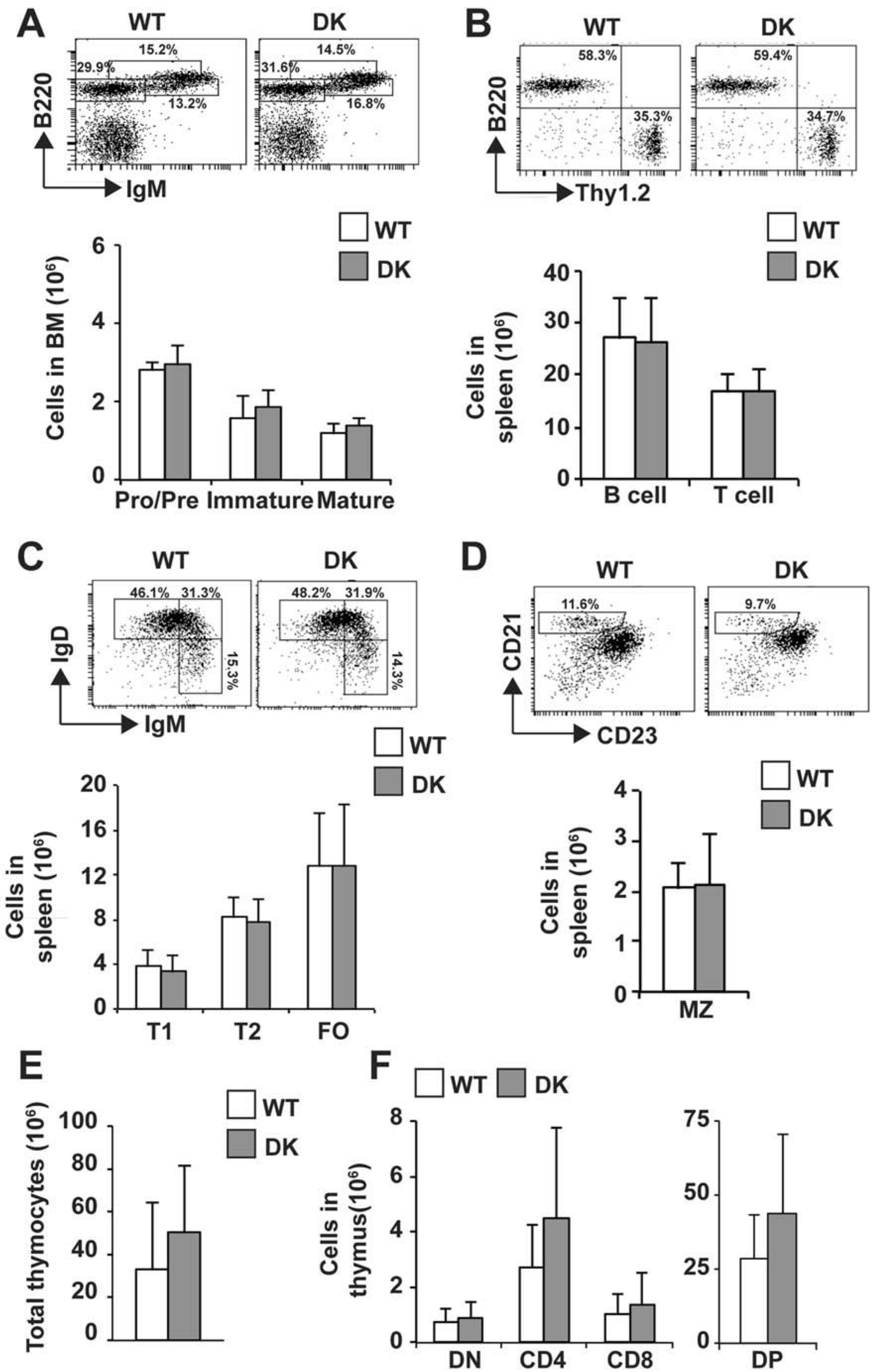
A
WT
DK
15.2%
29.9%
13.2%
14.5%
31.6%
16.8%
B220
IgM
WT
DK
Cells in BM (10^6)
Pro/Pre Immature Mature
B
WT
DK
58.3%
35.3%
59.4%
34.7%
B220
Thy1.2
WT
DK
Cells in spleen (10^6)
B cell T cell
C
WT
DK
46.1% 31.3%
15.3%
48.2% 31.9%
14.3%
IgD
IgM
WT
DK
Cells in spleen (10^6)
T1 T2 FO
D
WT
DK
11.6%
9.7%
CD21
CD23
WT
DK
Cells in spleen (10^6)
MZ
E
WT
DK
Total thymocytes (10^6)
F
WT DK
Cells in thymus(10^6)
DN CD4 CD8
DP

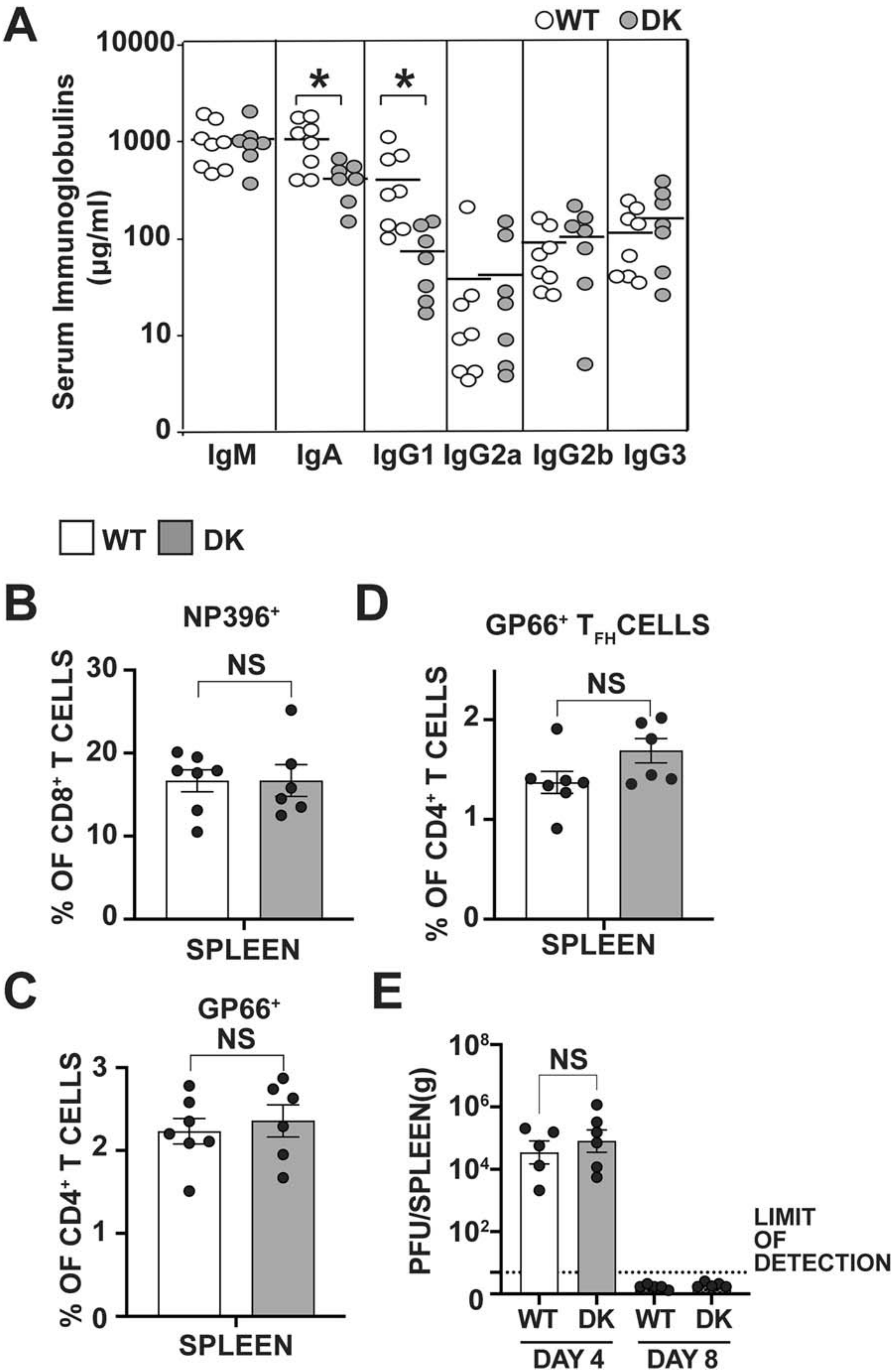
A
OWT ODK
10000
1000
100
10
0
Serum Immunoglobulins (µg/ml)
*
*
IgM IgA IgG1 IgG2a IgG2b IgG3
WT DK
B
NP396⁺
NS
30
20
10
0
% OF CD8⁺ T CELLS
SPLEEN
D
GP66⁺ T FH CELLS
NS
2
1
0
% OF CD4⁺ T CELLS
SPLEEN
C
GP66⁺
NS
3
2
1
0
% OF CD4⁺ T CELLS
SPLEEN
E
NS
10⁸
10⁶
10⁴
10²
0
PFU/SPLEEN(g)
LIMIT OF DETECTION
WT DK WT DK
DAY 4 DAY 8

 © The Author(s)

Figure EV2. NEMO^{DK} mice exhibit decreased basal antibody levels but normal T cell responses to lymphocytic choriomeningitis virus.

(**A**) Basal amounts of indicated serum Ig subtypes were determined by ELISA. Data was obtained from WT ($n = 8$) and NEMO^{DK} ($n = 7$) mice. Cohorts of WT and NEMO^{DK} mice were infected with LCMV-Armstrong. On the 8th day after infection, T cell responses were assessed in the spleen. Single cell suspensions of splenocytes were stained with anti-CD4, anti-CD8, D^b/NP396 tetramers, I-A^b/GP66 tetramers, anti-ICOS and anti-PD-1 antibodies. (**B**) Graphical representation of the percentages of NP396-specific cells among CD8$^+$ T cells with mean and SD, $P = 0.9853$ (WT $n = 7$, DK $n = 6$). (**C**) Graphical representation of the percentages of GP66-specific cells among CD4$^+$ T cells with mean and SD, $P = 0.6136$ (WT $n = 7$, DK $n = 6$). (**D**) Graphical representation of the percentages T_{FH} cells among GP66-specific CD4$^+$ T_{FH} cells with mean and SD, $P = 0.08$ (WT $n = 7$, DK $n = 6$). (**E**) Graphical representation of LCMV titers in spleen at day 4 and 8 after infection with mean and SD (WT $n = 7$, DK $n = 6$). Data information: statistical analysis was performed using unpaired Student's *t* test to compare WT and NEMO^{DK} samples. *P* values are indicated: ns, not significant; "*", significant with $P \leq 0.05$.

© The Author(s)

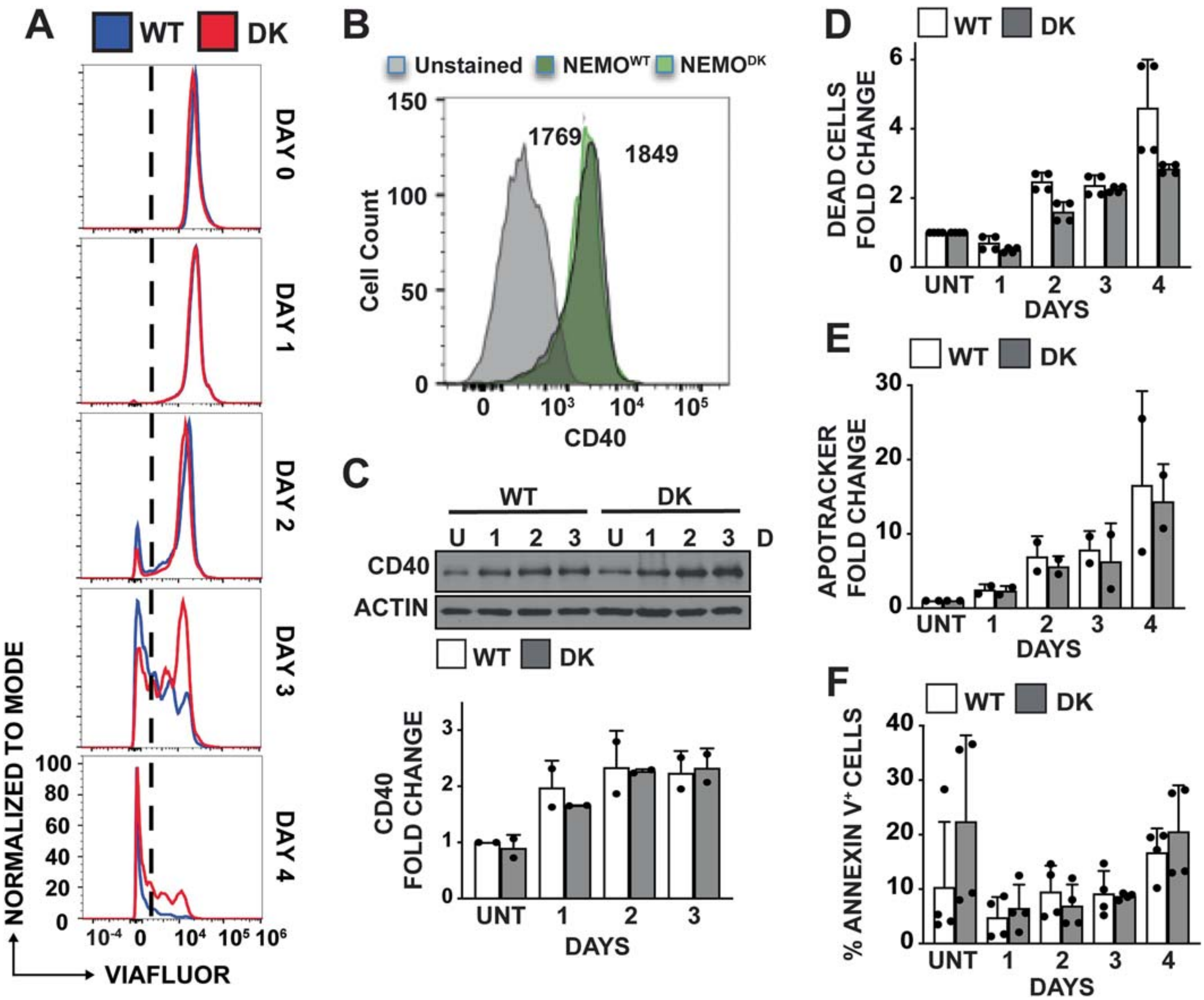

Figure EV3. CD40-specific defects in NEMO^DK B cells are not due to different CD40 expression or increased cell death.

(**A**) WT and NEMO^DK B cells were stimulated with CD40 + IL-4 and stained with Viafluor 405 to track proliferation over 4 days and analyzed by FACS. FACS plots are represented as histograms, the dotted line denotes the population of cells that have divided (left) and not divided (right). (**B**) Splenic B cells were stained for CD40 and analyzed by flow cytometry. Representative MFI are displayed ($n = 5$). (**C**) Lysates from WT and NEMO^DK B cells were stimulated with CD40 + IL-4 over the course of 3 days and analyzed by immunoblot for CD40 protein and actin as loading control. Data is represented graphically as CD40 expression normalized to actin with relative fold change compared to WT untreated control with mean and SD ($n = 2$). (D) Quantification of dead cells measured by Live-or-Dye Nucfix™ Red viability stain of WT and NEMO^DK B cells stimulated with CD40 + IL-4. Data represented as relative fold change compared to WT untreated control with mean and SD ($n = 4$). (**E**) Cells treated as described in (**D**) were stained with Apotracker Green and quantified with data represented as in (**D**) ($n = 2$). (**F**) WT and NEMO^DK B cells were treated as described in (**D**), stained with Annexin V and quantified. Data is represented as the mean percentage of annexin 5⁺ cells with SD ($n = 4$). Data information: statistical analysis was performed using unpaired two-tailed Welch's *t* test to compare WT and NEMO^DK samples.

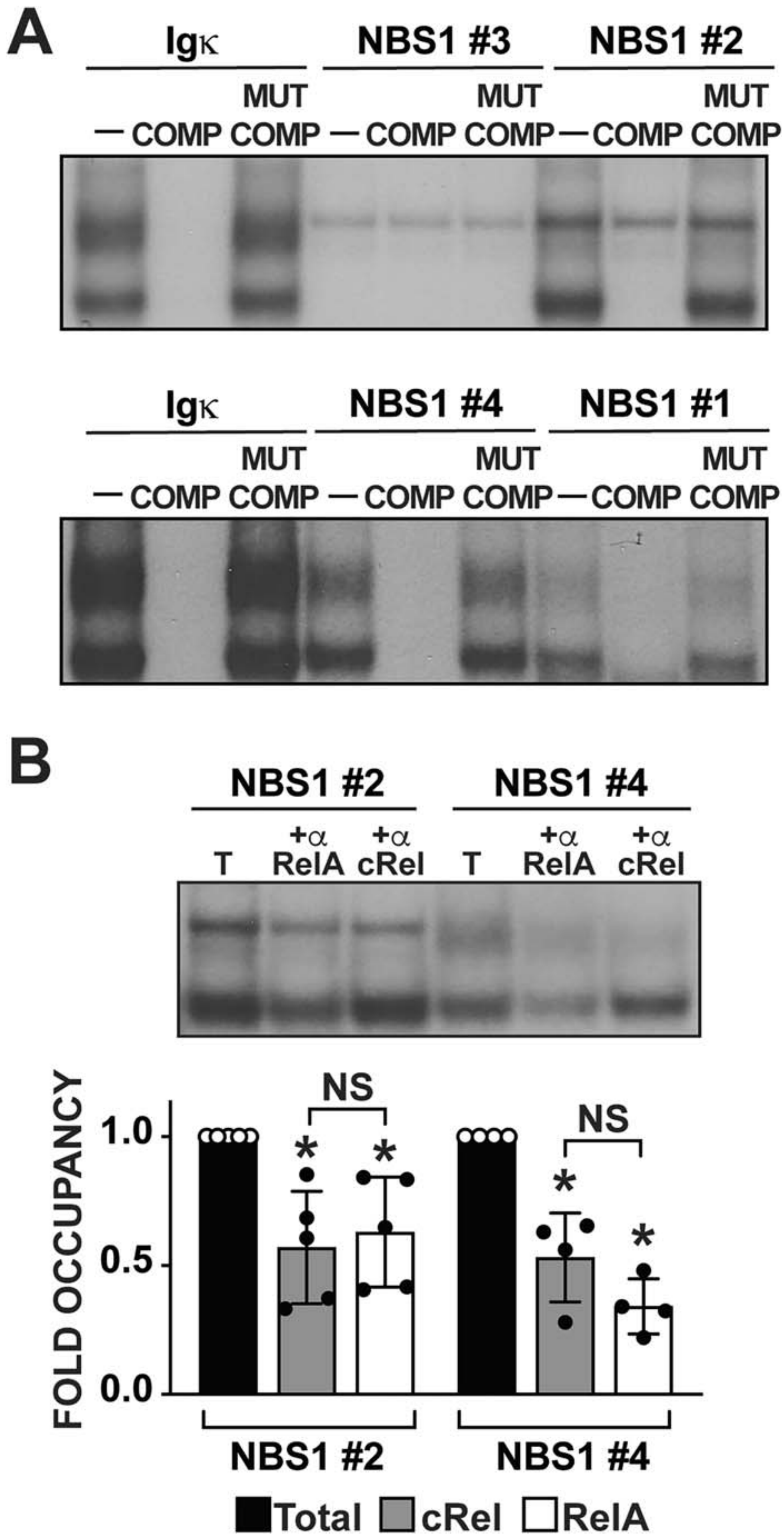
A
Igκ
NBS1 #3
NBS1 #2
— COMP MUT COMP
— COMP MUT COMP
— COMP MUT COMP
Igκ
NBS1 #4
NBS1 #1
— COMP MUT COMP
— COMP MUT COMP
— COMP MUT COMP
B
NBS1 #2
NBS1 #4
T +α RelA +α cRel
T +α RelA +α cRel
FOLD OCCUPANCY
1.0
0.5
0.0
NS
NS
NBS1 #2
NBS1 #4
Total cRel RelA

Figure EV4. Binding specificity of putative κB sites within Nbn locus proximal to start site.

(**A**) Competitive binding assay with samples from (Fig. 6J) of the 4 *Nbn* putative κB sites and Igκ-κB with unlabeled 20-fold excess duplexed oligos of authentic WT and mutated κB sites. (**B**) Supershift analysis of the samples from (Fig. 6J) incubated with either RelA or cRel antibodies to determine binding to NBS1 κB-#2 and -#4. Quantification of the remaining bands (leftover NF-κB subunit after shifting) is represented as the relative fold change compared to the "total" sample which contains both RelA and cRel with mean and SD ($n = 5$ for κB-#2, $n = 4$ for κB-#4). For κB-#2, cRel $P = 0.004$ and RelA $P = 0.01$. For κB-#4, cRel $P = 0.0006$ and RelA $P = 6.74 \times 10^{-5}$. Data information: statistical analysis was performed using unpaired two-tailed Welch's *t* test to compare cRel and RelA samples and ordinary one-way ANOVA with multiple comparisons to the total samples. *P* values are indicated: "*" indicates statistically significant.

 © The Author(s)

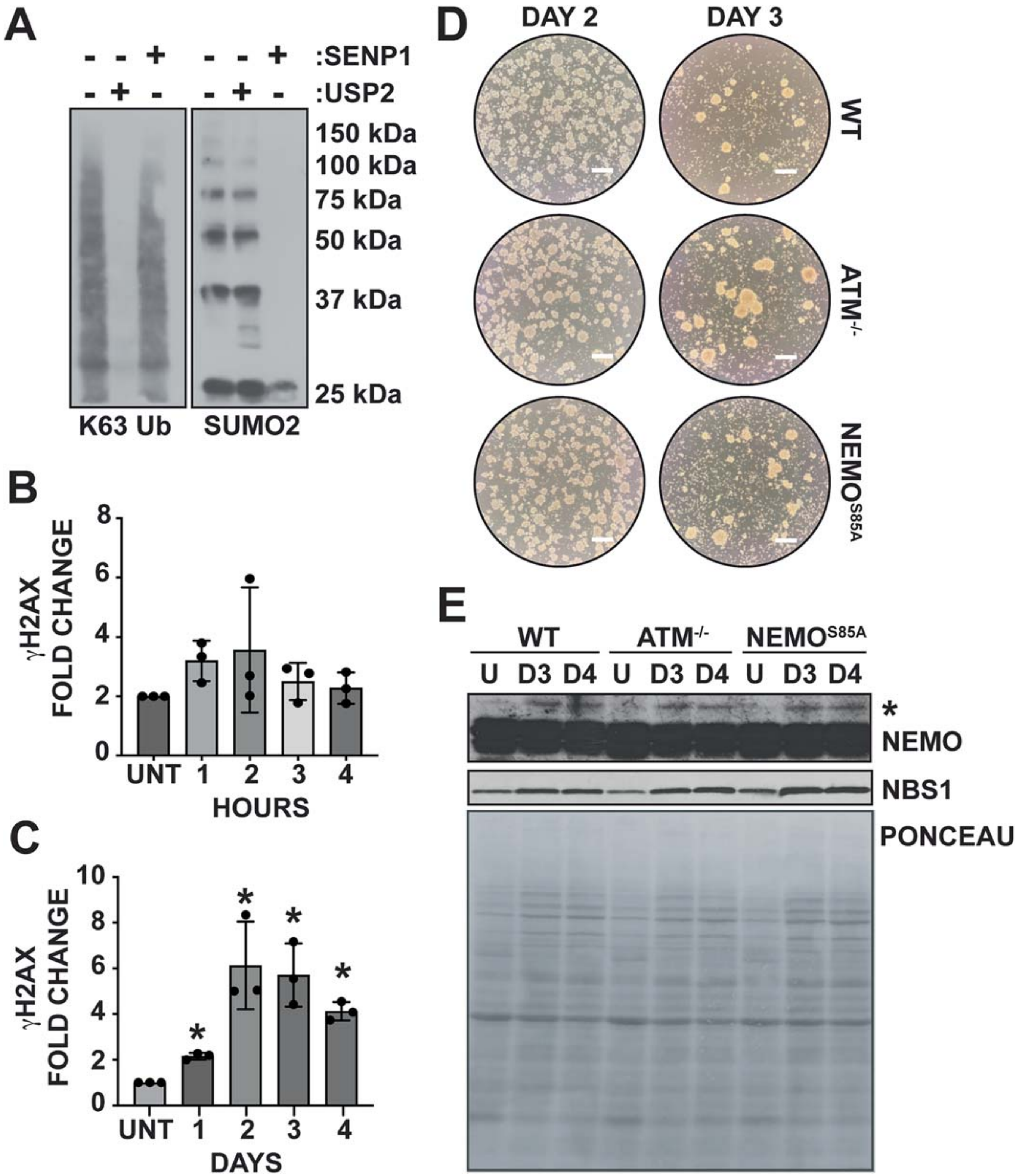
A
- - + :SENP1
- + - - + - :USP2
- - +
150 kDa
100 kDa
75 kDa
50 kDa
37 kDa
25 kDa
K63 Ub
SUMO2
B
γH2AX FOLD CHANGE
UNT 1 2 3 4
HOURS
C
γH2AX FOLD CHANGE
UNT 1 2 3 4
DAYS
D
DAY 2
DAY 3
WT
ATM-/-
NEMOS85A
E
WT
ATM-/-
NEMOS85A
U D3 D4 U D3 D4 U D3 D4
*
NEMO
NBS1
PONCEAU

Figure EV5. CD40-induced responses are independent of DNA damage-ATM-NEMO signaling.

(**A**) Recombinant K63-linked polyubiquitin or SUMO-2 chains were incubated with USP2 or SENP1 and analyzed by immunoblot with anti-K63 Ub or anti-SUMO2 antibodies ($n = 2$). (**B**) WT primary splenic B cells were stimulated with CD40 + IL-4 over the course of 4 h, stained for γH2AX, and analyzed by flow cytometry. Data is represented as the MFI fold change for each condition normalized to the untreated control, and values presented as bar graphs showing the mean relative fold change and SD ($n = 3$). (**C**) WT primary splenic B cells were stimulated with CD40 + IL-4 over the course of 4 days and analyzed as in (**B**) ($n = 3$, day 1 $P = 0.005$, day 2 $P = 0.04$, day 3 $P = 0.03$, day 4 $P = 0.006$). (**D**) Representative images of HA from WT ($n = 3$ pooled), $ATM^{-/-}$ ($n = 3$ pooled) and $NEMO^{S85A}$ ($n = 3$ pooled) stimulated with CD40 + IL-4 for 4 days. HA images from days 2 and 3 are displayed, scale bar on bottom right represents 100 µm ($n = 1$). (**E**) Lysates from day 3 and 4 samples from (**D**) were analyzed for NEMO and NBS1, with ponceau staining for loading control. The asterisk denotes monoubiquitinated NEMO ($n = 1$/genotype). Data information: statistical analysis was performed using ordinary one-way ANOVA with multiple comparisons to the untreated condition. "*" indicates statistically significant.

 © The Author(s)
