# Supplementary material for: NF-κB/RelA signaling required for CD40-induced humoral immunity depends on specific NEMO lysine residues in mice
Source: EMBO J. 2026 Jul 30;45(17):6229–66. doi: 10.1038/s44318-026-00875-0 (PMC13534677; doi:10.1038/s44318-026-00875-0)
Supplement: Supplementary file 2 [file 44318_2026_875_MOESM1_ESM.pdf]

Appendix for:

NF-κB/RelA signaling required for CD40-induced humoral immunity depends on specific NEMO lysine residues in mice

Table of Contents:

Appendix Figure S1	3-4
Appendix Figure S2	5-6
Appendix Figure S3	7-8
Appendix Figure S4	9-10
Appendix Figure S5	11-12
Appendix Figure S6	13-14

APPENDIX FIGURE S1

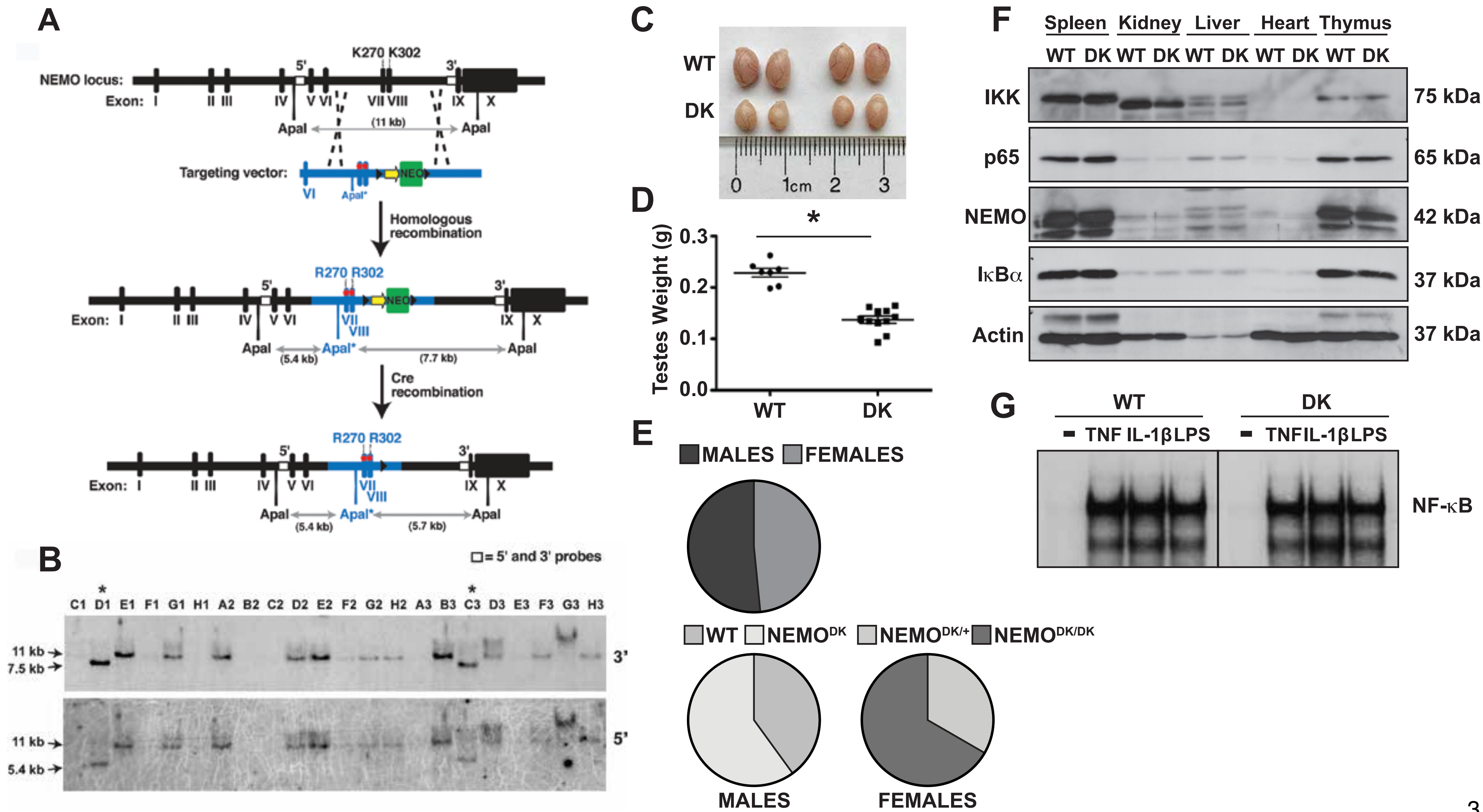

Appendix Figure S1. Generation and characterization of the NEMO^{DK} mouse model. (A) Schematic of the targeting construct used to generate NEMO^{DK} genetically engineered mouse model. Black boxes represent *Nemo* coding exons. 5' and 3' probes used for Southern blotting are shown as white boxes. Black triangles represent lox P sites flanking a PGK-Neo cassette. Cre-mediated deletion of floxed PGK-Neo generated the NEMO^{DK} allele. **(B)** Correctly targeted ES cell clones, denoted by asterisks, were verified by Southern blot analysis using a 3' or 5' probe. **(C)** Photo of WT and NEMO^{DK} testes. **(D)** Quantification of WT (n=7) and NEMO^{DK} (n=11) testis weight. **(E)** Graphical representation of the outcomes of the offspring from the mating of hemizygous male and heterozygous female NEMO^{DK} mutant mice, separated by sex (males n=40, females n=36). **(F)** Immunoblot analysis of NF-κB signaling components in spleen, kidney, liver, heart, and thymus from WT and NEMO^{DK} mice (n=1). **(G)** WT and NEMO^{DK} MEFs were treated with TNFα (10ng/mL) for 20 minutes, IL-1β (10ng/mL) for 1 hour, or LPS (10μg/mL) for 1 hour and analyzed for NF-κB activation by EMSA (n=2).

Data information: statistical analysis was performed using unpaired Student's *t*-test to compare WT and NEMO^{DK} samples. *P* values are indicated: $p \leq 0.05$.

APPENDIX FIGURE S2

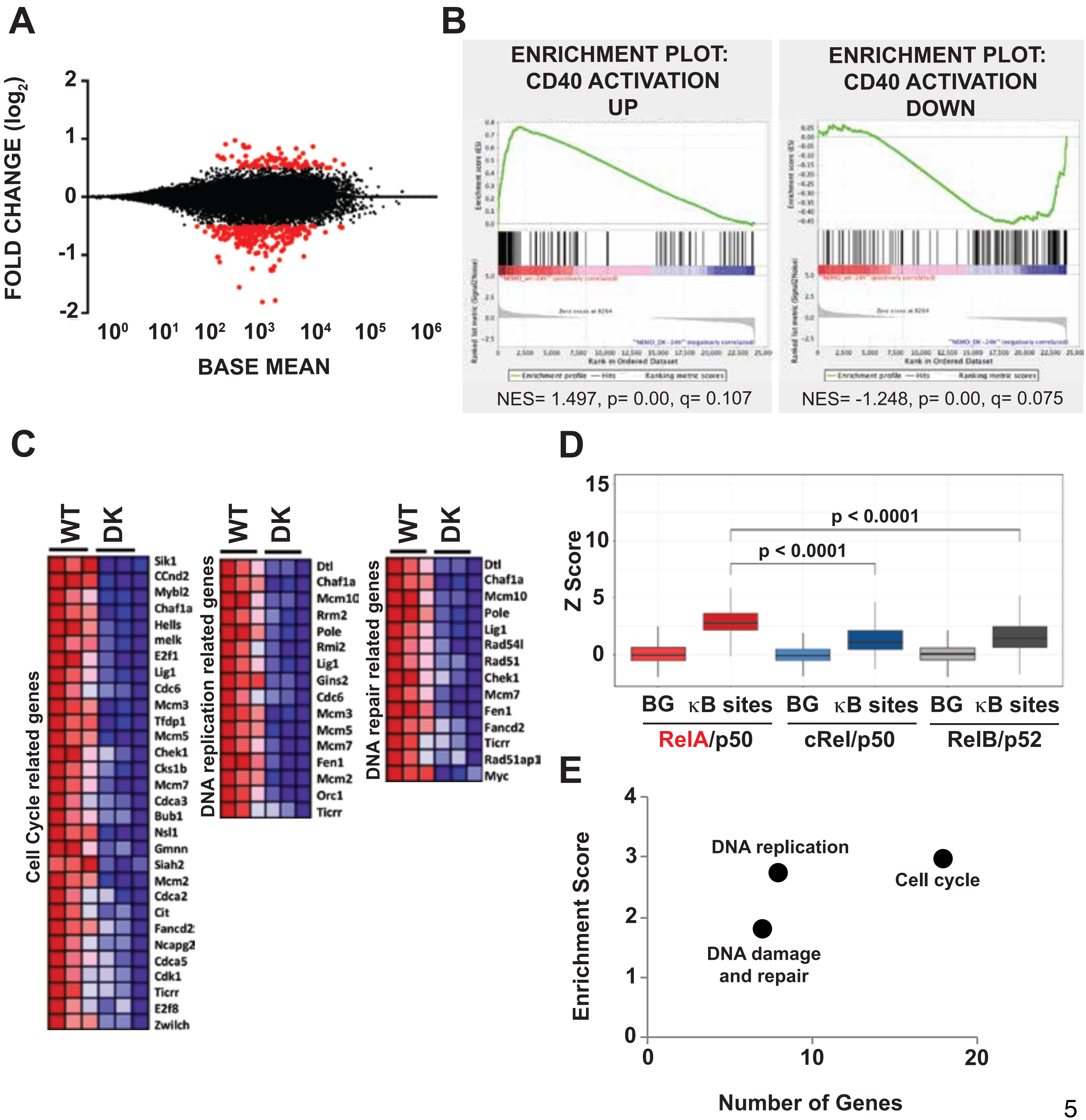

Appendix Figure S2. NEMO^{DK} B cells are defective in CD40-induced gene expression.

(A) RNA-seq analysis of genes in WT and NEMO^{DK} splenic B cells after 24-hour stimulation with CD40. Differentially expressed genes with log2 fold-change and corrected p value ≤0.05 are shown in red. **(B)** GSEA analyses from (A) using the gene signatures from CD40-activated and resting human CD19-positive B cells (GSE54017). **(C)** Heatmap showing the expression of cell cycle, DNA replication, and DNA repair-related genes in WT and NEMO^{DK} B cells with ≥1.5 relative fold change (log2 relative fold change ≥0.585) in (B). **(D)** NF-κB motif Z scores of κB dimer and background (BG) sites are plotted for downregulated genes from (A). P-values were obtained through t-tests comparing the difference between Z-scores of each dimer. **(E)** DAVID analysis of RelA/p50 enriched genes found in (D) highlighting DNA replication, DNA damage and repair and cell cycle related genes.

APPENDIX FIGURE S3

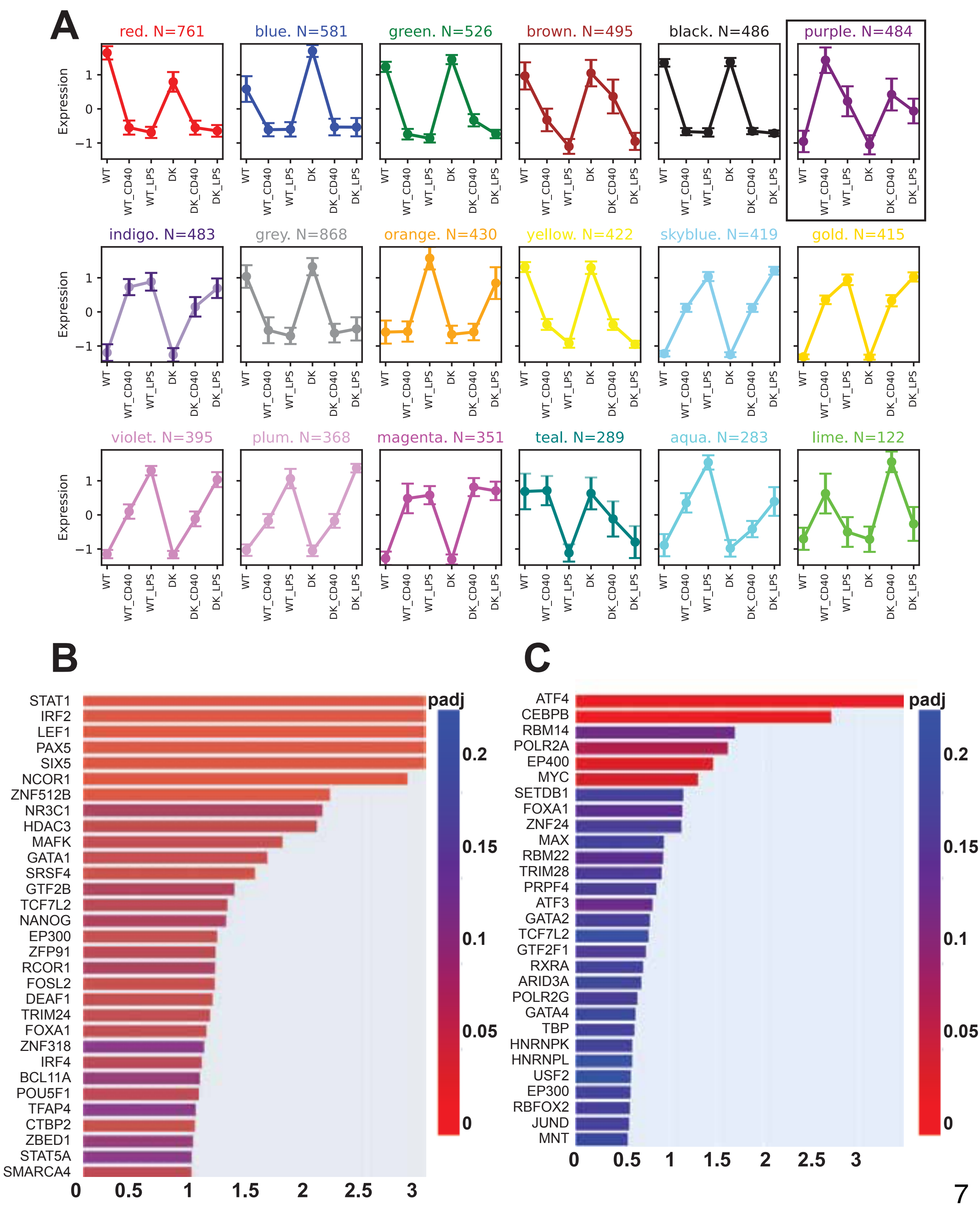

Appendix Figure S3. Gene cluster analysis of unstimulated, CD40+IL-4, and LPS+IL-4 stimulated WT and NEMO^{DK} B cells.

(A) Genes identified from Fig. 5A were subjected to Leiden clustering at a resolution of 0.5 which generated 18 gene clusters. **(B)** MAGIC analysis of the orange cluster from (A), which shows specific defects in LPS+IL-4 induced gene expression in NEMO^{DK} B cells relative to other conditions. RelA is lacking in the top transcription factors predicted to regulate this gene set. **(C)** MAGIC analysis of the aqua cluster from (A), which shows specific defects in CD40+IL-4 and LPS+IL-4 induced gene expression in NEMO^{DK} B cells relative to other conditions. RelA is not found in the top transcription factors predicted to regulate this gene set.

APPENDIX FIGURE S4

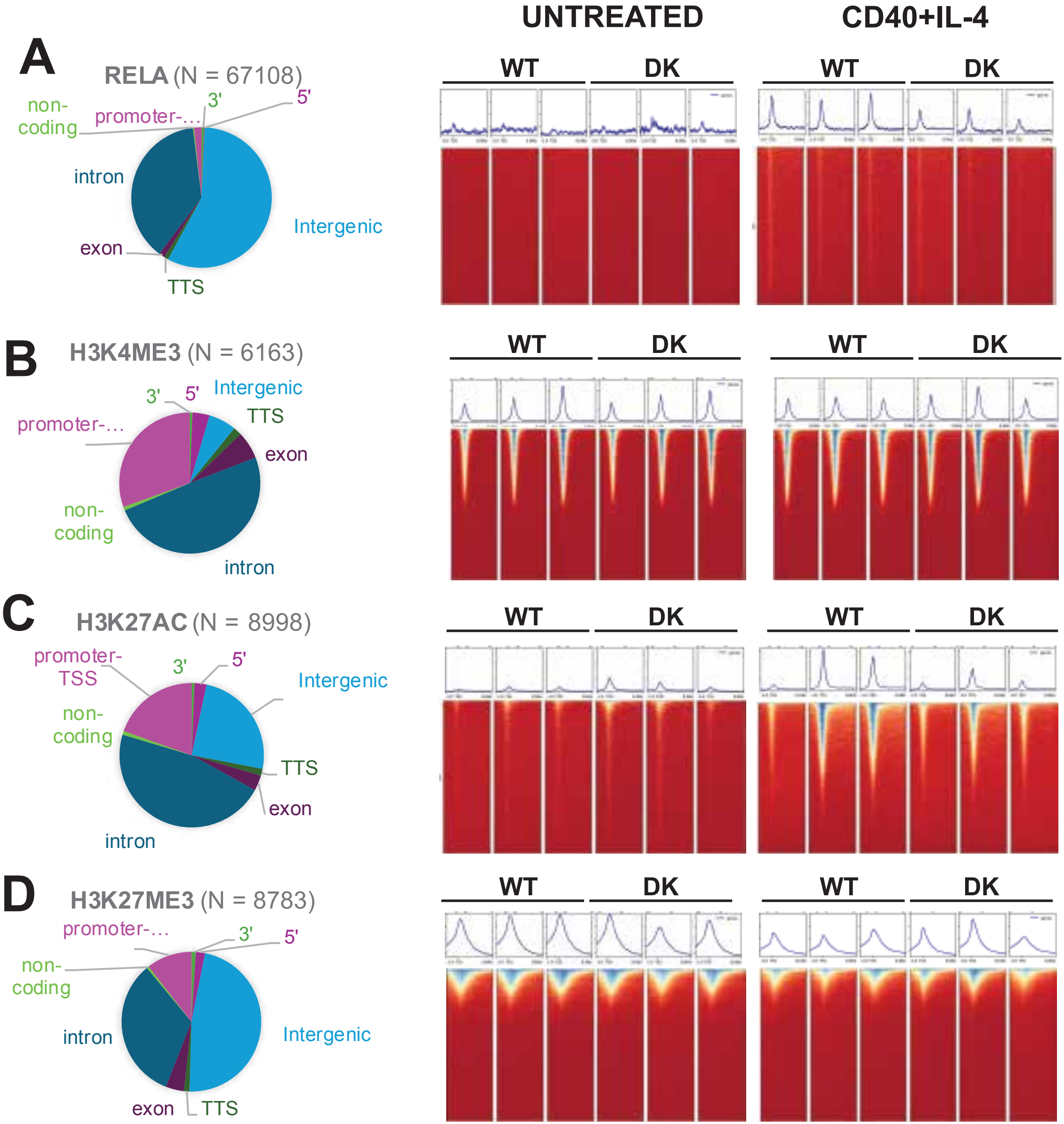

Appendix Figure S4. NEMO^{DK} mutation impairs RelA binding at the promoters of target genes.

CUT&Tag analyses of WT and NEMO^{DK} splenic B cells stimulated with CD40+IL-4 for 2 days. Pie chart of the genomic location distribution of peaks called from WT and NEMO^{DK} replicates and heatmaps of signals aligned to mouse genomes for RelA **(A)**, H3K4me3 **(B)**, H3K27Ac **(C)**, and H3K27me3 **(D)**. All transcriptional start sites (TSS) were aligned and plotted with a 3 kb region upstream of the TSS and 3 kb downstream of the transcription end site (TES) (n=3). While RelA shows statistically significant difference between stimulated WT and NEMO^{DK} replicates, all others, including H3K27Ac, were not statistically significant between the stimulated groups.

APPENDIX FIGURE S5

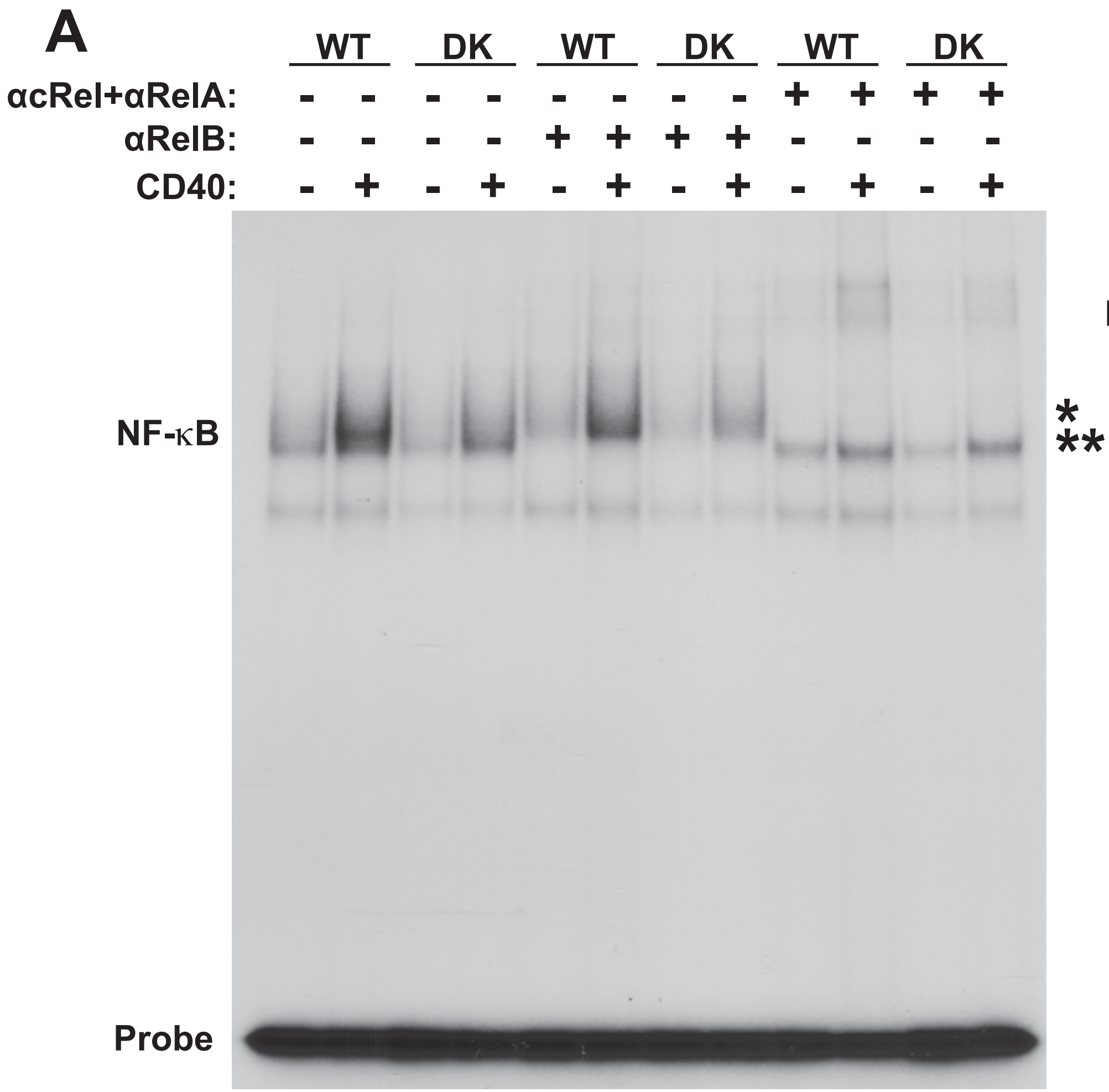

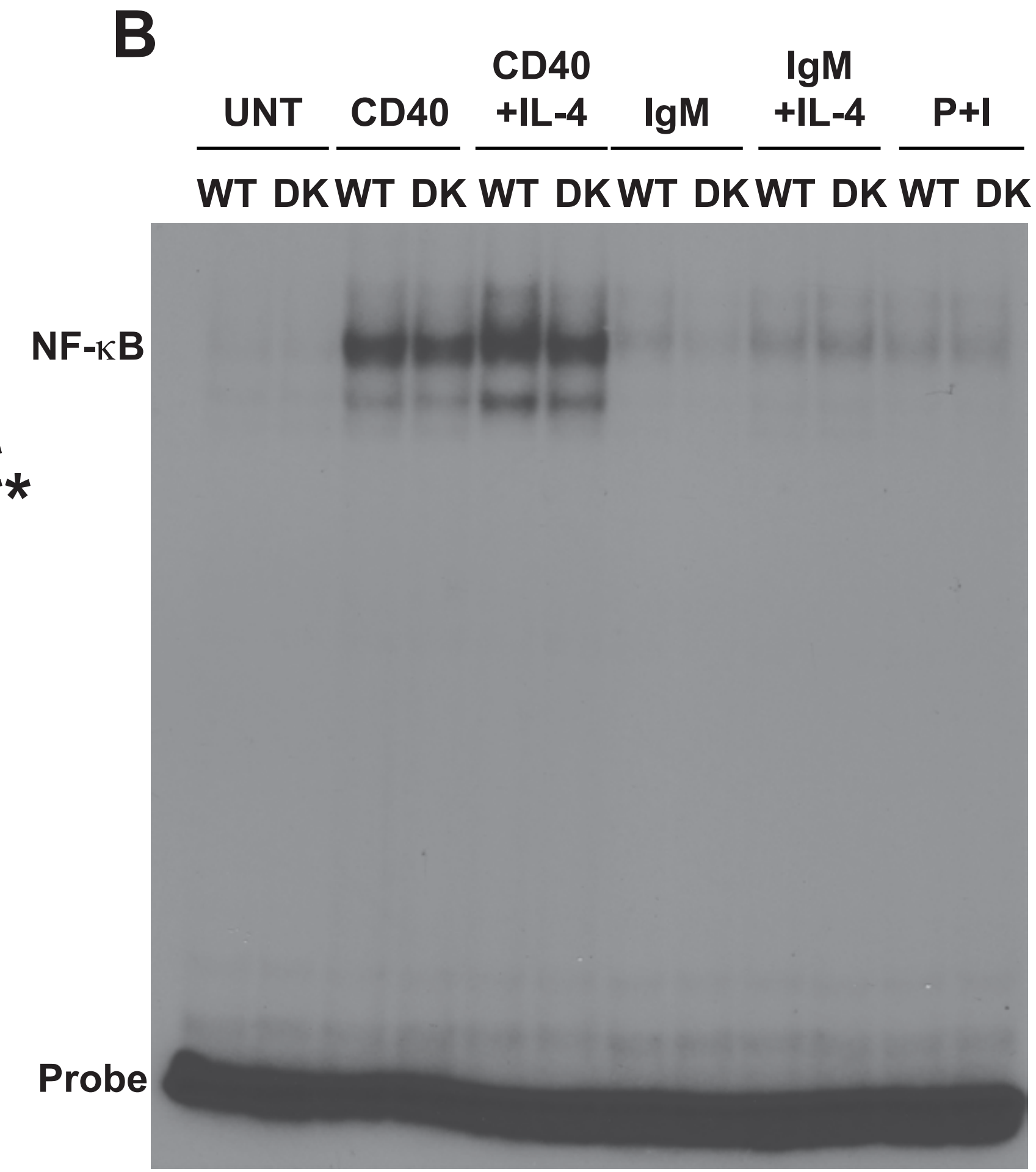

Appendix Figure S5. CD40 is the only stimulus capable of robustly inducing sustained NF-κB activation among several canonical stimuli examined. (A) Full film image of (Fig. 7C) to show the shifted bands of lysates incubated with cRel and RelA antibodies or RelB antibody. Single asterisk marks canonical RelA and cRel complexes; double asterisk marks noncanonical RelB complexes. **(B)** WT and $NEMO^{DK}$ B cells were stimulated as in (Fig. 4A-C) for 48 hours. Lysates from the samples were analyzed for NF-κB activation by EMSA (n=1). EMSA gel was overexposed to highlight the little to weak activation by non-CD40 stimuli at this time point. Activation difference between CD40+/-IL-4 stimulated WT and $NEMO^{DK}$ samples is difficult to discern due to overexposure.

APPENDIX FIGURE S6

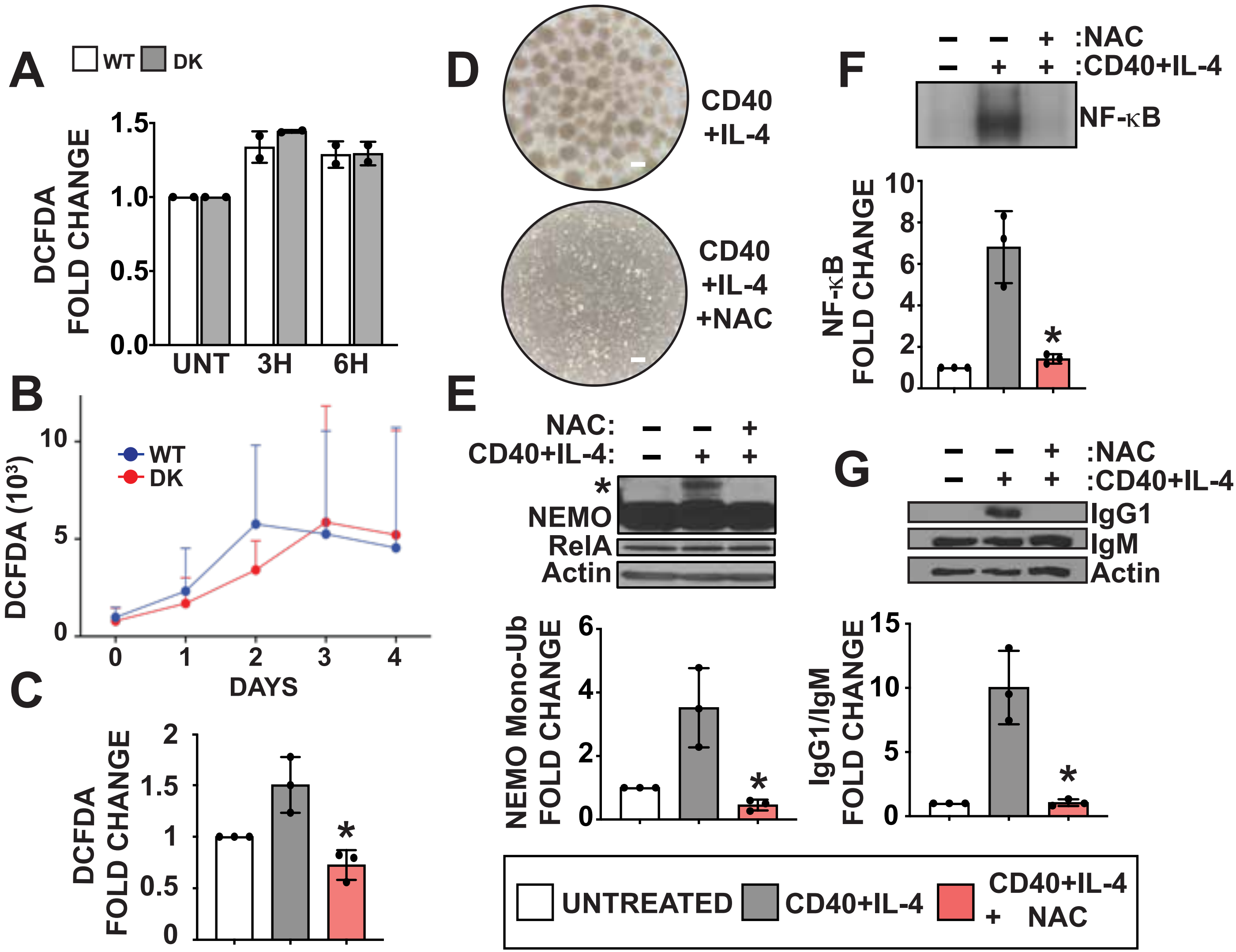

Appendix Figure S6. Inhibition of CD40-induced ROS by NAC diminishes downstream responses. (A) WT and $NEMO^{DK}$ B cells were treated with CD40+IL-4 for 3 and 6 hours, stained with H_2DCFDA, analyzed by flow cytometry. Data is represented as the MFI fold change for each condition normalized to the untreated control, and values are presented as bar graphs showing the mean relative fold change and SD (n=2). **(B)** WT and $NEMO^{DK}$ B cells were treated with CD40+IL-4 over the course of 4 days and stained with H_2DCFDA and analyzed as in (A). Data is represented as a time course plot of the MFI of each genotype at each timepoint with SD (n=3). **(C)** WT B cells treated with either CD40+IL-4 alone or cotreatment with NAC for 24 hours, stained with H_2DCFDA, and analyzed by flow cytometry. Data is represented as in (A, p=0.02). **(D)** Representative HA images of WT B cells treated with either CD40+IL-4 alone or cotreatment with NAC for 4 days. Scale bar on the bottom right represents 100 µm. **(E)** Lysates from the cells in (D) were analyzed by immunoblot for NEMO, RelA, and actin with the asterisk denoting monoubiquitinated NEMO. Quantification of NEMO monoubiquitination is represented as relative fold change compared to untreated cells with mean and SD (n=3, p=0.05). **(F)** Lysates from (D) were analyzed by EMSA for NF-κB activation. Quantification of NF-κB activation is represented as relative fold change compared to untreated cells with mean and SD (n=3, p=0.03). **(G)** WT B cells treated with either CD40+IL-4 alone or cotreatment with NAC for 4 days were collected and analyzed by immunoblot for IgG1, IgM, and actin. Quantification of class switching is represented as the relative fold change of IgG1 over IgM compared to untreated cells with mean and SD (n=3, p=0.05).

Data information: statistical analysis was performed using unpaired two-tailed Welch's t-test. *P* values are indicated: "*" indicates statistically significant.
