# Supplementary material for: NF-κB/RelA signaling required for CD40-induced humoral immunity depends on specific NEMO lysine residues in mice
Source: EMBO J. 2026 Jul 30;45(17):6229–66. doi: 10.1038/s44318-026-00875-0 (PMC13534677; doi:10.1038/s44318-026-00875-0)
Supplement: Supplementary file 3 [file 44318_2026_875_MOESM2_ESM.pdf]

THE EMBO JOURNAL

NF-κB/RelA signaling required for CD40-induced humoral immunity depends on specific NEMO lysine residues in mice

Donna Li, Shelly Wuerzberger-Davis, Yuhong Chen, Funita Phan, Mei Yu, Dominique Lisiero, Cynthia Aguilar, Thomas Cleven, Melba Tejera, Parth Khatri, Natalie Medina, Yu-Chia Chen, Ning Wang, Philippos Tsourkas, Irene M, Ong, Jeffrey Bednarski, Huy Dinh, Alexander Hoffmann, Barry Sleckman, Aussie Suzuki, Avtar Roopra, Marulasiddappa Suresh, Demin Wang, and Shigeki Miyamoto

Corresponding author(s): Shigeki Miyamoto (smiyamot@wisc.edu) , Demin Wang (dwang@versiti.org)

Review Timeline:

Submission Date:	29th May 25
Editorial Decision:	4th Aug 25
Revision Received:	27th Mar 26
Editorial Decision:	26th May 26
Revision Received:	12th Jun 26
Accepted:	22nd Jun 26

Editor: Ioannis Papaioannou

Transaction Report:

(Note: With the exception of the correction of typographical or spelling errors that could be a source of ambiguity, letters and reports are not edited. Depending on transfer agreements, referee reports obtained elsewhere may or may not be included in this compilation. Referee reports are anonymous unless the Referee chooses to sign their reports.)

1st Editorial Decision 4th Aug 2025

Dear Shigeki,

Thank you again for submitting your manuscript EMBOJ-2025-121503 for consideration by The EMBO Journal, and for your patience during peer review. As I have already informed you, your manuscript has been seen by three experts in the field, and we have received their detailed, informative, and constructive reports, which are included below.

I am pleased to say that all three referees are largely supportive, mentioning that the novel insights provided by the manuscript are interesting, relevant, and supported by in vivo evidence. However, they also identify several limitations and raise concerns regarding the interpretation of some data, the design of some experiments, the need to consider alternative possibilities for the interretation of some results (such as the possibility of indirect events linked to DNA damage as potential drivers of CD40 responses), and the fact that many relevant questions remain unanswered. The reviewers provide very detailed lists of suggestions for the improvement of the study and the manuscript.

On balance, and given the positive referees' comments and recommendations, I would like to invite you to submit a thorougly revised version of your manuscript taking the referees' suggestions on board, along with a detailed point-by-point response addressing all referees' comments. In light of the large number of raised issues and unaddressed questions listed by the referees, I would encourage you to share with us a draft provisional revision plan already at the initial stages of your revision, detailing which concerns/questions/suggestions you are willing to address during your revision, and whether there are any major comments you do not agree with or cannot address during your revision. I should add that all technical issues related to the solidity of the data and the robustness of their interpretation must be sufficiently addressed for further consideration of the work at The EMBO Journal.

Please note that it is The EMBO Journal policy to allow only a single round of major revision, and acceptance of your manuscript will therefore depend on the completeness of your responses in this revised version. Please let me know if you have any questions or comments that you would like to discuss with me.

We generally allow three months as standard revision time (November 3, 2025). As a matter of policy, competing manuscripts published during this period will not negatively impact our assessment of the conceptual advance presented by your study. However, we request that you contact us as soon as possible upon publication of any related work, to discuss how to proceed. Should you foresee a problem in meeting this three-month deadline, please let us know in advance and we will be able to grant an extension.

Thank you for the opportunity to consider your work for publication in The EMBO Journal. I look forward to your revision.

Best regards,

Ioannis

Ioannis Papaioannou, PhD
Editor, The EMBO Journal
i.papaioannou@embojournal.org

*****
Instructions for preparing your revised manuscript
*****

1. When you are ready to submit the revision, please upload:

- A Word file of the manuscript text (including legends of main Figures, EV Figures and Tables). Please make sure that changes are highlighted (or "tracked") to be clearly visible.

- Individual production-quality figure files (one file per figure). When assembling your figures, please refer to our figure preparation guidelines in order to ensure proper formatting and readability in print as well as on screen:
https://bit.ly/EMBOPressFigurePreparationGuideline
If the data shown in a figure are obtained from n {less than or equal to} 2, please use scatter plots showing the individual data points.
Please also include scale bars in all microscopy images.
The following points must be specified in each figure legend:
i. the name of the statistical test used to generate error bars and P values

ii. the number (n) of independent experiments (please specify technical or biological replicates) underlying each data point (discussion of statistical methodology can be reported in the Materials and Methods section, but figure legends should contain a basic description of n, P, and the test applied)
iii. the nature of the bars and error bars (s.d., s.e.m.).
See also guidelines for figure legends: https://www.embopress.org/page/journal/14602075/authorguide#figureformat

- A point-by-point response to the referees' comments, with a detailed description of the changes made (as a word file). All referees' concerns must be fully addressed and their suggestions taken on board. When preparing your letter of response to the referees' comments, please bear in mind that this will form part of the Review Process File and will therefore be available online to the community. Please note that you have the possibility to opt out of the transparent process at any stage prior to publication by letting the editorial office know (contact@embojournal.org); if you do opt out, the Review Process File link will point to the following statement: "No Review Process File is available with this article, as the authors have chosen not to make the review process public in this case.". For more details on our Transparent Editorial Process, please visit our website: https://www.embopress.org/page/journal/14602075/authorguide#transparentprocess

- Expanded View (EV) files (replacing Supplementary Information) that are collapsible/expandable online. A maximum of 5 EV Figures can be typeset. EV Figures should be cited as "Figure EV1, Figure EV2" etc. in the text, and their respective legends should be included in the manuscript file after the legends of regular figures. See detailed instructions regarding Expanded View files here:
https://www.embopress.org/page/journal/14602075/authorguide#expandedview

- For the figures that you do NOT wish to display as Expanded View figures, they should be bundled together with their legends in a single PDF file called "Appendix", which should start with a short Table of Contents (including page numbers). Appendix figures should be referred to in the main text as: "Appendix Figure S1, Appendix Figure S2" etc. Please see detailed instructions here: https://www.embopress.org/page/journal/14602075/authorguide#expandedview

- A complete author checklist, which you can download from our author guidelines (https://www.embopress.org/page/journal/14602075/authorguide). Please note that the checklist will also be part of the Review Process File.

2. Please note that no statistics should be calculated and shown in Figures if n=2. Please also note that each p value should be reported as an exact value.

3. Before submitting your revision, primary datasets (and computer code, where appropriate) produced in this study need to be deposited in appropriate public databases (see https://www.embopress.org/page/journal/14602075/authorguide#dataavailability). In particular, we kindly request you to submit all DNA/RNA/ATAC sequencing data produced in your study to appropriate repositories. The accession numbers, database, and the specific URLs (links) should be listed in a formal "Data availability" section (placed after Methods), following the example below:

"The RNA-seq datasets produced in this study are available in the following database:
Gene Expression Omnibus GSE46843 (https://www.ncbi.nlm.nih.gov/geo/query/acc.cgi?acc=GSE46843)"

*** All links should resolve to a page where the data can be accessed. ***

*** Please remember to provide in the Data availability section of your revised manuscript reviewer passwords if the datasets are not yet public. ***

*** The Data Availability Section is restricted to new primary data that are part of this study. In case you have no data that require deposition in a public database, please state so instead of referring to the database: "Our study includes no data deposited in public repositories." under the heading "Data availability". ***

4. The materials and methods need to be described in the manuscript using our structured methods format, which is now required for all research articles. According to this format, the Methods section includes a single "Reagents and Tools Table" - listing key reagents, experimental models, software and relevant equipment including their sources and relevant identifiers- followed by a "Methods and Protocols" section describing the methods. Please download and fill our Reagents and Tools Table template (.docx), which you can find in our author guide:
https://www.embopress.org/page/journal/14602075/authorguide#structuredmethods. When submitting your revised manuscript, please do not include the Reagents and Tools Table in the Methods section of the manuscript but instead upload it as a separate file choosing the file type "Reagent Table".

5. Please check that the title and the abstract of the manuscript are brief, yet explicit, even to non-specialists. The length of the title should not exceed 100 characters, and the abstract should be a single paragraph not exceeding 175 words.

6. Please also note our reference format: https://www.embopress.org/page/journal/14602075/authorguide#referencesformat.

7. At EMBO Press we ask authors to provide source data for the main manuscript figures. You will receive a separate email with instructions for providing source data with your revised manuscript, including how to upload and organize the files.

8. Please remember: digital image enhancement is acceptable practice, as long as it accurately represents the original data and conforms to community standards. If a figure has been subjected to significant electronic manipulation, this must be noted in the figure legend or in the "Materials and Methods" section. The editors reserve the right to request original versions of figures and the original images that were used to assemble the figure.

9. Our journal encourages inclusion of data citations in the reference list to directly cite datasets that were obtained from public databases. Data citations in the article text are distinct from normal bibliographical citations and should directly link to the database records from which the data can be accessed. In the main text, data citations are formatted as follows: "Data ref: Smith et al, 2001" or "Data ref: NCBI Sequence Read Archive PRJNA342805, 2017". In the Reference list, data citations must be labeled with "[DATASET]". A data reference must provide the database name, accession number/identifiers, and a resolvable link to the landing page from which the data can be accessed at the end of the reference. Further instructions are available at: https://www.embopress.org/page/journal/14602075/authorguide#referencesformat.

10. We request authors to consider both actual and perceived competing interests. Please review our policy (https://www.embopress.org/page/journal/14602075/authorguide#conflictsofinterest) and update your competing interests statement if necessary. Please name this section 'Disclosure and competing interests statement' and place it after the Acknowledgements section.

11. Please note that all corresponding authors are required to provide an ORCID ID upon submission of a revised manuscript (https://orcid.org/). Please find instructions on how to link your ORCID ID to your account in our manuscript tracking system in our Author guidelines
(https://www.embopress.org/page/journal/14602075/authorguide#authorshipguidelines).

12. We use CRediT to specify the contributions of each author in the journal submission system. CRediT replaces the author contribution section, which should be removed from the manuscript. Please use the free text box to provide more detailed descriptions. See also guide to authors:
https://www.embopress.org/page/journal/14602075/authorguide#authorshipguidelines.

13. Further information is available in our Guide For Authors: https://www.embopress.org/page/journal/14602075/authorguide

14. We would also welcome the submission of cover suggestions or motifs to be used by our Graphics Illustrator in designing a cover.

15. Please use the link below to submit your revision:
https://emboj.msubmit.net/cgi-bin/main.plex

Referee #1:

In their manuscript entitled "ROS-dependent NEMO modification sustains high RelA activity for optimal CD40-induced humoral immunity", Li et al. present a well-constructed and methodologically concise study that addresses significant mechanistic aspects of clinically relevant mutations in NEMO (NF-κB essential modulator). Their manuscript contributes meaningfully to our understanding of immunodeficiencies and NF-κB signaling. A major strength of the study is its in vivo characterization of two critical NEMO mutations, namely lysines 270 and 302 in mouse NEMO. The authors demonstrate the pathological link between altered NEMO function mediated by defective mono-ubiquitination and impaired immune responses in B-cell malignancies. Importantly, this study evaluates the functional consequences of defective CD40 signaling, including impaired antibody responses, and altered gene expression profiles specific to mutant NEMODK in vivo.
Thereby, this manuscript provides valuable insight into the pathogenic role of NEMO mutations in immunodeficiency, supported by robust in vivo evidence. Taken together, these findings suggest that the NEMO/NF-κB signaling pathway has a role in the development of autoimmunity. There are however, significant concerns with data interpretation and experimental design that demand careful revision.

Major points:

Figure 1:

1. As the study employs an infection model, it is interesting to speculate whether the impaired antibody response of NEMODK mice may have implications for viral clearance or immunity. Even if not measured quantitatively, the authors need to remark whether mutant mice showed an increase in viral burden or prolonged infection, stressing the functional importance of the NF-κB-dependent humoral response.
2. The authors quantify the numbers of $CD4^+$ and $CD8^+$ T cells in the spleen, and report no significant differences between WT vs NEMODK mutant mice. However, this analysis is limited to cell counts and does not provide any insight on T-cell functionality. Given the well-established role of NEMO in TCR-mediated NF-κB signaling, it is critical to examine whether T-cell responses are functionally intact, particularly under stimulation-dependent conditions. Specifically, the authors should assess: (1) $CD4^+$ T cell activation following anti-CD3/CD28 or antigen-specific stimulation; (2) T follicular helper (Tfh) cell differentiation, as these cells are essential for effective B-cell help and germinal centre responses; and (3) $CD8^+$ T cell effector functions, including cytotoxicity and cytokine output, particularly in light of the widespread downregulation of NF-κB target and DNA damage response genes. The current lack of such functional data weakens the interpretation that the immune defects are only B cell-intrinsic. We strongly recommend that the authors perform functional T cell assays, to assess T cell activation, cytokine secretion, and helper capacity.

Figure 2:
3. In Figure 2D, the authors claim that the mutant cells form smaller B-cell homotypic aggregates (HA) in response to stimulation of CD40 in the presence of IL-4. However, this conclusion lacks sufficient experimental support. Kinetic data, e.g., time-course of aggregation formation or disassembly is required to confirm reliability of the conclusion.
4. B-cell defects are evident upon CD40 stimulation, yet LPS stimulation does not seem to reveal similar impairments. Both CD40 and LPS are well-established activators of NF-κB signaling in B cells, albeit through distinct upstream receptors. Nevertheless, both converge on IKK complex activation and NF-κB nuclear translocation. Given that the NEMODK mutation is proposed to affect NEMO ubiquitination and downstream NF-κB signaling, one would expect defects in both CD40- and LPS-induced responses if the mutation causes a general impairment of canonical NF-κB signaling in B cells. The fact that LPS responses appear preserved raises several critical questions:
(1) Is the mutation selectively affecting CD40-induced signaling complexes and not LPS-stimulated pathways? If so, what is the molecular explanation for this specificity?
(2) Do the authors observe normal NEMO ubiquitination or NF-κB activation in B cells downstream of LPS in the mutant?
(3) Could there be redundant or compensatory mechanisms engaged by LPS that bypass the NEMO defect?
(4) Does this mean that TLR4-MyD88 NF-κB activation does not require NEMO? And if so could they show how this happen because the known published work argues against that.
5. This selective defect in CD40 but not LPS responses should be more thoroughly investigated and mechanistically explained. Ideally, the authors should provide side-by-side signaling and functional assays comparing CD40 and LPS stimulation in WT and mutant B cells, including measurements of NF-κB activation (e.g., p-p65, p-IKK). Moreover, authors should explain how LPS differentially activate MyD88-dependent signalling or TRIF-dependent signalling in their system.

Figure 3:
6. This figure overall, highlights a notable decrease in RelA in NEMODK mutant, alongside suppression of key DNA damage response genes such as Trp53, Brca1, and Atg7. This widespread transcriptional repression, especially involving pro-survival and stress response genes, would conventionally be expected to render cells more susceptible to impaired viability. Yet, the authors report no measurable increase in cell death, nor do they investigate compensatory mechanisms that might preserve cell survival. This raises two critical questions:
(1) If one of the most pivotal pro-survival pathways is impaired, why is there no compensatory upregulation of alternative survival or stress-response pathways (e.g., autophagy-related genes)?
(2) How do cells maintain viability despite such broad molecular defects in pro-survival signaling?
7. Furthermore, the functional implications of this molecular repression remain underexplored. While the authors note that the mutant mice are viable, the immune signature of these NEMO-deficient cells is not clearly defined, particularly in terms of inflammatory cytokine storm. The manuscript refers to functional antibody defects, but does not address the downstream consequences of impaired humoral immunity. Are these mice more susceptible to infections? Is there altered vaccine responsiveness, or reduced affinity maturation? A more detailed immunophenotypic analysis and discussion are required.
All of the omics data are at the 48-hour time point. The authors should do (or at least report on) the kinetics of these transcriptional differences. When after stimulation do WT and NEMODK gene expression profiles diverge? The NF-κB activity data (Fig. 5) are consistent with diverging as early as a few hours and are evident by 24-48 h.

Figure 4:
8. This figure presents data on the expression of NBN and AID, both of which are well-known players in the DNA damage response and class-switch recombination (CSR). A direct mechanistic link is missing that ties their downregulation to NEMO-specific ubiquitination defects or downstream functional outcomes. It would significantly strengthen the manuscript if the authors clarified (1) how their regulation is influenced by NEMO mutation/ dysfunction, and (2) what functional consequences arise from their suppression (dependent of NEMO mutation), particularly regarding CSR.
9. As NBS1 and AID are stated to be downstream of NF-κB RelA, the authors need to support this assertion with proofs of direct interaction. Aside from Nbn and Aicda, are there any other key NF-κB target genes left to be validated? The authors can check genes that were also present in their screen.

Figure 5:
10. In Figure 5, specifically in panels 5A, D and G, EMSAs are presented to assess NF-κB activation following CD40 stimulation. Immunoblots for phosphorylated p65 (RelA) should also be performed as a more direct measure of canonical NF-κB pathway activation.
11. Furthermore, the quantified differences shown in Figure 5E are not clearly reflected in the EMSA images in 5D. The band shifts are faint, do not show NF-kB dimers and do not convincingly demonstrate a dynamic difference in NF-κB activity between wild-type and mutant cells. The reported divergence at later time points (3-6 hours) raises mechanistic questions about the temporal regulation and feedback loops within the NF-κB pathway, issues that the authors do not sufficiently address. To strengthen this figure, we strongly recommend the inclusion of immunoblots for phosphorylated p65 (p-RelA) and phosphorylated IKKα/β. We also suggest inhibiting IKKs using an inhibitor to neutralize the effect shown to assert specificity of the difference in activation. It is known that canonical NF-κB complex could be either p50-p65 heterodimer or p50-p50 homodimer, both of them have different effect on downstream signalling. It would be of value for the authors to comment on this, employing western blots showing the phospho-p105 and p50 product.
12. The authors showed extensively in figure 3 that RelA is transcriptionally downregulated in response to CD40 in NEMODK mutant, however in figure 5F, the protein expression levels of RelA are equal amongst NEMOWT and NEMODK mutant. What is authors´ comment on this?
13. In figure 5G the authors present data showing NF-κB signaling activation upon CD40 stimulation for up to 4 days, but do not consider/validate the half-life or turnover of the CD40 receptor. In vivo, CD40-CD40L interactions are transient. The authors should clarify:
• What is the expected half-life of CD40 on B cells under their experimental conditions?
• Is CD40 expression monitored over the 4-day time course?
• Could prolonged CD40 engagement lead to secondary effects unrelated to canonical signaling, such as altered cell viability or exhaustion?
To address these concerns, the authors are advised to provide time-resolved CD40 surface expression data.

Figure 6:
14. In figure 6A, the authors show that upon CD40 stimulation, NEMOWT get mono-ubiquitinated whereas NEMODK mutant does not. To confirm this, it is necessary to perform an immunoprecipitation for ubiquitin and present immunoblot for NEMO. Also, as we are looking for changes in post-translational modifications in NEMO WT vs DK, it is important to include full-length blots of NEMO and ubiquitin.
15. Figures 6C and D aim to demonstrate that CD40 stimulation in 70Z/3 cells stably expressing mCD40 induces mono-ubiquitination of NEMO. However, the message is redundant across the two figures. Figure 6C, showing the 24-hour stimulation time point, presents the data with clearer and better-resolved blots, making it easier to interpret. In contrast, Figure 6D attempts to show a time course, but the blots are poor in quality, with weak and poorly distinguishable bands that do not add substantial new insight beyond what is already conveyed in 6C. We suggest that Figure 6C be retained in the main manuscript, while Figure 6D be either redone with improved blot quality and quantification or moved to the supplementary material.
16. In Figure 6G it looks as if the labelling order for the enzymes is incorrect or interchanged, please verify. In principle, DUB (USP2) + should cleave the K63 chains and SENP1 + should cleave SUMO chains.

Referee #2:

In the present manuscript, Miyamoto and colleagues generated knock-in (KI) mice to analyze the in vivo role of NEMO lysines 270 and 302. Sumoylation or mono-ubiquitination of these lysines has been shown to drive NF-kB activation upon DNA damage response (e.g. ionizing radiation or genotoxic agents). NEMO DK mice, with conservative KK270/302RR exchanges, are viable. A decrease in testes size (not further analyzed) and a reduction in IgA and IgG1 levels is the main phenotypic alteration under homeostatic conditions. Humoral immune response upon LCMV infection are compromised. The authors show mildly impaired CD40-triggered B cell activation in vitro (proliferation, IgG1 class switching), while BCR and LPS stimulation remained unchanged. Transcriptomic and molecular profiling suggested that sustained activation of NF-kB RelA is reduced in NEMO DK mice. NEMO mono-ubiquitination in response to CD40 stimulation is absent in NEMO DK B cells. CD40 also induces reactive oxygen species (ROS) and blockade of this the antioxidant N-acetyl cysteine (NAC) abrogates in vitro CD40-induced mono-ubiquitination, NF-kB activation and IgG1 class switching.
Overall, the study describes the first attempt to unravel an in vivo role of lysines 270/302 in NEMO. It describes some interesting and unexpected effects on impaired CD40 stimulation in NEMO DK B cells. However, the underlying mechanism is unclear. Further, in vitro NEMO modifications at lysines 270 and 302 have been connected to NF-kB activation after DNA-damage response, but there has been no attempt to test this in the physiological in vivo setting.

Major concerns:

There are mild effects on CD40 stimulation, but lysines 270/302 in NEMO do not seem to be main drivers of CD40 responses. The lysines rather modulate sustained responses (pro-longed RelA activation), which could arise - as the reviewers note in the discussion - from indirect events such as DNA damage which is of course happening in B cells undergoing class switch recombination and somatic hypermutations. The Miyamoto laboratory reported pioneering findings on the unconventional role of

NEMO and lysines 270 and 302 in DNA damage-induced NF-kB activation. It is unclear why the study focusses on rather mild alterations in B cells, while potential changes in DNA damage responses have not been investigated. What happens if these mice are exposed to ionizing radiation or chemotherapeutic agents? Can tumors be induced and are therapeutic responses to DNA-damaging reagents in NEMO DK mice altered?

Basal IgG1 and IgA levels are reduced in NEMO DK mice. Instead of only testing B cell responses upon LCMV infection, it will be important to see in how antibody class switching, and antigen-specific immune responses are altered upon immunization with T cell-dependent an/or -independent antigens. Is the defect indeed at the GC reaction, which elicits less plasma cells and reduced humoral immune responses? Is this connected with an impaired DNA damage response in GCB cells undergoing class switching and somatic hypermutations?

In figure 7 the authors show that CD40 induces ROS induction. NAC treatment prevents B cell responses to CD40, but the connection to the previous data is unclear. ROS induction is not altered in NEMO DK cells. Despite the correlative observation that high concentrations (50 mM) of NAC (a strong antioxidant) abolish NEMO mono-ubiquitination and NF-kB activation as well as antibody class switching, there is no evidence that this is linked to the much milder effects observed in NEMO DK mice. It is well established that IKK/NF-kB signaling is highly sensitive to the redox status of the cells and most likely all NF-kB activating pathways are affected by 50 mM NAC. Again, why have NEMO DK B cells (or other cells) not been subjected to DNA-damage reagents that induce NF-kB?

Specific points:

1) NF-kB signaling has been implicated in many processes of B cell differentiation. Are there any changes in marginal zone or follicular B cells? Is the ratio of B1(a/b) to B2 cells affected?

2) How severe is the defect of NEMO DK in coping with LCMV infection. Despite failing to mount optimal LCMV-specific humoral immune response, are NEMO DK mice unable to control viral titers or are they more easily succumbing to LCMV infection?

3) Are plasma cells reduced after LCMV infection in NEMO DK mice?

4) CD40 can boost BCR-induced NF-kB activation and cell proliferation. Has the combination of CD40 and BCR stimulation been tested on cell proliferation and class switching?

5) It seems that NEMO DK mice do not show an altered induction in the classical canonical or non-canonical NF-kB target gene signatures after CD40 stimulation. This is somewhat surprising given that RelA is the main transcription factor affected by NEMO DK. Have the authors closely looked at NF-kB target gene signatures?

6) Fig. S5: In the aqua cluster CD40 and LPS responses are affected. Can the authors comment? Are there LPS-induced NF-kB target genes changed in this cluster?

7) It should be investigated if the reduction in sustained RelA activation in NEMO DK cells is indeed specific for CD40 stimulation and not seen after LPS or BCR stimulation.

8) The antibody-supershift assay is not a good quantitative method to determine levels of active NF-kB subunits. NF-kB DNA-binding ELISA kits exist for each subunit, which is a much better way to quantify the relative changes in the various active NF-kB subunits.

Minor points:

Fig. S2A: Indicate which gate corresponds to which B cell population.

Fig. 2A: The presentation of cell cycle in this form is not very helpful.

Fig. 4A seems to be misplaced and should be Fig. S7A. Subsequent labeling needs to be adjusted.

Referee #3:

This report from Li and colleagues presents intriguing findings to suggest that CD40-induced NEMO monoubiquitination (monoUb) on lysines residues 270 and 302 is required for sustained, ROS-dependent canonical NF-kB activation following CD40 engagement. The authors show that "NEMO-DK" mice, in which both lysines are switched to arginines, show a marked defect in CD40-induced B cell proliferation and class switch recombination (CSR), both in vitro and in response to LCMV infection in vivo. These functional deficits appear to be B cell-intrinsic and correlated with the absence of NEMO monoUb - this ROS-dependent post-translational modification is apparently required for sustained RelA activity and downstream transcription of genes required

for cell cycle progression, DNA damage repair (e.g. NBS1) and CSR (e.g. AID), especially for IL-4-dependent IgG1 generation.

This manuscript is outstanding overall, providing convincing and comprehensive mechanistic evidence linking a novel NEMO PTM to prolonged canonical NF-kB activation required for B cell differentiation and function downstream of CD40 ligation. Their highly competent use of multiple next-generation sequencing modalities and bioinformatics analyses to inform these findings at the transcriptional level is particularly notable. Relatively inconsequential critiques outlined below should be addressed prior to publication.

Major critiques:

• One major question is how CD40 ligation triggers acute canonical NF-kB activation, considering CD40 signaling is traditionally associated with the noncanonical NF-kB pathway. The authors cite a single reference (Zarnegar et al), but this point could be elaborated on further in the introduction and discussion.

• Figure 2B-C: From the data presented, it may be premature to conclude that IgM-induced proliferation is normal in NEMO-DK mice - the agonistic Ab used appears to largely induce cell death based on the high proportion of sub-G1 apoptotic cells in their cell cycle analyses. B cell receptor-induced proliferation typically requires extensive crosslinking of the anti-IgM/D Ab to work properly, which may be worth trying here for a better comparison or at least acknowledging in the discussion.

• Figure 2E-F: Why did the authors not measure IgG3 or IgG2b production in response to CD40 ligation (+ a relevant cytokine)? LPS-induced class switching to these isotypes appears comparable in WT and NEMO-DK mice but quite modest overall. This question seems potentially important, as Figure 1 presents a discrepancy between genotypes in relation to total IgG2/3 levels (1A) vs. LCMV-induced IgG2a/b levels (1H).

• Discussion: the authors generally do a good job of considering study limitations, but they could elaborate further on putative ROS categories downstream of CD40 ligation that might be at play in stimulating NEMO monoUb, given their convincing data using the broad antioxidant NAC.

Minor critiques:

• Page 7: the authors' description of BM chimera experiments presented in Figure 1 is somewhat confusing - it should be clarified in the text that WT and NEMO-DK BM were not tested together in a competitive experiment, but rather mixed 1:4 with mMT B cells as described in the methods.

• Supp Fig 6C: although variable, H3K27 acetylation appears lower in NEMO-DK B cells - can these epigenetic marks be quantified in some way?

• Figure 5C: it would be helpful to display the entire blot to view supershifted complexes on the EMSA

• Figure 6G: labels at the bottom of each blot appear to be switched

• Page 5: change "osteopetrosis" to "osteoporosis"

• Page 9: change "changes in cell death does not account" to "do not account"

General statement:

We thank all the referees for taking time and carefully going through our initial manuscript. We have addressed all the points raised by each of the referees below. As you will see (highlighted in blue texts), we have addressed most concerns by performing additional experiments. Given that most of these new experiments required matched WT and NEMO^{DK} littermates for analysis and the number of such mice was limiting even with our effort to increase the breeding capacity, we apologize in advance that we were unable to perform all the requested experiments. The list of new experiments performed are summarized at the end of the point-by-point response described below for quick reference.

Referee #1:

In their manuscript entitled "ROS-dependent NEMO modification sustains high RelA activity for optimal CD40-induced humoral immunity", Li et al. present a well-constructed and methodologically concise study that addresses significant mechanistic aspects of clinically relevant mutations in NEMO (NF-κB essential modulator). Their manuscript contributes meaningfully to our understanding of immunodeficiencies and NF-κB signaling. A major strength of the study is its in vivo characterization of two critical NEMO mutations, namely lysines 270 and 302 in mouse NEMO. The authors demonstrate the pathological link between altered NEMO function mediated by defective mono-ubiquitination and impaired immune responses in B-cell malignancies. Importantly, this study evaluates the functional consequences of defective CD40 signaling, including impaired antibody responses, and altered gene expression profiles specific to mutant NEMODK in vivo.

Thereby, this manuscript provides valuable insight into the pathogenic role of NEMO mutations in immunodeficiency, supported by robust in vivo evidence. Taken together, these findings suggest that the NEMO/NF-κB signaling pathway has a role in the development of autoimmunity. There are however, significant concerns with data interpretation and experimental design that demand careful revision.

We thank the reviewer for their positive comments of our study.

Major points:

Figure 1:

1. As the study employs an infection model, it is interesting to speculate whether the impaired antibody response of NEMODK mice may have implications for viral clearance or immunity. Even if not measured quantitatively, the authors need to remark whether mutant mice showed an increase in viral burden or prolonged infection, stressing the functional importance of the NF-κB-dependent humoral response.

We have now carried out an additional LCMV infection study and provided viral titer data (**new Fig. EV2E**). Since the clearance of LCMV-Armstrong is mostly $CD8^+$ T cell mediated, where we did not find any defect in the mutant mice (**new Fig. EV2B-D**), we also found similar viral clearance in the $NEMO^{DK}$ mutant mice as in WT mice at day 8 post-infection.

2. The authors quantify the numbers of $CD4^+$ and $CD8^+$ T cells in the spleen, and report no significant differences between WT vs NEMODK mutant mice. However, this analysis is limited to cell counts and does not provide any insight on T-cell functionality. Given the well-established role of NEMO in TCR-mediated NF-κB signaling, it is critical to examine whether T-cell responses are functionally intact, particularly under stimulation-dependent conditions. Specifically, the authors should assess: (1) $CD4^+$ T cell activation following anti-CD3/CD28 or antigen-specific stimulation; (2) T follicular helper (Tfh) cell differentiation, as these cells are essential for effective B-cell help and germinal centre responses; and (3) $CD8^+$ T cell effector functions, including cytotoxicity and cytokine output, particularly in light of the widespread downregulation of NF-κB target and DNA damage response genes. The current lack of such functional data weakens the interpretation that the immune defects are only B cell-intrinsic. We strongly recommend that the authors perform functional T cell assays, to assess T cell activation, cytokine secretion, and helper capacity.

As in above point, we have performed additional LCMV infection studies and provided data on anti-viral $CD8^+$ and $CD4^+$ T cell responses, including T_{FH} cell responses *in vivo* (**new Fig. EV2B-D**). As clearly seen, the anti-viral $CD8^+$ and $CD4^+$ T cell responses were normal in $NEMO^{DK}$ mice. Given this lack of phenotype and the limited quantity of $NEMO^{DK}$ mice, we refrained from conducting additional *in vitro* studies on $CD8^+$ and $CD4^+$ T cells.

Figure 2:

3. In Figure 2D, the authors claim that the mutant cells form smaller B-cell homotypic aggregates (HA) in response to stimulation of CD40 in the presence of IL-4. However, this conclusion lacks sufficient experimental support. Kinetic data, e.g., time-course of aggregation formation or disassembly is required to confirm reliability of the conclusion.

We now added additional data to address this question in **new Figure 3**. We provided time-lapse imaging data (**new Figure 3B**). We also provided quantitative analysis of the resulting HA sizes in **new Figure 3D**. Finally, we also included new data demonstrating the reduced expression of ICAM-1 mRNA in **new Figure 3E** to further support the defective expression of this cell adhesion molecule gene, which has been previously demonstrated to mediate homotypic aggregation.

4. B-cell defects are evident upon CD40 stimulation, yet LPS stimulation does not seem to reveal similar impairments. Both CD40 and LPS are well-established activators of NF-κB signaling in B cells, albeit

through distinct upstream receptors. Nevertheless, both converge on IKK complex activation and NF-κB nuclear translocation. Given that the NEMODK mutation is proposed to affect NEMO ubiquitination and downstream NF-κB signaling, one would expect defects in both CD40- and LPS-induced responses if the mutation causes a general impairment of canonical NF-κB signaling in B cells. The fact that LPS responses appear preserved raises several critical questions:

(1) Is the mutation selectively affecting CD40-induced signaling complexes and not LPS-stimulated pathways? If so, what is the molecular explanation for this specificity?

(2) Do the authors observe normal NEMO ubiquitination or NF-κB activation in B cells downstream of LPS in the mutant?

(3) Could there be redundant or compensatory mechanisms engaged by LPS that bypass the NEMO defect?

(4) Does this mean that TLR4-MyD88 NF-κB activation does not require NEMO? And if so could they show how this happen because the known published work argues against that.

Our data indicated that acute canonical NF-κB activation is not globally impaired in $NEMO^{DK}$ B cells. Both CD40- and LPS-induced canonical NF-κB activation are intact **(Figure 7A-B; new Appendix Fig. S1G**), arguing against a general defect in canonical signaling. To address the apparent discrepancy, we examined NEMO monoubiquitination. As shown in the **new Figure 8D**, LPS stimulation does not induce NEMO monoubiquitination, whereas CD40 stimulation does. This demonstrates that NEMO monoubiquitination is a CD40-specific signaling feature, not a universal requirement for NF-κB activation. Consistent with this, transcriptional analysis by the MAGIC algorithm **(Appendix Fig. S3B-C**) showed that RelA/NF-κB dependence differs between CD40 and LPS programs, suggesting distinct downstream requirements. These data support a stimulus-specific mechanism. The $NEMO^{DK}$ mutation disrupts a CD40-dependent pathway that requires NEMO monoubiquitination, whereas LPS signaling remains intact because it does not rely on this modification.

5. This selective defect in CD40 but not LPS responses should be more thoroughly investigated and mechanistically explained. Ideally, the authors should provide side-by-side signaling and functional assays comparing CD40 and LPS stimulation in WT and mutant B cells, including measurements of NF-κB activation (e.g., p-p65, p-IKK). Moreover, authors should explain how LPS differentially activate MyD88-dependent signalling or TRIF-dependent signalling in their system.

As described above, we now show that LPS fails to induce NEMO monoubiquitination, unlike CD40 stimulation in B cells. This clarifies why only CD40-induced RelA activation is defective and LPS-induced NF-κB signaling is intact in $NEMO^{DK}$ B cells. Our data support a model in which only CD40 stimulation causes mROS-induced NEMO monoubiquitination while conventional canonical NF-κB signaling induced by both CD40 and LPS are intact in mutant B cells. Data shown in **new Appendix Fig. S5B** showed that

other stimuli tested do not activate NF-κB as robustly as CD40 at 48 hours post-stimulation in WT cells. Thus, sustained and robust NF-κB activation appear to be unique to CD40 stimulation condition in B cells.

Figure 3:

6. This figure overall, highlights a notable decrease in RelA in NEMODK mutant, alongside suppression of key DNA damage response genes such as Trp53, Brca1, and Atg7. This widespread transcriptional repression, especially involving pro-survival and stress response genes, would conventionally be expected to render cells more susceptible to impaired viability. Yet, the authors report no measurable increase in cell death, nor do they investigate compensatory mechanisms that might preserve cell survival. This raises two critical questions:

(1) If one of the most pivotal pro-survival pathways is impaired, why is there no compensatory upregulation of alternative survival or stress-response pathways (e.g., autophagy-related genes)?

(2) How do cells maintain viability despite such broad molecular defects in pro-survival signaling?

The lack of detectable differences in viability and apoptosis measurements between WT and $NEMO^{DK}$ B cells (**Fig. EV3D-F**) was also quite surprising to us. However, based on the analysis of transcriptomic differences, we believe that acute canonical and delayed noncanonical NF-κB signaling pathways, which remain intact in $NEMO^{DK}$ B cells (**Figure 7A-C**), may contribute to the survival of CD40+IL-4 stimulated B cells. Thus, there is no measurable difference in survival between WT and $NEMO^{DK}$ B cells. Instead, our data suggest that the “third” pathway induced specifically by CD40 stimulation, which involves mROS-NEMO monoubiquitination-RelA signaling, contributes to ICAM-1 expression, cell cycle, DNA replication, and DNA damage repair processes, including AID and NBS1 induction, to promote HA formation, cell proliferation, CSR, and antibody-secreting cell generation.

7. Furthermore, the functional implications of this molecular repression remain underexplored. While the authors note that the mutant mice are viable, the immune signature of these NEMO-deficient cells is not clearly defined, particularly in terms of inflammatory cytokine storm. The manuscript refers to functional antibody defects, but does not address the downstream consequences of impaired humoral immunity. Are these mice more susceptible to infections? Is there altered vaccine responsiveness, or reduced affinity maturation? A more detailed immunophenotypic analysis and discussion are required.
All of the omics data are at the 48-hour time point. The authors should do (or at least report on) the kinetics of these transcriptional differences. When after stimulation do WT and NEMODK gene expression profiles diverge? The NF-κB activity data (Fig. 5) are consistent with diverging as early as a few hours and are evident by 24-48 h.

These are indeed valid points raised by the referee. Given the limited number of $NEMO^{DK}$ mice available to us, we could not perform all the requested experiments. However, as indicated above, we performed

additional LCMV-Armstrong infection (**new Fig. EV2B-E**). We also showed kinetic analyses of the expression of certain genes and encoded proteins critical to CSR, namely AID and NBS1, in **Figure 6B-C** and **Figure 6F-G**. These results showed significant reduction in the expression of AID and NBS1 in NEMO^{DK} B cells compared to WT at day 2 or 3 post stimulation, depending on the genes/proteins analyzed. We also performed single cell RNA sequencing analysis to further investigate the diversity of cell populations generated during *ex vivo* stimulation of WT and NEMO^{DK} B cells. Data shown in **new Figure 4E-H** revealed that B cell differentiation from naïve state to antibody-secreting cell (ASC) state is progressively diminished in NEMO^{DK} B cells relative to WT cells. These insights into the kinetic alterations in both gene/protein expression and cellular states in NEMO^{DK} B cells help explain the defects in humoral responses in these mutant B cells.

Figure 4:

8. This figure presents data on the expression of NBN and AID, both of which are well-known players in the DNA damage response and class-switch recombination (CSR). A direct mechanistic link is missing that ties their downregulation to NEMO-specific ubiquitination defects or downstream functional outcomes. It would significantly strengthen the manuscript if the authors clarified (1) how their regulation is influenced by NEMO mutation/ dysfunction, and (2) what functional consequences arise from their suppression (dependent of NEMO mutation), particularly regarding CSR.

We further investigated how NEMO double lysine mutation might directly affect the induction of *Nbn* gene following CD40+IL-4 stimulation. We found that RelA is recruited proximal to the *Nbn* transcriptional start site by CUT&Tag analysis (**new Figure 6H**). We further demonstrated the presence of 4 putative NF-κB binding sites proximal to the *Nbn* start site (**new Figure 6I**). We also demonstrated that of the 4 DNA-protein complexes are formed on these putative κB sites (**new Figure 6J**), 3 of which are specific (**new Fig. EV4A**). We also showed that RelA is a component of these CD40+IL-4 inducible nuclear κB complexes (**new Fig. EV4AB**). Finally, we demonstrated that by chromatin immunoprecipitation followed by qPCR analysis, RelA is inducibly recruited to two of these *Nbn* κB sites in WT B cells but not in NEMO^{DK} B cells following CD40+IL-4 stimulation (**new Figure 6K**), thus demonstrating NEMO double lysine specific RelA recruitment to the *Nbn* locus. Expression of both AID (**new Figure 6A**) and *Nbn* (**new Figure 6E**) is not altered in NEMO^{DK} B cells following LPS+IL-4 stimulation, thus further demonstrating the CD40-specificity of defects seen in NEMO^{DK} B cells.

It is reported in the literature that haplodeficiency in both AID$^{+/-}$ and NBS1$^{+/-}$ mice causes approximately ½ the production of class-switched antibodies (Reina-San-Martin *et al*, 2005; Jiang *et al*, 2009). Since the levels of both AID and NBS1 are ~1/2 reduced in NEMO^{DK} B cells specifically following stimulation with CD40+IL-4 relative to WT B cells (**Figure 6 A-G**), we did not investigate whether such reductions specifically

lead to reduced CSR, since we already demonstrated the ~1/2 redution in IgG1 antibody response in NEMO^{DK} B cells following stimulation with CD40+IL-4, but not LPS, relative to WT B cells (**new Figure 4D**).

9. As NBS1 and AID are stated to be downstream of NF-κB RelA, the authors need to support this assertion with proofs of direct interaction. Aside from Nbn and Aicda, are there any other key NF-κB target genes left to be validated? The authors can check genes that were also present in their screen.

As stated above, we have validated the direct recruitment and binding of RelA in *Nbn* κB elements (**new Figure 6H-K**). We did not pursue *Aicda* as this gene has been previously demonstrated to be a direct NF-κB target gene. Given that reductions in these two genes and proteins can explain the reduced CSR as stated above, we did not pursue additional potential targets. The gene expression data, along with all other transcriptomic and epigenomic data will be deposited to the journal for others to evaluate if they wish to do so.

Figure 5:

10. In Figure 5, specifically in panels 5A, D and G, EMSAs are presented to assess NF-κB activation following CD40 stimulation. Immunoblots for phosphorylated p65 (RelA) should also be performed as a more direct measure of canonical NF-κB pathway activation.

Since (1) these biochemical studies require pooling of multiple spleens to isolate sufficient B cells for analysis and EMSA DNA binding assay, (2) we have a limited number of matched WT and NEMO^{DK} mice, and (3) we considered phospho-RelA immunoblot assays are mostly redundant in information generated with EMSA analyses, we did not perform phospho-RelA western blot analysis. We prioritized performing other experiments described above and elsewhere due to a very limited number of NEMO^{DK} mice available to us.

11. Furthermore, the quantified differences shown in Figure 5E are not clearly reflected in the EMSA images in 5D. The band shifts are faint, do not show NF-kB dimers and do not convincingly demonstrate a dynamic difference in NF-κB activity between wild-type and mutant cells. The reported divergence at later time points (3-6 hours) raises mechanistic questions about the temporal regulation and feedback loops within the NF-κB pathway, issues that the authors do not sufficiently address. To strengthen this figure, we strongly recommend the inclusion of immunoblots for phosphorylated p65 (p-RelA) and phosphorylated IKKα/β. We also suggest inhibiting IKKs using an inhibitor to neutralize the effect shown to assert specificity of the difference in activation. It is known that canonical NF-κB complex could be either p50-p65 heterodimer or p50-p50 homodimer, both of them have different effect on downstream signalling. It would be of value for the authors to comment on this, employing western blots showing the phospho-p105 and p50 product.

Given that there are noncanonical NF-κB complexes present in later time points which are not different between WT and NEMO^{DK} B cells as shown in **Figure 7C**, we believe that the presence of these complexes obscures the difference of canonical NF-κB complexes between WT and NEMO^{DK} B cells at later time points. Western blots shown in **Figure 7E** clearly show reduced phospho-IκBα (target of phospho-IKKα/β) and consequent reduction in the level of IκBα due to IKK-induced proteasomal degradation. As stated above, these biochemical analyses require pooling of multiple spleens from mutant mice, which were quite limiting, and the information generated are mostly redundant, we did not prioritize performing additional phospho-RelA and phospho-IKKα/β biochemical analyses.

12. The authors showed extensively in figure 3 that RelA is transcriptionally downregulated in response to CD40 in NEMODK mutant, however in figure 5F, the protein expression levels of RelA are equal amongst NEMOWT and NEMODK mutant. What is authors´ comment on this?

The levels of RelA/p65 are not altered in NEMO^{DK} B cells as shown in multiple cases (e.g., **Figure 7E**, **Figure 8E**). It is the activation and activity of RelA that is altered. To avoid confusion, we have now stated this in the Result section "**RelA protein levels did not change (e.g., Figure 7E). Thus, RelA activation, rather than its expression, is reduced in CD40-stimulated NEMO^{DK} B cells**" (**page 17, lines 16-17**).

13. In figure 5G the authors present data showing NF-κB signaling activation upon CD40 stimulation for up to 4 days, but do not consider/validate the half-life or turnover of the CD40 receptor. In vivo, CD40-CD40L interactions are transient. The authors should clarify:

• What is the expected half-life of CD40 on B cells under their experimental conditions?
• Is CD40 expression monitored over the 4-day time course?
• Could prolonged CD40 engagement lead to secondary effects unrelated to canonical signaling, such as altered cell viability or exhaustion?

To address these concerns, the authors are advised to provide time-resolved CD40 surface expression data.

We show that similar surface expression of CD40 in naïve B cells from WT and NEMO^{DK} B cells (**new Fig. EV3B**). We further show that expression of CD40 is not altered during the course of study (**new Fig. EV3C**). We chose to use immunoblot, instead of surface staining of CD40 and FACS analysis, due to the presence of anti-CD40 antibody on B cells used for stimulation. This analysis demonstrated that the levels of CD40 are not different between WT and NEMO^{DK} B cells.

Figure 6:

14. In figure 6A, the authors show that upon CD40 stimulation, NEMOWT get mono-ubiquitinated whereas NEMODK mutant does not. To confirm this, it is necessary to perform an immunoprecipitation for ubiquitin

and present immunoblot for NEMO. Also, as we are looking for changes in post-translational modifications in NEMO WT vs DK, it is important to include full-length blots of NEMO and ubiquitin.

We have performed such IP-immunoblot experiments earlier, but we were unable to obtain reliable results due to the presence of a large amount of IgG (~50ka) used for immunoprecipitation in the subsequent immunoblot analysis, which obscured the similarly migrating monoubiquitinated NEMO (~50kDa). This was the reason why we had to perform *in vitro* deubiquitination and desumoylation assays in the first place. We now include such an explanation in the Results section "**To test this, we first performed immunoprecipitation of ubiquitin in denatured protein extracts, followed by Western blot analysis of NEMO. However, the presence of large amounts of Ig heavy chain (50 kDa) that comigrated with modified NEMO obscured our analysis. Thus, we next performed *in vitro* deconjugation assays…**" (**page 18, lines 10-14**) to clarify the rationale for performing such experiments.

15. Figures 6C and D aim to demonstrate that CD40 stimulation in 70Z/3 cells stably expressing mCD40 induces mono-ubiquitination of NEMO. However, the message is redundant across the two figures. Figure 6C, showing the 24-hour stimulation time point, presents the data with clearer and better-resolved blots, making it easier to interpret. In contrast, Figure 6D attempts to show a time course, but the blots are poor in quality, with weak and poorly distinguishable bands that do not add substantial new insight beyond what is already conveyed in 6C. We suggest that Figure 6C be retained in the main manuscript, while Figure 6D be either redone with improved blot quality and quantification or moved to the supplementary material.

We have now eliminated the data from reconstituted 70Z/3 cells as pointed out here such results do not add substantially to the overall results presented (especially in view of many new data results added).

16. In Figure 6G it looks as if the labelling order for the enzymes is incorrect or interchanged, please verify. In principle, DUB (USP2) + should cleave the K63 chains and SENP1 + should cleave SUMO chains.

Thank you for pointing out this mistake. It is now corrected.

Referee #2:

In the present manuscript, Miyamoto and colleagues generated knock-in (KI) mice to analyze the in vivo role of NEMO lysines 270 and 302. Sumoylation or mono-ubiquitination of these lysines has been shown to drive NF-kB activation upon DNA damage response (e.g. ionizing radiation or genotoxic agents). NEMO DK mice, with conservative KK270/302RR exchanges, are viable. A decrease in testes size (not further analyzed) and a reduction in IgA and IgG1 levels is the main phenotypic alteration under homeostatic conditions. Humoral immune response upon LCMV infection are compromised. The authors show mildly impaired CD40-triggered B cell activation in vitro (proliferation, IgG1 class switching), while BCR and LPS stimulation remained unchanged. Transcriptomic and molecular profiling suggested that sustained activation of NF-kB RelA is reduced in NEMO DK mice. NEMO mono-ubiquitination in response to CD40 stimulation is absent in NEMO DK B cells. CD40 also induces reactive oxygen species (ROS) and blockade of this the antioxidant N-acetyl cysteine (NAC) abrogates in vitro CD40-induced mono-ubiquitination, NF-kB activation and IgG1 class switching.

Overall, the study describes the first attempt to unravel an in vivo role of lysines 270/302 in NEMO. It describes some interesting and unexpected effects on impaired CD40 stimulation in NEMO DK B cells. However, the underlying mechanism is unclear. Further, in vitro NEMO modifications at lysines 270 and 302 have been connected to NF-kB activation after DNA-damage response, but there has been no attempt to test this in the physiological in vivo setting.

Major concerns:
There are mild effects on CD40 stimulation, but lysines 270/302 in NEMO do not seem to be main drivers of CD40 responses. The lysines rather modulate sustained responses (pro-longed RelA activation), which could arise - as the reviewers note in the discussion - from indirect events such as DNA damage which is of course happening in B cells undergoing class switch recombination and somatic hypermutations. The Miyamoto laboratory reported pioneering findings on the unconventional role of NEMO and lysines 270 and 302 in DNA damage-induced NF-kB activation. It is unclear why the study focusses on rather mild alterations in B cells, while potential changes in DNA damage responses have not been investigated. What happens if these mice are exposed to ionizing radiation or chemotherapeutic agents? Can tumors be induced and are therapeutic responses to DNA-damaging reagents in NEMO DK mice altered?

We have now included data on how $NEMO^{DK}$ mice and tissues/cells respond to DNA damage induced by ionizing radiation (**new Figure 1A-E**). We provided evidence that whole body irradiation of $NEMO^{DK}$ mice demonstrated compromised NF-κB activation in certain tissues, such as liver and kidney (**new Figure 1A-C**). Because Bredemeyer et al. (Nature 2008) demonstrated the activation of ATM-NEMO-NF-κB pathway in response to physiological DNA double strand breaks induced during V(D)J recombination as well as

exogenous radiation in pre-B cells, we also investigated how NEMO^{DK} pre-B cells would respond to radiation. Both NF-κB activation (**new Figure 1D**) and target gene induction (**new Figure 1E**) are inhibited in NEMO^{DK} pre-B cells. These results demonstrated that NF-κB activation in response to radiation is defective in certain NEMO^{DK} mice and tissues/cells. Because tumor studies involve long-term follow up studies and we had a limited number of mice to address other major concerns of three reviewers, we used available NEMO^{DK} mice to address such concerns and did not perform tumor studies in the current study. We will be conducting tumor-related investigations in future studies.

Basal IgG1 and IgA levels are reduced in NEMO DK mice. Instead of only testing B cell responses upon LCMV infection, it will be important to see in how antibody class switching, and antigen-specific immune responses are altered upon immunization with T cell-dependent an/or -independent antigens. Is the defect indeed at the GC reaction, which elicits less plasma cells and reduced humoral immune responses? Is this connected with an impaired DNA damage response in GCB cells undergoing class switching and somatic hypermutations?

While questions of TD and TI humoral responses are important ones, due to the time required for the alteration of our animal protocols, as well as the limited availability of NEMO^{DK} mice, we were unable to perform such studies during the revision period. We hope that reduced class-switched antibody production following LCMV infection in NEMO^{DK} mice relative to WT mice (**Figure 2G**) addresses the physiological importance NEMO double lysine sites for antibody production *in vivo*.

We were able to address whether DNA damage signaling *per se* was required to mediate NEMO monoubiquitination and induction of critical factors, such as NBS1, in B cells following CD40+IL-4 stimulation *ex vivo* by using primary B cells derived from commercially available ATM$^{-/-}$ mice and **newly derived NEMO^{S85A} mice**. We also measured the induction of DNA damage in longer-term stimulation conditions. While DNA damage was indeed induced at longer time periods (**new Fig. EV5C**), induction of NEMO monoubiquitination and NBS1 protein was intact in stimulated ATM$^{-/-}$ and NEMO^{S85A} B cells (**new Figure 8E** and **new Fig. EV5E**). Consistent with this lack of impact of DNA damage signaling, HA formation was also not perturbed in ATM$^{-/-}$ and NEMO^{S85A} B cells (**new Fig. EV5D**). These results together demonstrated that the DNA damage-ATM-NEMO signaling pathway is not involved in CD40+IL-4 induction of sustained NEMO monoubiquitination.

In figure 7 the authors show that CD40 induces ROS induction. NAC treatment prevents B cell responses to CD40, but the connection to the previous data is unclear. ROS induction is not altered in NEMO DK cells. Despite the correlative observation that high concentrations (50 mM) of NAC (a strong antioxidant) abolish NEMO mono-ubiquitination and NF-kB activation as well as antibody class switching, there is no evidence that this is linked to the much milder effects observed in NEMO DK mice. It is well established that IKK/NF-

kB signaling is highly sensitive to the redox status of the cells and most likely all NF-kB activating pathways are affected by 50 mM NAC. Again, why have NEMO DK B cells (or other cells) not been subjected to DNA-damage reagents that induce NF-kB?

As correctly pointed out, the concentration of NAC needed to observe strong inhibitory effects on NEMO monoubiquitination and downstream events was rather high. When a lower concentration of NAC (25 mM) was used, weaker impacts were observed. Thus, to independently address the origin of ROS following CD40+IL-4 stimulation, we now provide additional data (**new Figure 9**) investigating the known sources of ROS following such stimulation. The new results together support the role of mitochondrial ROS as the major source (new Figure 9C-D) that promotes HA formation (new Figure 9B), NEMO monoubiquitination (new Figure 9E), NF-κB activation (new Figure 9F), and class switching (**new Figure 9G**). In these studies, the level of inhibition of such intermediate steps was generally closer to that observed in $NEMO^{DK}$ B cells.

Specific points:

1) NF-kB signaling has been implicated in many processes of B cell differentiation. Are there any changes in marginal zone or follicular B cells? Is the ratio of B1(a/b) to B2 cells affected?

We did not observe any evident defects in marginal zone (**Fig. EV1D**) or follicular B cells (**Fig. EV1C**) in $NEMO^{DK}$ mice. Similarly, analysis of peritoneal samples did not reveal clear defects in B1a or B1b cell populations (see figure below, y-axis: % of cells). However, as these analyses were performed on only two mice per group, we chose to not include these data in the manuscript.

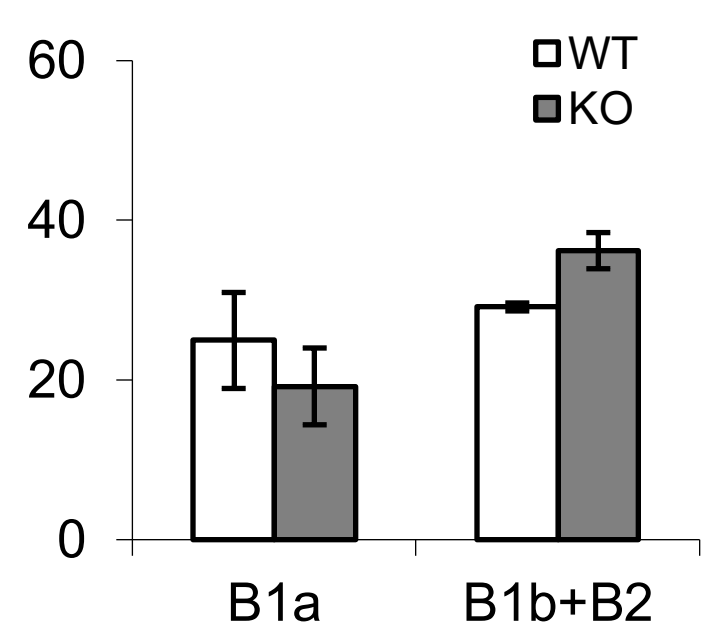

2) How severe is the defect of NEMO DK in coping with LCMV infection. Despite failing to mount optimal LCMV-specific humoral immune response, are NEMO DK mice unable to control viral titers or are they more easily succumbing to LCMV infection?

We now performed additional LCMV infection experiments to address this question. We showed that $CD8^+$, $CD4^+$ and T_{FH} responses to viral infection are normal (**new Fig. EV2B-D**) and viral clearance, which is

largely mediated by CD8^{+} T cells, is also normal (**new Fig. EV2E**) in NEMO^{DK} mice.

3) Are plasma cells reduced after LCMV infection in NEMO DK mice?

We addressed this question by first examining IgG1^{+} B cells following LCMV infection and found a significant reduction in this population in NEMO^{DK} mice (**Figure 2F**). While IgG1^{+} cells are not exclusively plasma cells, they reflect class-switched B cell responses that give rise to antibody-secreting cells. To more directly assess plasma cell differentiation, we performed single-cell RNA sequencing on CD40+IL-4-stimulated B cells. This analysis demonstrated a clear reduction not only in GC B cells but also in antibody-secreting cells (ASCs), including plasma cells, in NEMO^{DK} B cells (**new Figure 4E-G**).

4) CD40 can boost BCR-induced NF-kB activation and cell proliferation. Has the combination of CD40 and BCR stimulation been tested on cell proliferation and class switching?

Using Viafluor 405 to look at cell proliferation, which decreases in intensity with every round of cell division. The greater the proliferation, the greater the decrease in the Viafluor fold change compared to untreated controls. Proliferation in all samples began on day 2, with CD40+IL-4-WT cells undergoing the most rounds of division. As shown below, CD40+IgM-stimulated WT and NEMO^{DK} B cells proliferated less than their CD40+IL-4-stimulated counterparts from days 2-4. Since we were only able to perform n=2 experiments, we did not include this result in the manuscript.

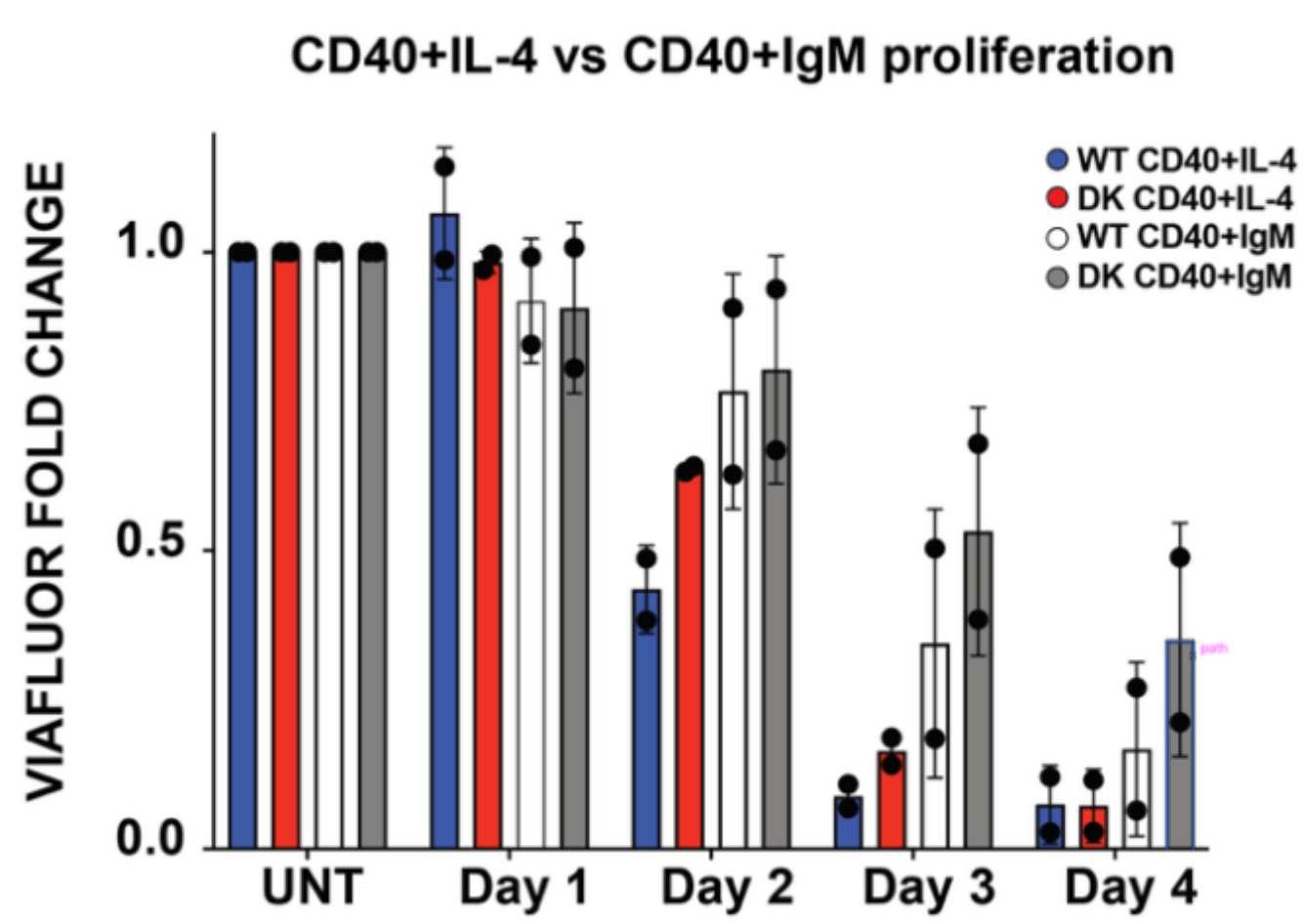

5) It seems that NEMO DK mice do not show an altered induction in the classical canonical or non-canonical NF-kB target gene signatures after CD40 stimulation. This is somewhat surprising given that RelA is the main transcription factor affected by NEMO DK. Have the authors closely looked at NF-kB target gene signatures?

We addressed this question by analyzing NF-κB target gene expression using a volcano plot, incorporating a curated gene set defined by Dr. Staudt's group at NIH (see below). This analysis revealed that numerous NF-κB target genes are differentially expressed in NEMO^{DK} B cells.

As the current figures already contain multiple subpanels, we did not include this analysis in the manuscript. However, we would be happy to incorporate it if the reviewer felt it would strengthen the study.

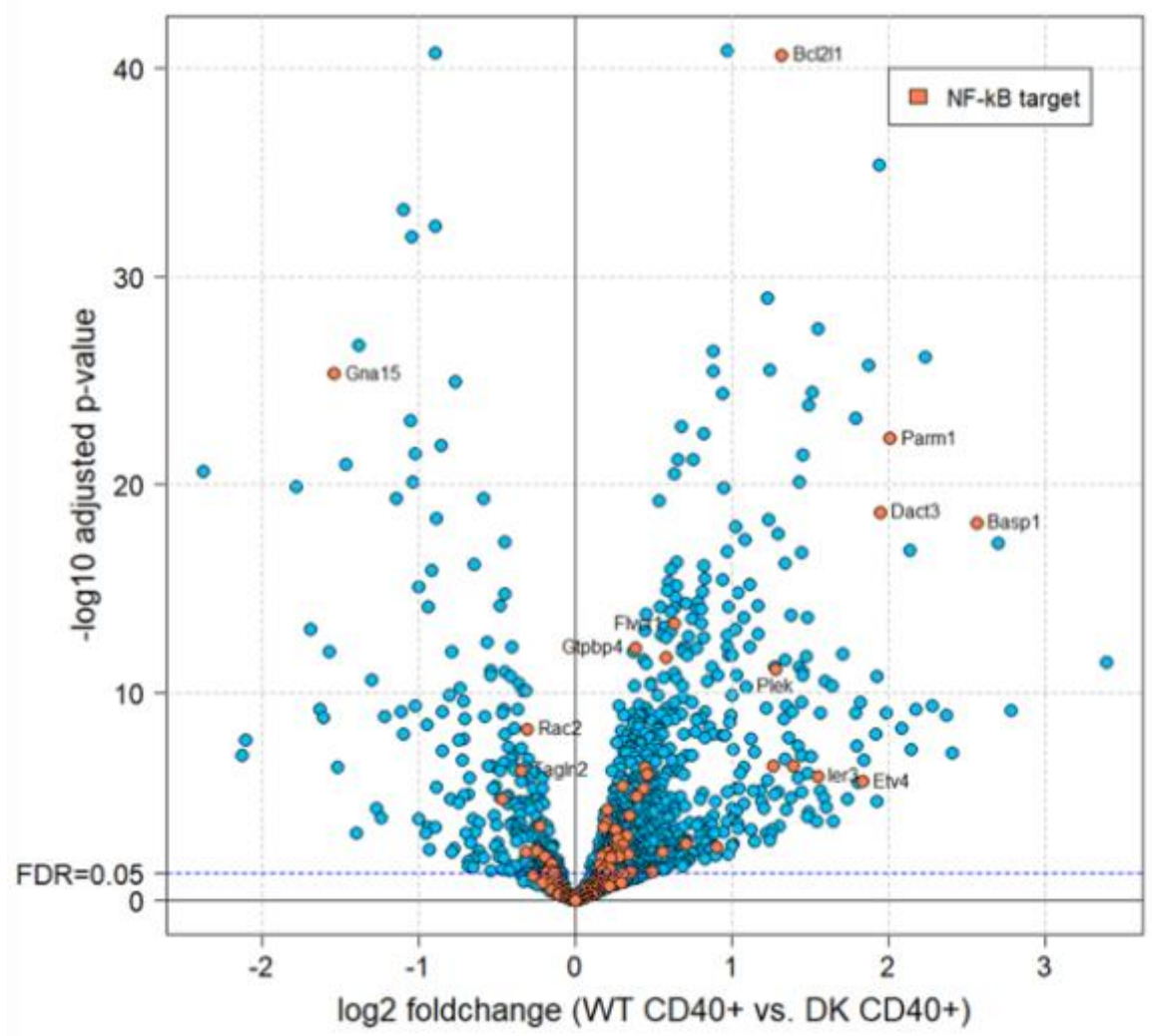

6) Fig. S5: In the aqua cluster CD40 and LPS responses are affected. Can the authors comment? Are there LPS-induced NF-kB target genes changed in this cluster?

Thank you for this important question. We have now included this analysis in the new **Appendix Fig. S3C.** Although the aqua cluster showed reduced gene expression under both CD40 and LPS stimulation, MAGIC analysis did not identify NF-κB family members as key drivers, suggesting these changes are not primarily due to impaired canonical NF-κB signaling. Consistently, while some LPS-induced genes are modestly reduced, they are not enriched for NF-κB targets, indicating that LPS-induced NF-κB responses remain largely intact. Additionally, we performed MAGIC analysis on the orange cluster, which showed LPS-inducible genes that are selectively reduced in NEMO^{DK} B cells (**Appendix Figure 3A**) and failed to identify NF-κB family members as dominant regulators (**Appendix Figure 3B**). Altogether, with the analysis of the purple cluster(**Figure 5D**), we interpreted the NEMO^{DK} B cell defects are caused by dysregulation in CD40-specific transcription, mediated by RelA-dependent mechanisms, and not solely explained by overall canonical NF-κB activity.

7) It should be investigated if the reduction in sustained RelA activation in NEMO DK cells is indeed specific for CD40 stimulation and not seen after LPS or BCR stimulation.

NF-κB activation is not sustained under LPS, BCR or other stimulation conditions even in WT B cells. An example is shown below, where WT B cells were stimulated with indicated stimuli at concentrations described in the manuscript for 48 hours. The major complexes observed in the CD40 and CD40+IL-4 are specific to κB binding sites (based on competition with excess cold WT or mutant κB sites). Note the lack of such complexes under LPS or LPS+IL-4 conditions. Another example comparing different stimuli are included in **new Appendix Fig. S5B**. Thus, given the very weak to almost no sustained activity under LPS or LPS+IL-4 condition, we did not perform a supershift assay to specifically evaluate RelA binding. These results further suggest that sustained NF-κB activity is quite unique to CD40 stimulation conditions in B cells.

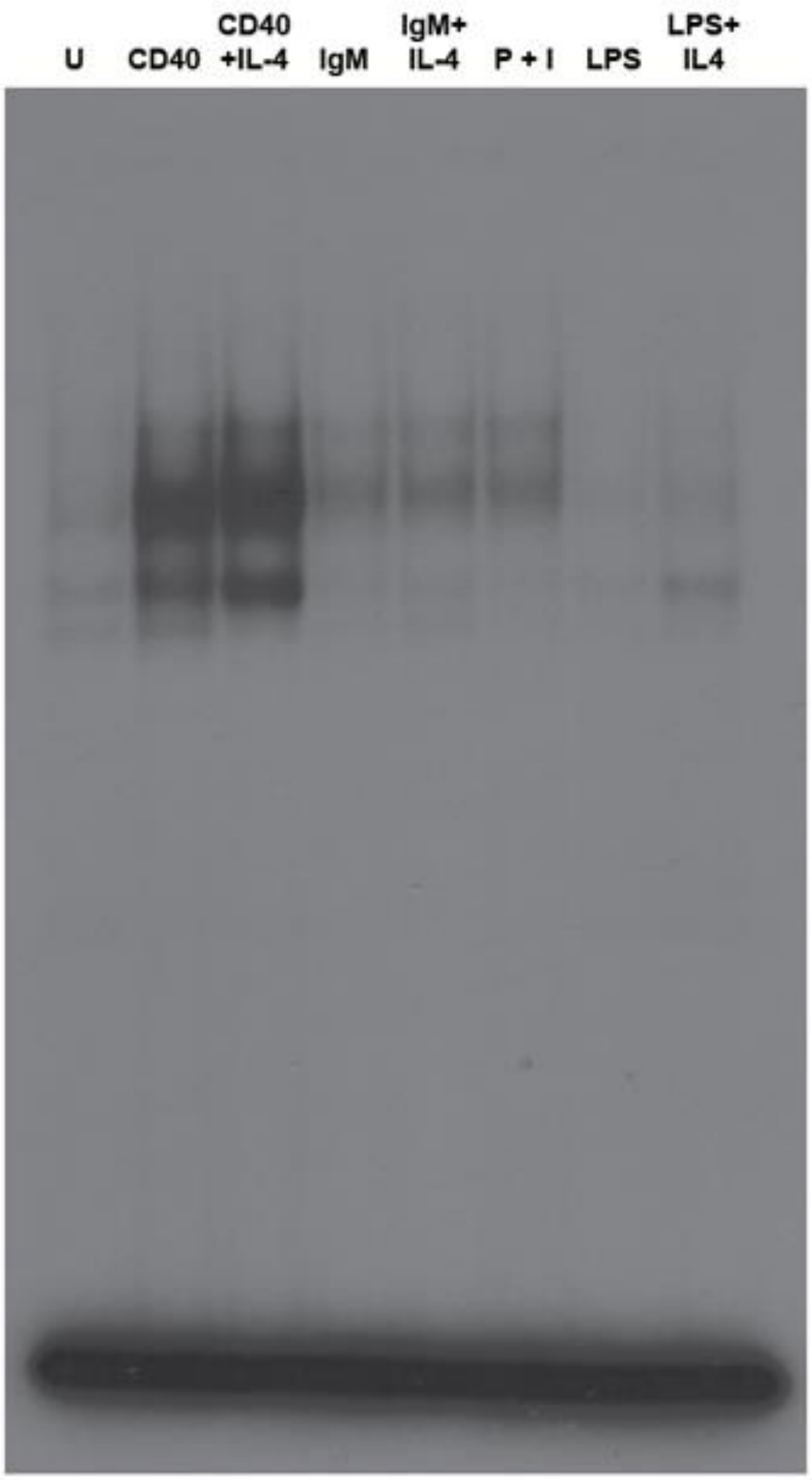

8) The antibody-supershift assay is not a good quantitative method to determine levels of active NF-kB subunits. NF-kB DNA-binding ELISA kits exist for each subunit, which is a much better way to quantify the relative changes in the various active NF-kB subunits.

While ELISA kits to detect all members of *human* NF-κB family members exist, we were unable to identify any commercially available ELISA kit that detects all members of *mouse* NF-κB family. Consequently, we were unable to address this question.

Minor points:
Fig. S2A: Indicate which gate corresponds to which B cell population.

Thank you for pointing out this deficiency. It is now described.

Fig. 2A: The presentation of cell cycle in this form is not very helpful.

This figure was now removed for clarity.

Fig. 4A seems to be misplaced and should be Fig. S7A. Subsequent labeling needs to be adjusted.

Thank you for pointing out this mistake. It is now corrected.

Referee #3:

This report from Li and colleagues presents intriguing findings to suggest that CD40-induced NEMO monoubiquitination (monoUb) on lysines residues 270 and 302 is required for sustained, ROS-dependent canonical NF-kB activation following CD40 engagement. The authors show that "NEMO-DK" mice, in which both lysines are switched to arginines, show a marked defect in CD40-induced B cell proliferation and class switch recombination (CSR), both in vitro and in response to LCMV infection in vivo. These functional deficits appear to be B cell-intrinsic and correlated with the absence of NEMO monoUb - this ROS-dependent post-translational modification is apparently required for sustained RelA activity and downstream transcription of genes required for cell cycle progression, DNA damage repair (e.g. NBS1) and CSR (e.g. AID), especially for IL-4-dependent IgG1 generation.

This manuscript is outstanding overall, providing convincing and comprehensive mechanistic evidence linking a novel NEMO PTM to prolonged canonical NF-kB activation required for B cell differentiation and function downstream of CD40 ligation. Their highly competent use of multiple next-generation sequencing modalities and bioinformatics analyses to inform these findings at the transcriptional level is particularly notable. Relatively inconsequential critiques outlined below should be addressed prior to publication.

We thank the reviewer for such positive comments.

Major critiques:

• One major question is how CD40 ligation triggers acute canonical NF-kB activation, considering CD40 signaling is traditionally associated with the noncanonical NF-kB pathway. The authors cite a single reference (Zarnegar et al), but this point could be elaborated on further in the introduction and discussion.

We have now included discussion of this signaling mechanism in the Discussion section (page 23, lines 29–31): "**Acute canonical NF-κB activation downstream of CD40 is driven by receptor-proximal signaling mediated by TRAF6 and TAK1, leading to rapid phosphorylation of IκBα within minutes of stimulation,**" with additional supporting references cited.

• Figure 2B-C: From the data presented, it may be premature to conclude that IgM-induced proliferation is normal in NEMO-DK mice - the agonistic Ab used appears to largely induce cell death based on the high proportion of sub-G1 apoptotic cells in their cell cycle analyses. B cell receptor-induced proliferation typically requires extensive crosslinking of the anti-IgM/D Ab to work properly, which may be worth trying here for a better comparison or at least acknowledging in the discussion.

Thank you for pointing this out. We have now deleted the cell cycle plot to avoid confusion and have included only Figure 4B, which highlights the %S/G_2/M fraction.

• Figure 2E-F: Why did the authors not measure IgG3 or IgG2b production in response to CD40 ligation (+ a relevant cytokine)? LPS-induced class switching to these isotypes appears comparable in WT and NEMO-DK mice but quite modest overall. This question seems potentially important, as Figure 1 presents a discrepancy between genotypes in relation to total IgG2/3 levels (1A) vs. LCMV-induced IgG2a/b levels (1H).

We wanted to demonstrate the CSR defect in CD40+IL-4 vs. LPS+IL-4. Since IgG1 is commonly induced under these conditions, we now include only the IgG1 response (**new Figure 4D**) to clarify and streamline the discussion.

• Discussion: the authors generally do a good job of considering study limitations, but they could elaborate further on putative ROS categories downstream of CD40 ligation that might be at play in stimulating NEMO monoUb, given their convincing data using the broad antioxidant NAC.

We now address this question by conducting additional experiments to investigate the major source of ROS that promotes NEMO monoubiquitination and downstream signaling and CSR reactions. We show that among the known sources of ROS under CD40 stimulation conditions, only mitochondrial ROS contributes to the ROS generation (**new Figure 9C-D**), HA formation (**new Figure 9B**), the NEMO monoquibiquitination (**new Figure 9E**), sustained NF-κB activation (**new Figure 9F**), and CSR (**new Figure 9G**). The overall magnitudes of effects of mROS inhibition align better with defects observed in $NEMO^{DK}$ B cells than a high dose of NAC, which was previously used and now included as **new Appendix Fig. S6A-G**.

Minor critiques:

• Page 7: the authors' description of BM chimera experiments presented in Figure 1 is somewhat confusing - it should be clarified in the text that WT and NEMO-DK BM were not tested together in a competitive experiment, but rather mixed 1:4 with mMT B cells as described in the methods.

We have now included a better description of this experiment on **page 8, lines 5-8**, "**In these chimeras, the bone marrow of either WT or $NEMO^{DK}$ mice was mixed at a 1:4 ratio with bone marrow from host µMT mice. This enabled specific evaluation of B cell-intrinsic defects as B cells are derived from either WT or $NEMO^{DK}$ mice while all other cell types remain normal**". We hope the reviewer agrees that the current description is easier to understand and that it clarifies the design of the experiment.

• Supp Fig 6C: although variable, H3K27 acetylation appears lower in NEMO-DK B cells - can these epigenetic marks be quantified in some way?

We have performed a statistical analysis and found that the difference was not statistically significant. This information is now included in **page 13, line 28-30**, "**signals for the histone modifications H3K4me3, H3K27ac, and H3K27me3 were statistically indistinguishable between WT and NEMO^{DK} B cells upon CD40+IL-4 stimulation (Supp. 7B-D)**".

• Figure 5C: it would be helpful to display the entire blot to view supershifted complexes on the EMSA

Although all figures from biochemical analyses will be included in the source data for the main figures per the journal's requirement, we have now specifically included this EMSA image as **new Appendix Fig. S5A**.

• Figure 6G: labels at the bottom of each blot appear to be switched

Thank you for pointing out this mistake. It is now corrected.

• Page 5: change "osteopetrosis" to "osteoporosis"

Thank you for pointing this out. It is now corrected.

• Page 9: change "changes in cell death does not account" to "do not account"

Thank you for pointing this out. This section is now rewritten.

Summary of new experiments included in the revised manuscript:

1. **Figure 1A-E** and **Appendix S1E-G**: This confirms the role of the double-lysine sites in murine NEMO in DNA damage-induced NF-κB signaling
2. **Expanded View 1E-F** and **Expanded View 2B-E**: This addresses thymocyte development and $CD4^+$ and $CD8^+$ T cell responses to LCMV-Armstrong infection and viral titer analysis, which together showed a lack of defects in T cell response to acute viral infection. (*Note: We have a T cell memory phenotype which will be discussed in a follow up study.)*
3. **Figure 3B-E**: homotypic aggregation (HA) quantification and statistical analysis. It also presents an analysis on ICAM-1 mRNA expression, which is a known NF-κB target gene necessary for HA formation. These new analyses clearly demonstrate the reduction in ICAM-1 mRNA expression and subsequent HA formation in $NEMO^{DK}$ B cells.
4. **Expanded View 3C**: This shows CD40 expression analysis over the 4-day time course, demonstrating the level of CD40 does not change during the analysis timeline.
5. **Expanded View 3D-F**: This shows cell death and apoptosis analyses, which show similar levels of these events in $NEMO^{DK}$ B cells.
6. **Figure E-H**: To address the question of antibody-secreting plasma cell vs germinal center cell generation, we also performed single-cell RNA sequencing analysis (scRNAseq). This analysis confirms a reduced production of not only GC cells but also antibody secreting cells (ASC) in $NEMO^{DK}$ B cells.
7. **Appendix S3B-C**: To address the specificity of the involvement of RelA dysregulation in $NEMO^{DK}$ B cells, we performed additional MAGIC analysis to identify transcription factors likely driving LPS-specific gene sets (orange cluster) and CD40 and LPS-specific gene set (aqua cluster). These analyses found the lack of RelA or any other NF-κB family member involvement in driving these gene sets and further confirmed that only CD40-specific gene set (purple cluster) is driven by RelA.
8. **Figure 6H-K** and **Expanded View 4A-B**: These address the mechanism of RelA-induced *Nbn* (encoding NBS1) gene induction. We show that Cut&Tag sequencing analyisis identified RelA signals surrounding *Nbn* start site. We further identified 2 major NF-κB binding sites within this region and show that RelA can directly bind to these sites by EMSA, EMSA competition and chromatin-IP assays. Since *Nbn* gene has not been previously shown to be an NF-κB target gene, all together these results provide the first evidence that *Nbn* is a direct RelA target gene in B cells under CD40+IL-4 stimulation condition.
9. **Appendix S5A-B**: These EMSA and supershift analyses show how CD40 is unique among various B cell stimuli to induce sustained NF-κB activation, in particular the RelA subunit.
10. **Figure 8D-E** and **Expanded View 5C-E**: These results provide evidence that DNA damage is induced in a sustained manner in B cells stimulated with CD40+IL-4; however, the analysis of $ATM^{-/-}$ and a newly derived $NEMO^{S85A}$ mice demonstrated that HA formation, NEMO monoubiquitination and NBS1 induction are all normal in these mouse models that would block DNA damage-induced NF-κB

signaling. Thus, these results clearly eliminated DNA damage as the intermediate signal induced by CD40+IL-4 to induce these B cell phenotypes.

11. **Figure 9A-G**: We have further investigated the source of ROS induced by CD40+IL-4, which mediates all of the B cell phenotypes. We showed when mitochondrial ROS is inhibited by Rotenone, HA formation, NEMO monoubiquitination, NF-κB activation and IgG1 class switching in CD40+IL-4 stimulated B cells is also subsequently diminished. Thus, these results further demonstrate that mitochondrial ROS is the major driver of these B cell phenotypes that are attenuated by the double-lysine mutation in NEMO.

Combined with the originally submitted data, these new results significantly bolster the initial conclusions drawn and substantially expand the understanding of the molecular mechanisms involved in ROS-induced NEMO monoubiquitination and its functional consequences. We believe that our study provides the first evidence for the presence of a "third" signaling pathway induced by CD40 stimulation of B cells, which specifically involves *sustained* (1) mitochondrial ROS generation, (2) NEMO lysine 270/302-dependent monoubuquitination and 3) RelA activation, and (4) downstream RelA-induced transcriptional changes that promote HA formation, proliferation, AID/NBS1 induction, and germinal center B cell and antibody secreting cell generation.

1st Revision - Editorial Decision **26th May 2026**

Dear Shigeki,

Thank you again for the submission of your revised manuscript (EMBOJ-2025-121503R) to The EMBO Journal and your patience during re-review. Your revision has now been seen by the three original referees, who had previously reviewed the first version of the manuscript, and we have received their comments, which are appended below.

As you will see, all three referees are satisfied with the depth and quality of the revision, and they find the new version of the manuscript substantially improved and their initially raised criticisms adequately and sufficiently addressed. In light of this input, I am happy to let you know that your manuscript has been accepted in principle for publication in The EMBO Journal.

There are only a few changes we need you to make in a final version of your manuscript before we can proceed with the next steps towards publication:

- Please note that our notifications to the e-mail address MelbaMarie.Tejera@moffitt.org of your co-author Melba M. Tejera could not be delivered. Please make sure that your co-author updates their profile in our system with a valid e-mail address, or send us please the current/new e-mail address so that we can update the account on your co-author's behalf.

- All funding information included in the Comments box in our online system must be provided instead using the "More Funders" option. Please make sure that this information matches the information provided in the Acknowledgements section of the manuscript.

- Thank you for depositing your sequencing datasets to the GEO database and for providing reviewer tokens for them. Please make sure that all datasets will be publicly available at the time of publication. The reviewer tokens can now be removed from the Data Availability statement of the manuscript. Instead, the specific accession ID, database, and specific URL to each dataset should now be provided in the Data Availability statement of the revised manuscript.

- Please change heading "Materials and Methods" to "Methods".

- Please change heading "Declaration of Interests" to "Disclosure and competing interests statement".

- The Author Contributions statement should be removed from the manuscript file. Instead, we use CRediT to specify the contributions of each author in the journal submission system. Please feel free to use the free text box to provide more detailed descriptions during submission. See also our guide to authors for more information: https://link.springer.com/partners/embo-press/editorial-policies#Authorship.

- As per our journal's policy, "data not shown" (on pages 16, 19, 25 and 27) is not permitted. All data referred to in the paper should be displayed in the main or Expanded View Figures, or in the Appendix. Please add these data or change the text accordingly if these data are not central to the study and its conclusions, or properly cite the respective published sources if these data can be found elsewhere.

- Expanded View Figures should be renamed to "Figure EV1-EV5" (instead of "Expanded view 1-5") in Figure legends.

- The Appendix file needs to be in PDF format; all Appendix Figures should be compiled in a single Appendix PDF file with each legend placed below its corresponding Figure; the title page should contain heading "Appendix for", followed by the manuscript's title and a Table of Contents including page numbers of all listed items; nomenclature should be "Appendix Figure S#" throughout the manuscript file and the Appendix PDF file.

- Please note that EMBO press papers are accompanied online by:
A) a short (2 sentences) summary of the findings and their significance,
B) 2-5 short bullet points highlighting the key results, and
C) a synopsis image in .jpg or .png format that is exactly 550 pixels wide x 300 pixels high. Please note that all text needs to be legible at this size.
Please upload this information along with your revised manuscript (the text for A and B should be provided in a separate Word file).

- Our data editors have checked your Figures and their legends, and raised the following queries. Please address all points below completely in your revised manuscript (all changes should be highlighted or "tracked"):
1. Please note that information related to "n" is missing in the legends of Figures 2D-F; 4F, 5C, EV1 B, EV2 B-E.
2. Please note that n=2 in Figures EV3 C, E. In such cases, the individual data points must be shown for the two replicates, and no statistics can be calculated or shown.
3. Please note that the error bars must be defined in the legends of Figures 1E; 2A, C, D, E, F; EV1 A-D; EV2 B-E.
4. Please define the annotated p values ****/***/**/* as well as provide the exact p-values for the same in the legends of Figures

4A, EV2 A, as appropriate.
5. Please provide the exact p-values in the legends of Figures 3D, 4B, 6C, F; 8D, 9G, EV4 B.

- The manuscript sections need to be named and ordered as follows: Title page - Abstract - Introduction - Results - Discussion - Methods - Data Availability - Acknowledgements - Disclosure and Competing Interests Statement - References - Figure Legends - main Tables (if there are any) - Expanded View Figure Legends.

- Please also note that as part of the EMBO Press transparent editorial process, The EMBO Journal publishes online a Peer Review File along with each accepted manuscript. This File will be published in conjunction with your paper and will include the referee reports, your point-by-point responses and all pertinent correspondence relating to the manuscript. Your Author's Checklist will also be published at the end of the Peer Review File. Please let us know in case you want to remove any data or figures from your point-by-point responses before they are published as part of the Peer Review File. Retaining unpublished data in the Peer Review File means that these count as published and that the Peer Review File would need to be referenced in future publications. Please let the editorial office know in case you want to remove any data from this file (contact@embojournal.org).

We look forward to seeing a final version of your manuscript as soon as possible. Please let us know if you have any questions and use this link to submit your revision: https://emboj.msubmit.net/cgi-bin/main.plex.

Best regards,

Ioannis

Ioannis Papaioannou, PhD
Editor, The EMBO Journal
i.papaioannou@embojournal.org

Referee #1:

In the revised version of their manuscript the authors have satisfactorily and adequately addressed all critical points raised in my review of the previous version of this manuscript.

Referee #2:

The authors have adequately answered my questions. Especially, the new data showing impaired NF-kB activation after DNA damage in NEMO DK mice in combination with experiments showing that CD40 stimulation is independent of the DNA damage pathway adds important new insights. Thus, I support acceptance and publication of the manuscript in EMBO J.

Referee #3:

The authors have done an excellent job of addressing critiques, including new experimental data that strengthens the revised manuscript considerably. The revised paper is now suitable for publication.

All editorial and formatting issues were resolved by the authors.

Dear Shigeki,

Congratulations on an excellent manuscript! I am very pleased to inform you that it has been accepted for publication in The EMBO Journal. Thank you for comprehensively addressing the initially raised referees' concerns and our editorial requests for changes and corrections.

Your manuscript will be processed for publication by EMBO Press. It will be copy edited and you will receive page proofs prior to publication. Please note that you will be contacted by Springer Nature Author Services to complete licensing and payment information.

You may qualify for financial assistance for your publication charges - either via a Springer Nature fully open access agreement or an EMBO initiative. Check your eligibility: https://link.springer.com/journal/44318/how-to-publish-with-us

Should you be planning a Press Release on your article, please notify the production team via this form in order to coordinate publication and release dates: https://forms.cloud.microsoft/e/eH4A8JVP6g

If you have any questions, please do not hesitate to contact the Editorial Office. Thank you for your contribution to The EMBO Journal. Working with you has been a pleasure!

Best regards,

Ioannis

Ioannis Papaioannou, PhD
Editor, The EMBO Journal
i.papaioannou@embojournal.org

*****
Please note that it is The EMBO Journal policy for the transcript of the editorial process (containing referee reports and your response letters) to be published as an online supplement to each paper. If you should prefer removal of any referee-only figures included in the point-by-point response(s), e.g. because they may still be used for future publication or because they have been reproduced from published work by others, please do let us know immediately via response email.
More information is available here: https://link.springer.com/partners/embo-press/editorial-policies#Peer%20review

** Click here to be directed to your login page: https://emboj.msubmit.net

EMBO Press Author Checklist

Corresponding Author Name: Shigeki Miyamoto
Journal Submitted to: The EMBO Journal
Manuscript Number: EMBOJ-2025-121503R

USEFUL LINKS FOR COMPLETING THIS FORM
The EMBO Journal - Author Guidelines
EMBO Reports - Author Guidelines
Molecular Systems Biology - Author Guidelines
EMBO Molecular Medicine - Author Guidelines

Reporting Checklist for Life Science Articles (updated January
This checklist is adapted from Materials Design Analysis Reporting (MDAR) Checklist for Authors. MDAR establishes a minimum set of requirements in transparent reporting in the life sciences (see Statement of Task: 10.31222/osf.io/9sm4x). Please follow the journal's guidelines in preparing your
Please note that a copy of this checklist will be published alongside your article.

Abridged guidelines for figures
1. Data
The data shown in figures should satisfy the following conditions:
- ➞ the data were obtained and processed according to the field's best practice and are presented to reflect the results of the experiments in an accurate and unbiased manner.
- ➞ ideally, figure panels should include only measurements that are directly comparable to each other and obtained with the same assay.
- ➞ plots include clearly labeled error bars for independent experiments and sample sizes. Unless justified, error bars should not be shown for technical
- ➞ if n<5, the individual data points from each experiment should be plotted. Any statistical test employed should be justified.
- ➞ Source Data should be included to report the data underlying figures according to the guidelines set out in the authorship guidelines on Data

2. Captions
Each figure caption should contain the following information, for each panel where they are relevant:
- ➞ a specification of the experimental system investigated (eg cell line, species name).
- ➞ the assay(s) and method(s) used to carry out the reported observations and measurements.
- ➞ an explicit mention of the biological and chemical entity(ies) that are being measured.
- ➞ an explicit mention of the biological and chemical entity(ies) that are altered/varied/perturbed in a controlled manner.
- ➞ the exact sample size (n) for each experimental group/condition, given as a number, not a range;
- ➞ a description of the sample collection allowing the reader to understand whether the samples represent technical or biological replicates (including how many animals, litters, cultures, etc.).
- ➞ a statement of how many times the experiment shown was independently replicated in the laboratory.
- ➞ definitions of statistical methods and measures:
  - common tests, such as t-test (please specify whether paired vs. unpaired), simple χ2 tests, Wilcoxon and Mann-Whitney tests, can be unambiguously identified by name only, but more complex techniques should be described in the methods section;
  - are tests one-sided or two-sided?
  - are there adjustments for multiple comparisons?
  - exact statistical test results, e.g., P values = x but not P values < x;
  - definition of 'center values' as median or average;
  - definition of error bars as s.d. or s.e.m.

Please complete ALL of the questions below.
Select "Not Applicable" only when the requested information is not relevant for your study.

Materials

**Newly Created Materials**	**Information included in the manuscript?**	**In which section is the information available?** (Reagents and Tools Table, Materials and Methods, Figures, Data Availability Section)
New materials and reagents need to be available; do any restrictions apply?	Yes	materials and methods, no restrictions

**Antibodies**	**Information included in the manuscript?**	**In which section is the information available?** (Reagents and Tools Table, Materials and Methods, Figures, Data Availability Section)
For **antibodies** provide the following information:<br>- Commercial antibodies: RRID (if possible) or supplier name, catalogue number and or/clone number<br>- Non-commercial: RRID or citation	Yes	materials and methods, reagents tools table

**DNA and RNA sequences**	**Information included in the manuscript?**	**In which section is the information available?** (Reagents and Tools Table, Materials and Methods, Figures, Data Availability Section)
**Short novel DNA or RNA including primers, probes**: provide the sequences.	Yes	materials and methods, reagents tools table

**Cell materials**	**Information included in the manuscript?**	**In which section is the information available?** (Reagents and Tools Table, Materials and Methods, Figures, Data Availability Section)
**Cell lines:** Provide species information, strain. Provide accession number in repository **OR** supplier name, catalog number, clone number, and/**OR** RRID.	Yes	materials and methods, reagents tools table
**Primary cultures:** Provide species, strain, sex of origin, genetic modification status.	Yes	materials and methods
Report if the cell lines were recently **authenticated** (e.g., by STR profiling) and tested for mycoplasma contamination.	Not Applicable	

**Experimental animals**	**Information included in the manuscript?**	**In which section is the information available?** (Reagents and Tools Table, Materials and Methods, Figures, Data Availability Section)
**Laboratory animals or Model organisms:** Provide species, strain, sex, age, genetic modification status. Provide accession number in repository **OR** supplier name, catalog number, clone number, **OR** RRID.	Yes	materials and methods
**Animal observed in or captured from the field:** Provide species, sex, and age where possible.	Not Applicable	
Please detail **housing and husbandry conditions**.	Yes	materials and methods

**Plants and microbes**	**Information included in the manuscript?**	**In which section is the information available?** (Reagents and Tools Table, Materials and Methods, Figures, Data Availability Section)
**Plants:** provide species and strain, ecotype and cultivar where relevant, unique accession number if available, and source (including location for collected wild specimens).	Not Applicable	
**Microbes:** provide species and strain, unique accession number if available, and source.	Yes	materials and methods

**Human research participants**	**Information included in the manuscript?**	**In which section is the information available?** (Reagents and Tools Table, Materials and Methods, Figures, Data Availability Section)
If collected and within the bounds of privacy constraints report on age, sex and gender or ethnicity for all study participants.	Not Applicable	

**Core facilities**	**Information included in the manuscript?**	**In which section is the information available?** (Reagents and Tools Table, Materials and Methods, Figures, Data Availability Section)
If your work benefited from core facilities, was their service mentioned in the acknowledgments section?	Yes	acknowledgements

Design

Study protocol	Information included in the manuscript?	In which section is the information available? (Reagents and Tools Table, Materials and Methods, Figures, Data Availability Section)
If study protocol has been **pre-registered, provide DOI in the manuscript**. For clinical trials, provide the trial registration number **OR** cite DOI.	Not Applicable	
Report the **clinical trial registration number** (at ClinicalTrials.gov or equivalent), where applicable.	Not Applicable	

Laboratory protocol	Information included in the manuscript?	In which section is the information available? (Reagents and Tools Table, Materials and Methods, Figures, Data Availability Section)
Provide DOI OR other citation details if **external detailed step-by-step protocols** are available.	Yes	Materials and methods

Experimental study design and statistics	Information included in the manuscript?	In which section is the information available? (Reagents and Tools Table, Materials and Methods, Figures, Data Availability Section)
Include a statement about **sample size** estimate even if no statistical methods were used.	Yes	Figure legends
Were any steps taken to minimize the effects of subjective bias when allocating animals/samples to treatment (e.g. **randomization procedure**)? If yes, have they been described?	Not Applicable	
Include a statement about **blinding** even if no blinding was done.	Yes	materials and methods
Describe **inclusion/exclusion criteria** if samples or animals were excluded from the analysis. Were the criteria pre-established?<br><br>If sample or data points were omitted from analysis, report if this was due to attrition or intentional exclusion and provide justification.	Yes	materials and methods
For every figure, are **statistical tests** justified as appropriate? Do the data meet the assumptions of the tests (e.g., normal distribution)? Describe any methods used to assess it. Is there an estimate of variation within each group of data? Is the variance similar between the groups that are being statistically compared?	Yes	materials and methods

Sample definition and in-laboratory replication	Information included in the manuscript?	In which section is the information available? (Reagents and Tools Table, Materials and Methods, Figures, Data Availability Section)
In the figure legends: state number of times the experiment was **replicated** in laboratory.	Yes	Figure legends
In the figure legends: define whether data describe **technical or biological replicates**.	Yes	materials and methods

Ethics

Ethics	Information included in the manuscript?	In which section is the information available? (Reagents and Tools Table, Materials and Methods, Figures, Data Availability Section)
Studies involving **human participants**: State details of **authority granting ethics approval** (IRB or equivalent committee(s), provide reference number for approval.	Not Applicable	
Studies involving **human participants**: Include a statement confirming that **informed consent** was obtained from all subjects and that the experiments conformed to the principles set out in the WMA Declaration of Helsinki and the Department of Health and Human Services Belmont Report.	Not Applicable	
Studies involving **human participants:** For publication of **patient photos**, include a statement confirming that consent to publish was obtained.	Not Applicable	
Studies involving experimental **animals**: State details of **authority granting ethics approval** (IRB or equivalent committee(s), provide reference number for approval. Include a statement of compliance with ethical regulations.	Yes	materials and methods
Studies involving **specimen and field samples:** State if relevant **permits** obtained, provide details of authority approving study; if none were required, explain why.	Not Applicable	

Dual Use Research of Concern (DURC)	Information included in the manuscript?	In which section is the information available? (Reagents and Tools Table, Materials and Methods, Figures, Data Availability Section)
Could your study fall under dual use research restrictions? Please check biosecurity documents and list of **select agents and toxins** (CDC): https://www.selectagents.gov/sat/list.htm	Not Applicable	
If you used a select agent, is the security level of the lab appropriate and reported in the manuscript?	Not Applicable	
If a study is subject to dual use research of concern regulations, is the name of the **authority granting approval and reference number** for the regulatory approval provided in the manuscript?	Not Applicable	

Reporting

The MDAR framework recommends adoption of discipline-specific guidelines, established and endorsed through community initiatives. Journals have their own policy about requiring specific guidelines and recommendations to complement MDAR.

Adherence to community standards	Information included in the manuscript?	In which section is the information available? (Reagents and Tools Table, Materials and Methods, Figures, Data Availability Section)
State if relevant guidelines or checklists (e.g., **ICMJE, MIBBI, ARRIVE, PRISMA**) have been followed or provided.	Not Applicable	
For **tumor marker prognostic studies,** we recommend that you follow the **REMARK** reporting guidelines (see link list at top right). See author guidelines, under 'Reporting Guidelines'. Please confirm you have followed these guidelines.	Not Applicable	
For **phase II and III randomized controlled trials**, please refer to the **CONSORT** flow diagram (see link list at top right) and submit the CONSORT checklist (see link list at top right) with your submission. See author guidelines, under 'Reporting Guidelines'. Please confirm you have submitted this list.	Not Applicable	

Data Availability

Data availability	Information included in the manuscript?	In which section is the information available? (Reagents and Tools Table, Materials and Methods, Figures, Data Availability Section)
Have **primary datasets** been deposited according to the journal's guidelines (see 'Data Deposition' section) and the respective accession numbers provided in the Data Availability Section?	Yes	Data Availability
Were **human clinical and genomic datasets** deposited in a public access-controlled repository in accordance to ethical obligations to the patients and to the applicable consent agreement?	Not Applicable	
Are **computational models** that are central and integral to a study available without restrictions in a machine-readable form? Were the relevant accession numbers or links provided?	Yes	materials and methods
If publicly available data were reused, provide the respective **data citations in the reference list**.	Yes	materials and methods, references
