# Supplementary material for: NF-κB/RelA signaling required for CD40-induced humoral immunity depends on specific NEMO lysine residues in mice
Source: EMBO J. 2026 Jul 30;45(17):6229–66. doi: 10.1038/s44318-026-00875-0 (PMC13534677; doi:10.1038/s44318-026-00875-0)
Supplement: Supplementary file 5 [file 44318_2026_875_MOESM3_ESM.zip › Figure 1/1A/1A Liver Oct EMSA.pdf]

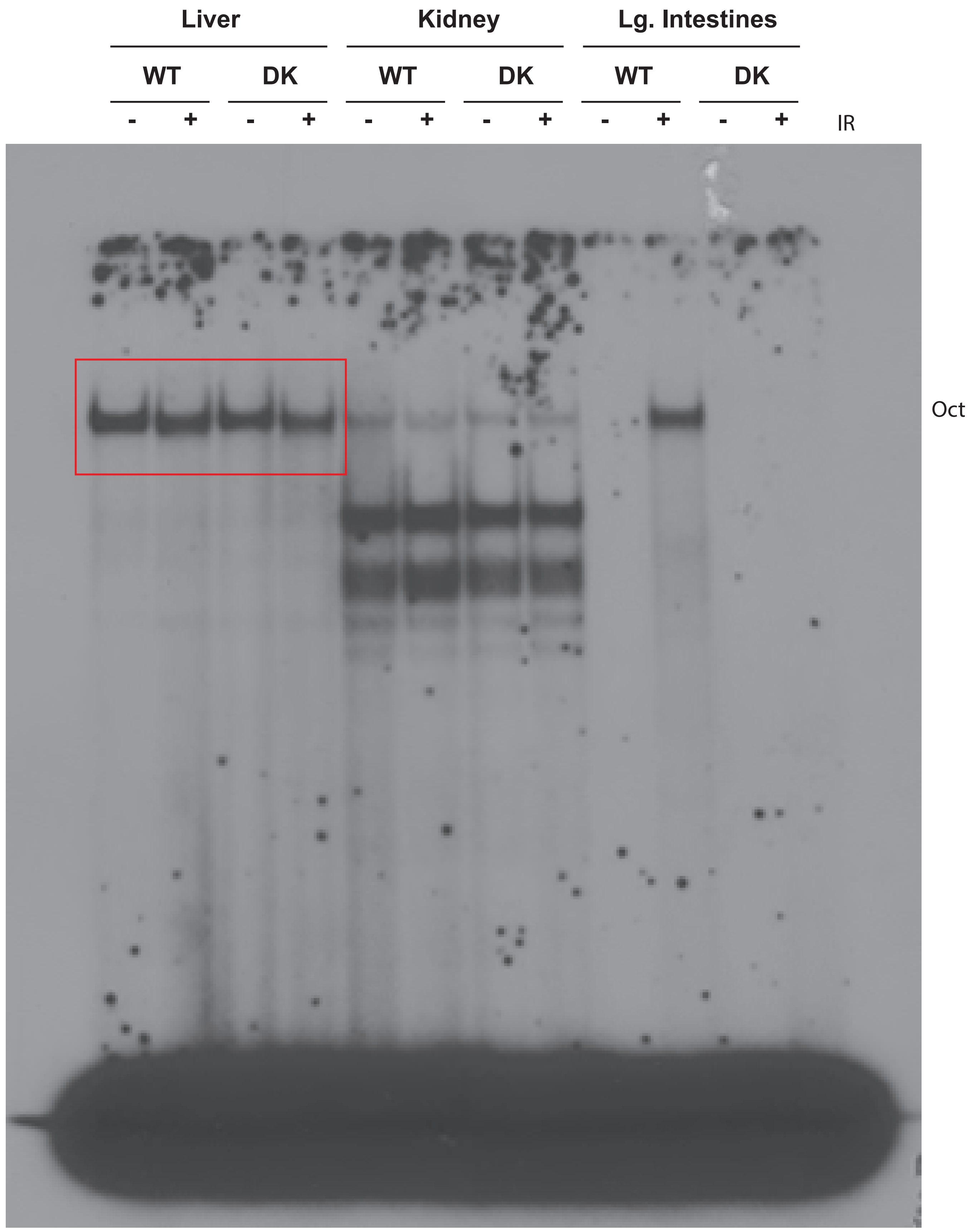
Liver
Kidney
Lg. Intestines
WT
DK
WT
DK
WT
DK
- + - + - + - + - + - +
IR
Oct
