# Supplementary material for: NF-κB/RelA signaling required for CD40-induced humoral immunity depends on specific NEMO lysine residues in mice
Source: EMBO J. 2026 Jul 30;45(17):6229–66. doi: 10.1038/s44318-026-00875-0 (PMC13534677; doi:10.1038/s44318-026-00875-0)
Supplement: Supplementary file 10 [file 44318_2026_875_MOESM8_ESM.zip › Figure 7/7E NEMO, Actin, RelA, IkBa immunoblot.pdf]

Figure 7E

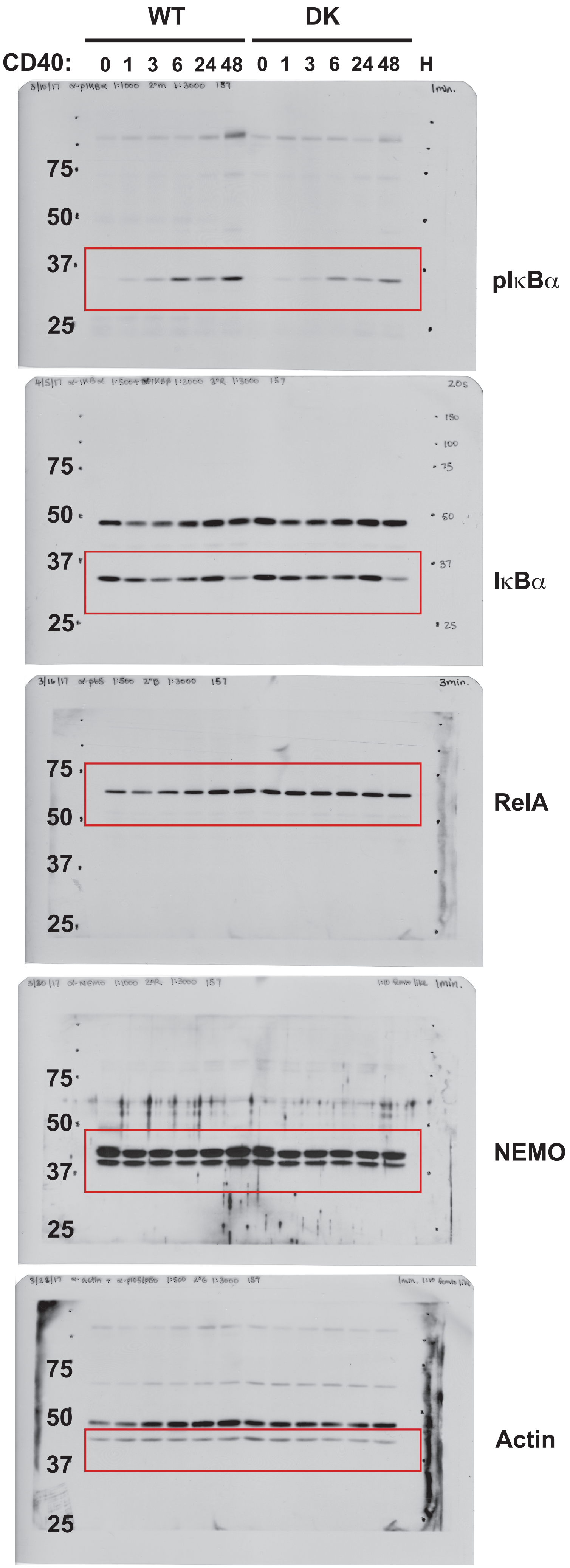

WT
DK
CD40: 0 1 3 6 24 48 0 1 3 6 24 48 H
75
50
37
25
pIκBα
75
50
37
25
IκBα
75
50
37
25
RelA
75
50
37
25
NEMO
75
50
37
25
Actin
