# Supplementary material for: NF-κB/RelA signaling required for CD40-induced humoral immunity depends on specific NEMO lysine residues in mice
Source: EMBO J. 2026 Jul 30;45(17):6229–66. doi: 10.1038/s44318-026-00875-0 (PMC13534677; doi:10.1038/s44318-026-00875-0)
Supplement: Supplementary file 11 [file 44318_2026_875_MOESM9_ESM.zip › Figure 8/8B/8B WEHI NEMO, RelA, actin.pdf]

Figure 8B

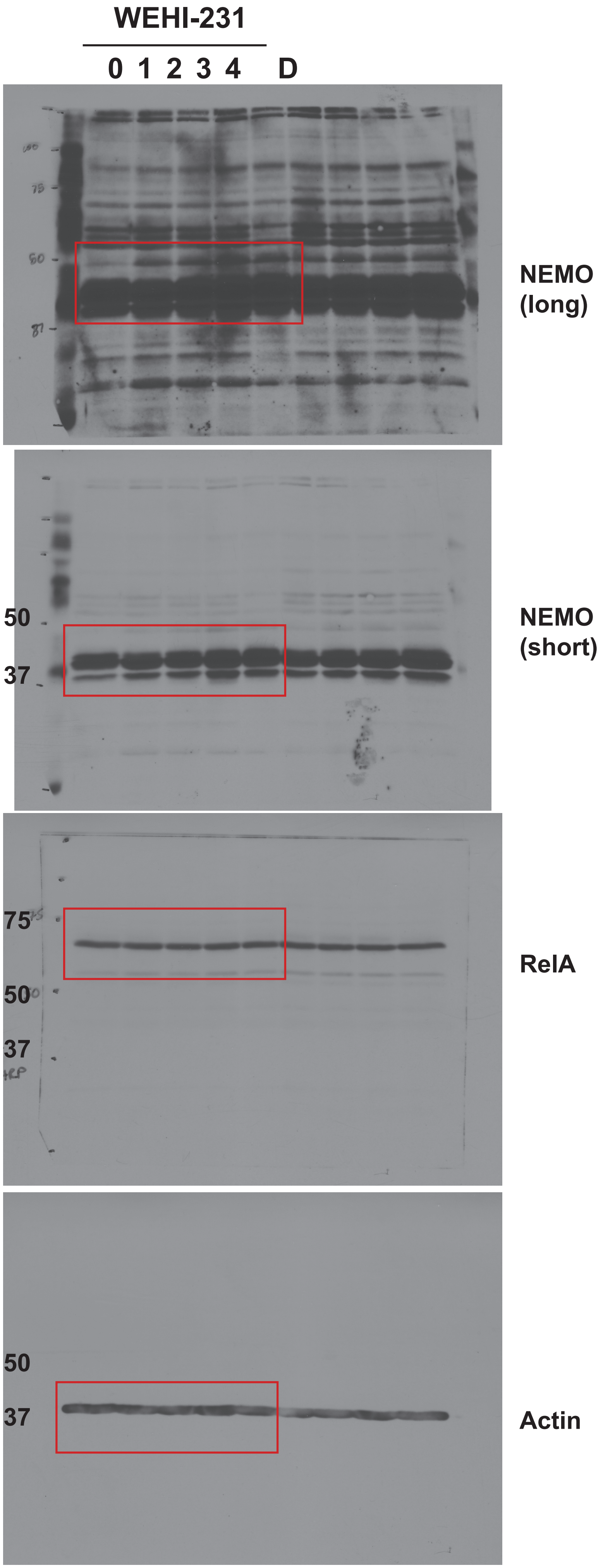

WEHI-231
0 1 2 3 4 D
NEMO (long)
50
37
NEMO (short)
75
50
37
RelA
50
37
Actin
